# Synthesis and Characterization of Triphenyl Phosphonium-Modified Triterpenoids with Never Reported Antibacterial Effects Against Clinically Relevant Gram-Positive Superbugs

**DOI:** 10.3390/pharmaceutics17121614

**Published:** 2025-12-16

**Authors:** Dafni Graikioti, Constantinos M. Athanassopoulos, Anna Maria Schito, Silvana Alfei

**Affiliations:** 1 Department of Chemistry, University of Patras, University Campus Rio, 26504 Patras, Greece; dafnigraikioti@upnet.gr; 2 Department of Surgical Sciences and Integrated Diagnostics (DISC), University of Genoa, Viale Benedetto XV 6, 16132 Genova, Italy; amschito@unige.it; 3 Department of Pharmacy, University of Genoa, Viale Cembrano, 16148 Genoa, Italy

**Keywords:** multidrug-resistant (MDR) bacteria (superbugs), natural triterpenoids, triphenyl phosphonium (TPP) group, minimum inhibitory concentration (MIC), strong antibacterial effects

## Abstract

**Background**: To meet the urgent need for novel antibacterial agents that are active also against worrying superbugs, natural pentacyclic triterpenoids, including totally inactive betulin (BET) and betulinic acid (BA), as well as ursolic acid (UA), active on Gram-positive bacteria, have been chemically modified, achieving compounds **1**–**7**. **Methods**: Triterpenoid derivatives **1**–**7** and all synthetic intermediates were characterized by chemometric-assisted FTIR and NMR spectroscopy, as well as by other analytical techniques, which confirmed their structure and high purity. Minimum inhibitory concentration values (MICs) of **1**–**7**, BET, BA and UA were determined by the broth dilution method, using a selection of Gram-positive and Gram-negative clinically isolated superbugs. **Results**: Performed experiments evidenced that compounds **4**–**7** had potent antibacterial effects against Gram-positive methicillin-resistant *Staphylococcus aureus* and *S. epidermidis* (MRSA and MRSE), as well as against vancomycin-resistant *Enterococcus faecalis* and *E. faecium* (VRE). The antibacterial effects of **4**–**7** were due to the insertion of a triphenyl phosphonium (TPP) group and were higher than those reported so far for other BET, BA and UA derivatives, especially considering the complex pattern of resistance of the isolates used here and their clinical source. **Conclusions**: For the first time, by inserting TPP, a real activity (MICs 2–16 µg/mL) was conferred to inactive BET and BA (MICs > 1024 and 256 µg/mL). Moreover, the antibacterial effects of UA were improved 16- and 32-fold against MRSE and MRSA (MICs = 2 vs. 32 and 64 μg/mL). **Future Perspectives**: Based on these very promising microbiologic results, new experiments are currently underway with the best-performing compounds **5** and **7** (MICs = 2 μg/mL) on an enlarged number of Gram-positive isolates, to confirm their MICs. Moreover, investigations about their possible antibiofilm activity, time-killing curves and cytotoxicity on eukaryotic cells will be carried out to define their pharmacological behavior and clinical potential.

## 1. Introduction

A superbug is usually defined as a multidrug-resistant (MDR) microorganism that has become resistant to multiple antibiotics [1]. The number of different antibiotics to which it can be resistant establishes the extent to which it can be considered a superbug [1]. The superbug of all superbugs is a bacterium that has developed resistance to all available antibiotics, causing increased morbidity, mortality rate and economic loss [1]. Major superbugs develop in hospital settings and include methicillin-resistant *Staphylococcus aureus* (MRSA) and MDR Gram-negative bacilli, including *Pseudomonas aeruginosa*, carbapenem-resistant *Enterobacteriaceae*, extended-spectrum-β-lactamase (ESBL)-producing *Enterobacteriaceae*, vancomycin-resistant enterococci (VRE), *E. coli* Hx30 and others [1].

Major risk factors for the development of antibiotic-resistant superbugs include overprescription and misuse of antibiotics, their use without medical indication, poor drug quality, genetic mutation among microorganisms, patients not completing the entire antibiotic dosage or not strictly following the correct antibiotic regimen, long-term hospitalization, prophylactic antibiotic therapy and poor hygiene and sanitation [2].

According to a report by Mancuso et al. in *Pathogens* in 2021, antibiotic-resistant bacteria caused 700,000 deaths worldwide each year [3]. The World Health Organization (WHO) predicted that without new and better treatments, this number could rise to 10 million by 2050, highlighting a health concern of paramount importance [3].

Recently, the increasing development of antibiotic resistance has led some Gram-negative bacteria to become tolerant also to last-resort antibiotics, used in the treatment of non-fermentative species in critically ill patients [4]. In this regard, drug resistance has been reported against colistin and the recently approved cefiderocol (FDC) [4]. Particularly, colistin is an older polycationic antibiotic, traditionally employed in the management of infections sustained by *Enterobacteriaceae* that have developed resistance to practically all other antibiotics [5]. Since 2016, many Gram-negative bacteria have been shown to possess genes that also confer resistance to colistin, thus further reducing the available weapons to treat the infections they cause [5]. Furthermore, FDC is a strategically catechol-substituted siderophore cephalosporin, capable of killing Gram-negative bacteria [6]. Nevertheless, cases of in vivo-emerging FDC resistance are increasingly being reported [7]. Moreover, some Gram-negative superbugs show a complex pattern of resistance, including cross-resistance to carbapenems, colistin, the combination ceftazidime–avibactam (Zavicefta) and FDC [8].

However, infections sustained by Gram-positive superbugs are also of serious concern. Although there are many more drugs available to combat them, many more drug-resistant infections from Gram-positive bacteria exist compared with those from Gram-negative strains [9].

Dr. Mark Blaskovich, a senior research chemist at the Institute for Molecular Bioscience (IMB) at the University of Queensland in Australia, described MRSA as the “poster child” of Gram-positive superbugs [10].

Blaskovich points to a report from the Centers for Disease Control (CDC) in the US from 2013, which counted the number of infections and the number of deaths from different types of drug-resistant bacteria [10].

The number of infections from drug-resistant Gram-positive MRSA or strep pneumonia was over a million, versus about 30,000 from Gram-negative bacterial infections.

Also, drug-resistant Gram-positive bacteria were by far the biggest killers in the report, too. The number of deaths from resistant bacteria was about fivefold higher for Gram-positive compared to Gram-negative infections [10]. Particularly, VRE enterococcal species are MDR bacteria that, in addition to resistance to vancomycin, have already developed a variety of mechanisms of resistance to several other antibiotics, like aminoglycosides, β-lactams, tetracyclines and quinolones. Additionally, they can produce β-lactamases and have decreased cellular permeability, thus being the cause of severe hospital-acquired infections [11]. VREs are reported as the main cause of central line-associated bloodstream infections (CLABSIs), catheter-associated urinary tract infections (CAUTIs), ventilator-associated pneumonia (VAP) and surgical site infections (SSIs). VREs are categorized as a “serious threat” by the Centers for Disease Control (CDC) and Prevention [12]. Moreover, staphylococci, especially MRSA, are the leading cause of nosocomial infections, antibiotic-resistant diseases, central line-associated bacteraemia and hospital-associated endocarditis in the USA [13,14]. Notably, both MRSA and VRE are reported as the most common causes of community-acquired endocarditis in North America [15]. Very common in hospitals, prisons and nursing homes, where immunocompromised patients and people with open wounds and/or invasive devices such as catheters are at greater risk of hospital-acquired infections, MRSA represents a global health threat and a clear ‘One Health’ problem. Moreover, MRSA can spread between and impact the environment, animals and several human sectors [16]. Concerning the current available armamentarium to counteract these superbugs, vancomycin is successful only in about 49% of cases of MRSA infection [8]. Its use is complicated by its inconvenient route of administration [17], and, since the late 1990s, the emergence of several vancomycin- and teicoplanin-resistant strains of MRSA has been reported [18]. Oxazolidinones such as linezolid (LNZ), available from the 1990s, were initially beneficial in limiting the widespread infections by Gram-positive superbugs, representing one of the last-line therapeutic options for serious infections caused by VRE, MRSE and MDR *Enterococcus* and *Staphylococcus* species, but cases of bacteria tolerant to LNZ have been reported since 2001 [19]. According to data from recent large-scale studies, isolates of the MDR *S. epidermidis* genus have demonstrated resistance to LNZ [8]. Additionally, epidemiologic data have shown a 2.5-fold increase in the prevalence of clinical LNZ-resistant enterococci (LRE) over the past decade, with a global detection rate of 1.1% for LNZ-resistant *E. faecium* (LREfm) and 2.2% for LNZ-resistant *E. faecalis* (LREfs) [20]. Most reported cases have originated from China, followed by South Korea and the United States [20].

For surgical site infections (SSIs) by MRSA [21] and for MRSA colonization in nonsurgical wounds, such as traumatic wounds, burns and chronic ulcers (i.e., diabetic ulcer, pressure ulcer, arterial insufficiency ulcer and venous ulcer), no conclusive evidence has been found regarding the best antibiotic regimen to be used [22]. Although infection control and antimicrobial stewardship are important tools for combating the development and spread of lethal infections, their uncontrolled development will lead to a point where conducting surgeries such as C-sections and transplants will be too dangerous, due to the risk of superbug infection, which would have huge implications for the health of people and economic safety around the world. Collectively, we are living in an era of missing epidemiologic evidence and of a plethora of uncertainties, due to the interindividual responses of patients to existing antibiotics, where the decreasing efficacy of available drugs urgently requires the development of new curative options against difficult-to-treat superbugs.

**Figure 1:**
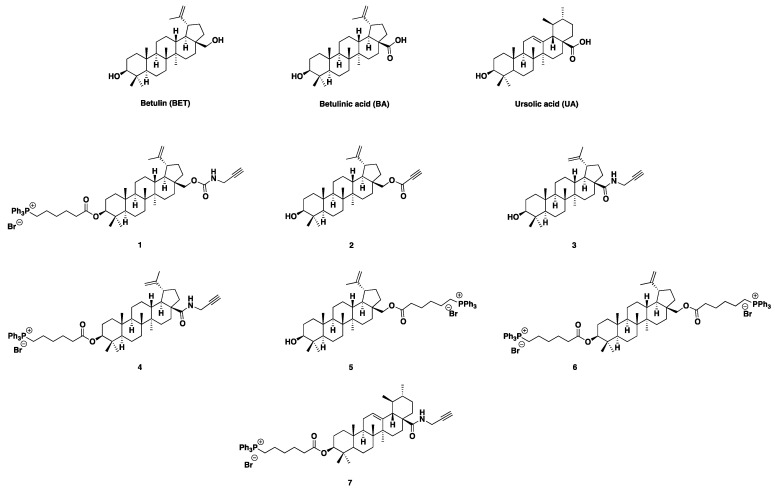
Compounds synthesized in this work.

To meet these needs, in this study, seven betulin (BET), betulinic acid (BA) and ursolic acid (UA) derivatives (**1**–**7**) have been synthesized and fully characterized. Compounds **1**, **4**, **5** and **7** contained 1 triphenyl phosphonium (TPP) group. In the case of compound **5**, the TPP group was inserted as an ester in C-28 of pristine BET, while in the other cases, it was inserted as an ester in C-3 of BET, BA and UA, since position C-28 was functionalized with propargyl amine carbamate (**1**) and propargyl amide (**4**, **7**) residues. Compound **6** contained 2 TPP groups as esters in both C-3 and C-28. Compounds **2** and **3** did not bear the TPP groups, but only a propiolic ester (**2**) or a propargyl amide (**3**) residue inserted in C-28 of BET or BA, respectively. Compounds **5** and **6** did not contain the propine derivatives, but only the TPP group (Figure 1).

Compounds **1**–**7** and all synthetic intermediates were characterized by chemometric-assisted FTIR and NMR spectroscopy, as well as by other analytical techniques. The minimum inhibitory concentration values (MICs) of **1**–**7**, BET, BA and UA were assessed by the broth dilution method according to EUCAST [23], using a selection of Gram-positive and Gram-negative clinically isolated superbugs.

## 2. Materials and Methods

### 2.1. Chemicals and Instruments

All solvents (Acros Organics, Geel, Belgium) were dried and purified according to standard procedures prior to use. When required, reactions were performed under an inert atmosphere (Ar) in pre-flamed glassware. Anhydrous Na_2_SO_4_ was used for drying solutions, and the solvents were then routinely removed at ca. 40 °C under reduced pressure (ca 10–20 mmHg), using a rotary evaporator. All reagents employed in the present work were commercially available and used without further purification. Flash column chromatography (FCC) was performed on silica gel (70–230 and 230–400 mesh, Merck, Darmstadt, Germany) and analytical thin layer chromatography (TLC) on silica gel 60-F_254_ precoated aluminium foils (0.2 mm film, Merck, Germany). Spots on the TLC plates were visualized with UV light at 254 nm and using ninhydrin, *p*-anisaldehyde or charring solution. Attenuated total reflectance (ATR) Fourier transform infrared (FTIR) analyses were carried out using a Spectrum Two FT-IR Spectrometer (PerkinElmer, Inc., Waltham, MA, USA), as previously reported [24]. ^1^H, ^13^C and ^31^PNMR spectra were recorded in CDCl_3_ at 600, 151 and 243 MHz on a Bruker AVANCEIII HD spectrometer (Billerica, MA, USA). Fully chemical shifts (*δ*) are indicated in parts per million downfield from TMS, and coupling constants (*J*) are reported in Hz. Gas chromatography–mass spectrometry (GC-MS) spectra were performed on a Varian Saturn 2000 ion trap GC-MS instrument (Artisan Technology Group ^®^, Champaign, IL, USA) equipped with a DB-5MS column (30 m, i.d. 0.25 mm) (Agilent, Santa Clara, CA, USA). ESI mass spectra were recorded at 30 V on an amaZon SL ion trap mass spectrometer, Bruker Daltonics (Billerica, MA, USA), using MeOH as solvent. Elemental analyses were carried out using an Elemental Analyzer (Fison Instruments Ltd., Glasgow, UK).

### 2.2. Synthesis of Triterpenoid Derivatives

With the aim of finding novel compounds effectively active against difficult-to-treat clinically isolated superbugs, seven triterpenoid derivatives (**1**–**7**) were synthesized via chemical modifications of betulin (BET), betulinic acid (BA) and ursolic acid (UA).

#### 2.2.1. Synthesis of Betulin (BET) Derivatives

**Scheme 1:**
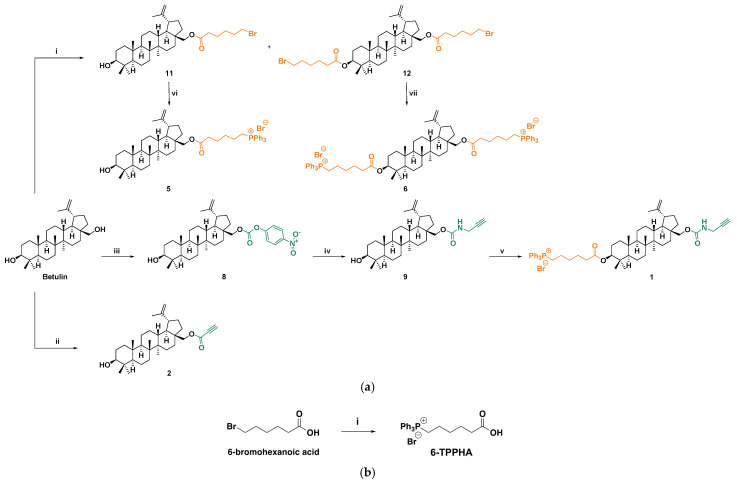
(**a**) Synthesis of the BET derivatives **1**, **2**, **5** and **6** and intermediates **8**, **9**, **11** and **12**. Legend to scheme: (**i**) 6-bromohexanoic acid, dichloromethane (DCM)-tetra-hydro-furane (THF), 4-di-methylamino pyridine (DMAP), dicyclohexyl carbodiimide (DCC), 0 °C to r. t., 48 h, 88% (**11**, 57% and **12**, 31%); (**ii**) propiolic acid, DCC, DMAP, DCM, −10 °C to r. t., 24 h, 38%; (**iii**) *p*-nitro-phenyl-chloroformate, THF, pyridine, 0 °C to r. t., 24 h, 83%; (**iv**) propargylamine, triethylamine (Et_3_N); THF, 0 °C to r. t., 3 h, 65%; (**v**) 6-triphenyl phosphonium hexanoic acid **10**, DCM, DCC, DMAP, 0 °C, to r. t., 90 min, 54%; (**vi**, **vii**) triphenylphosphine (Ph_3_P), toluene (PhMe), reflux, 48 h, 30–36%. (**b**) Synthesis of 6-TPPHA (**10**). Legend to scheme: (i) Ph_3_P, PhMe, 85 °C overnight, 53%. Chemical modifications leading to the introduction of triple-bond-containing moieties are in green color, while those leading to the insertion of the triphenyl phosphonium hexanoate group are in orange color.

BET derivatives were synthesized according to Scheme 1a via intermediates **8**, **9**, **11** and **12**. Reagents used have been described in the legend available in Scheme 1a’s caption.

*(3β)-Lup-20(29)-ene-3,28-diol*, *28-(4-nitrophenyl carbonate)* **8**. To a stirring solution of BET (200 mg, 0.45 mmol) in dry THF (10 mL), under an Ar atmosphere, pyridine (36 μL, 0.45 mmol) was added, and the mixture was cooled to 0 °C. *p*-nitro-phenyl-chloroformate (94.3 mg, 0.47 mmol) was inserted in 3 portions, and the reaction mixture was stirred for 24 h at room temperature (r. t.). Following, the mixture was concentrated under reduced pressure to dryness. The obtained solid residue was diluted in DCM and washed sequentially with 5% aqueous ice-cold citric acid, water and brine, dried over anhydrous Na_2_SO_4_, filtered and concentrated to dryness. The desired product was afforded as a white foam (227 mg, 0.37 mmol, 83% yield) after flash column chromatography (FCC) purification using PhMe:AcOEt 95:5 as eluent. R_f_ (PhMe:AcOEt 95:5) = 0.14.

^1^H NMR (600 MHz, CDCl_3_) *δ* 8.29 (s, 1 H), 8.28 (s, 1 H), 7.41 (s, 1 H), 7.39 (s, 1 H), 4.71 (br s, 1 H), 4.61 (t, *J* = 1.7 Hz, 1 H), 4.51 (dd, *J* = 10.8 Hz, 2.0 Hz, 1 H), 4.08 (d, *J* = 10.7 Hz, 1 H), 3.19 (dd, *J* = 11.4, 4.8 Hz, 1 H), 2.45 (td, *J* = 10.8, 5.4 Hz, 1 H), 2.03 (dtd, *J* = 14.2, 10.5, 4.9 Hz, 1 H), 1.95–1.84 (m, 2 H), 1.75 (td, *J* = 13.9, 4.7 Hz, 1 H), 1.70 (s, 3 H), 1.68–1.08 (m, 19 H), 1.05 (s, 3 H), 1.00 (s, 3 H), 0.97 (s, 3 H), 0.91 (td, *J* = 12.5, 3.7 Hz, 1 H), 0.83 (s, 3 H), 0.76 (s, 3 H), 0.69 (d, *J* = 11.2 Hz, 1 H); ^13^C NMR (151 MHz, CDCl_3_) *δ* 155.6, 153.0, 149.7, 145.3, 125.3, 121.8, 110.1, 78.9, 68.3, 55.3, 50.3, 48.8, 47.7, 46.7, 42.7, 40.9, 38.9, 38.7, 37.7, 37.1, 34.4, 34.2, 29.6, 29.5, 28.0, 27.4, 27.0, 25.2, 20.7, 18.3, 16.1, 16.0, 15.3, 14.8.

*(3β)-Lup-20(29)-ene-3,28-diol*, *28-(N-propargyl carbamate)* **9**. To an ice-cooled solution of propargylamine (10 μL, 8.6 mg, 0.156 mmol, 1.9 equiv.) in THF (0.42 mL), **8** (50 mg, 0.0823 mmol) and Et_3_N (0.030 mL, 0.22 mmol, 2.6 equiv.) were added portion-wise, and the resulting mixture was left under stirring at 0 °C to r.t. Upon consumption of the starting material, the reaction mixture was concentrated to dryness. Then, the residue was diluted in EtOAc and washed twice with NaHCO_3 (aq)_ 5%, water, K_2_CO_3 (aq)_ 10%, water and NaCl _(sat.)_. The organic layer was dried over anhydrous Na_2_SO_4_, filtered, and concentrated to dryness and the pure product was afforded as a colorless oil (28 mg, 65% yield) after FCC purification using PhMe:EtOAc 97:3 as eluent. R*f* (PhMe:EtOAc 97:3) = 0.12.

^1^H NMR (CDCl_3_, 600 MHz) *δ* 4.95 (br s, 1 H), 4.67 (d, *J* = 2.3 Hz, 1 H), 4.57 (d, *J* = 1.9 Hz, 1 H), 4.27 (d, *J* = 10.8 Hz, 1 H), 3.98 (br s, 2 H), 3.86 (d, *J* = 10.8 Hz, 1 H), 3.18 (dd, *J* = 11.5, 4.7 Hz, 1 H), 2.43 (td, *J* = 11.2, 5.7 Hz, 1 H), 2.24 (t, *J* = 2.4 Hz, 1 H), 1.97 (dq, *J* = 14.0, 10.2 Hz, 1 H), 1.81 (d, *J* = 13.6 Hz, 1 H), 1.76 (m, 1 H), 1.73 (m, 1 H), 1.67 (s, 3 H), 1.64–1.49 (m, 8 H), 1.38 (m, 5 H), 1.25 (dd, *J* = 12.6, 2.3 Hz, 2 H), 1.19 (qd, *J* = 12.5, 4.1 Hz, 2 H), 1.04 (m, 1 H), 1.02 (s, 3 H), 0.96 (s, 6 H), 0.88 (td, *J* = 13.4, 4.4 Hz, 2 H), 0.81 (s, 3 H), 0.75 (s, 3 H), 0.66 (d, *J* = 9.5 Hz, 1 H); ^13^C NMR (CDCl_3_, 151 MHz) *δ* 157.0, 150.5, 129.4, 128.6, 126.5, 116.0, 110.2, 80.1, 79.3, 71.9, 64.0, 55.6, 50.7, 49.1, 48.0, 46.9, 43.0, 41.2, 39.2, 37.9, 37.5, 34.5, 31.2, 29.9, 28.3, 27.7, 27.4, 25.5, 21.1, 19.5, 18.6, 16.4, 15.7, 15.1.

*6-Tri Phenyl Phosphonium Hexanoic Acid* **10**. To a solution of Ph_3_P (806 mg, 3.07 mmol, 1.2 equiv.) in PhMe (4.27 mL, 0.6 M), 6-bromohexanoic acid (500 mg, 2.56 mmol) was added and the resulting mixture was heated, under stirring, at 85 °C overnight. Following, the resulting suspension was left to cool down at r. t., then cooled to 0 °C and filtered under vacuum. The solid residue was washed twice with diethyl ether (Et_2_O) and dried. The pure product was afforded without further purification as a white solid (621 mg, 53% yield).

*(3β)-Lup-20(29)-Ene-3,28-Diol-3-(6-Triphenyl Phosphonium Hexanoate)-28-(N-propargyl carbamate)* **1**. To a solution of **9** (28 mg, 0.053 mmol) in DCM (0.180 mL), 6-triphenyl phosphonium hexanoic acid **10** (6-TPPHA), synthesized as described in Scheme 1a (24 mg, 0.052 mmol, 0.99 equiv.), and DMAP (0.64 mg, 0.0053 mmol, 0.1 equiv.) were added, and the mixture was cooled to 0 °C. Then, DCC (12 mg, 0.058 mmol, 1.1 equiv.) was added portion-wise, and the resulting mixture was stirred at 0 °C to r. t. Upon completion of the reaction, the mixture was filtered under vacuum, and the solid residue was washed twice with EtOAc. The filtrate was concentrated to dryness, and the oily residue was subjected to FCC purification using EtOAc:methanol (MeOH):acetic acid (AcOH) 9:1:0.1 as eluent, to afford the pure product as a colorless oil (28 mg, 54% yield). R*f* (AcOEt:MeOH:AcOH 9:1:0.1) = 0.13.

ATR-FTIR (ṽ, cm^−1^): 3060 (=C-H), 2941, 2869 (C-H), 1715 (NHC=OO), 1438 (C-H), 1246, 1112 (C-O), 723, 689 (C-P). ^1^H NMR (CDCl_3_, 600 MHz) *δ* 7.84 (ddd, *J* = 12.6, 8.3, 1.3 Hz, 6 H), 7.77 (td, *J* = 7.4, 1.7 Hz, 3 H), 7.68 (td, *J* = 7.8, 3.4 Hz, 6 H), 4.88 (br s, 1 H), 4.66 (d, *J* = 2.3 Hz, 1 H), 4.56 (t, *J* = 2.0 Hz, 1 H), 4.39 (m, 1 H), 4.27 (d, *J* = 10.8 Hz, 1 H), 3.97 (m, 2 H), 3.84 (ddt, *J* = 21.0, 15.6, 8.4 Hz, 3 H), 2.42 (td, *J* = 10.8, 5.4 Hz, 1 H), 2.24 (m, 3 H), 1.96 (dq, *J* = 14.0, 10.4 Hz, 1 H), 1.80 (m, 1 H), 1.71 (ddd, *J* = 13.7, 9.3, 6.2 Hz, 4 H), 1.66 (s, 3 H), 1.65–1.51 (m, 9 H), 1.46 (m, 1 H), 1.37 (m, 5 H), 1.25 (m, 2 H), 1.18 (qd, *J* = 12.6, 4.2 Hz, 2 H), 1.01 (s, 6 H), 0.94 (s, 3 H), 0.88 (m, 1 H), 0.81 (s, 3 H), 0.77 (s, 3 H), 0.76 (s, 3 H), 0.73 (d, *J* = 9.6 Hz, 1 H); ^13^C NMR (CDCl_3_, 151 MHz) *δ* 173.6, 150.0, 135.2, 135.2, 134.0, 133.9, 130.7, 130.7, 118.9, 118.3, 110.0, 81.0, 80.0, 71.8, 55.6, 50.5, 49.0, 47.9, 46.8, 42.9, 41.1, 38.6, 38.0, 37.7, 37.3, 34.7, 34.3, 34.3, 30.0, 29.9, 28.2, 27.3, 25.4, 24.7, 23.9, 23.0, 22.7, 22.7, 22.7, 21.0, 19.3, 18.4, 16.8, 16.4, 16.2, 14.9; ^31^P NMR (CDCl_3_, 243 MHz) *δ* 24.38. GC-MS: m/e: 963.48 (100.0%), 961.48 (84.1%), 962.48 (55.4%), 964.48 (54.4%), 965.48 (18.3%), 964.49 (3.8%), 966.49 (3.8%). ESI-MS (30 eV): *m*/*z* 882.75 [(M-Br^−^)^+^], 962.54 [M + H^+^]; Anal. Calcd. for C_58_H_77_BrO_4_P (Mol. Wt.: 963.11): C, 72.33; H, 8.06; Br, 8.30; N, 1.45; O, 6.64; P, 3.22. Found: C, 72.13; H, 8.38; Br, 8.38; O, 6.52; P, 3.43.

*(3β)-Lup-20(29)-Ene-3,28-Diol, 28-(2-Propynoate)* **2**. To an ice-cooled (−10 °C) suspension of BET (40 mg, 0.09 mmol) and propiolic acid (10 μL, 0.161 mmol, 1.79 equiv.) in DCM (0.45 mL), a solution of DCC (21 mg, 0.102 mmol, 1.13 eq.) and DMAP (1 mg, 0.008 mmol, 0.09 eq.) in DCM (0.2 mL) was added portion-wise, and the resulting brown mixture was stirred at −10 °C to r. t. Upon completion of the reaction, the mixture was diluted with cold EtOAc and filtered under vacuum. The filtrate was concentrated to dryness, and the oily residue was subjected to FCC purification using PhMe:EtOAc 97:3 as eluent, to afford the pure product as a colorless oil (17 mg, 38% yield). R*f* (PhMe:EtOAc 97:3) = 0.18.

ATR-FTIR (ṽ, cm^−1^): 3284 (OH), 3074 (=C-H), 2941, 2867 (C-H), 2120 (C_sp_-H), 1716 (OC=O), 1455 (C-H), 1221 (C-O). ^1^H NMR (CDCl_3_, 600 MHz) *δ* 4.68 (d, *J* = 2.2 Hz, 1 H), 4.58 (t, *J* = 1.8 Hz, 1 H), 4.37 (dd, *J* = 11.1, 2.0 Hz, 1 H), 3.98 (d, *J* = 11.0 Hz, 1 H), 3.17 (dd, *J* = 11.5, 4.7 Hz, 1 H), 2.89 (s, 1 H), 2.42 (td, *J* = 10.7, 5.8 Hz, 1 H), 1.97 (dtd, *J* = 14.1, 10.6, 8.4 Hz, 1 H), 1.88 (ddd, *J* = 13.6, 4.6, 2.6 Hz, 1 H), 1.81 (dd, *J* = 12.7, 8.3 Hz, 1 H), 1.68 (s, 4 H), 1.66–1.58 (m, 5 H), 1.57–1.48 (m, 3 H), 1.44–1.37 (m, 6 H), 1.32–1.24 (m, 2 H), 1.20 (qd, *J* = 12.8, 4.3 Hz, 1 H), 1.13 (ddd, *J* = 12.6, 10.4, 2.1 Hz, 1 H), 1.07 (ddd, *J* = 14.1, 4.4, 2.7 Hz, 1 H), 1.03 (s, 3 H), 0.97 (s, 3 H), 0.96 (s, 3 H), 0.90 (td, *J* = 12.9, 4.2 Hz, 1 H), 0.81 (s, 3 H), 0.75 (s, 3 H), 0.67 (m, 1 H); ^13^C NMR (CDCl_3_, 151 MHz) *δ* 153.5, 150.1, 110.2, 79.2, 75.0, 74.9, 65.1, 55.5, 50.6, 49.0, 47.9, 46.6, 42.9, 41.1, 39.1, 38.9, 37.9, 37.4, 34.7, 34.4, 29.8, 29.7, 28.2, 27.6, 27.2, 25.4, 21.0, 19.3, 18.5, 16.3, 16.2, 15.6, 15.0. GC-MS: m/e: 494.38 (100.0%), 495.38 (37.6%), 496.38 (7.2%). ESI-MS (30 eV): *m*/*z* 493.43 [M-H^−^]; Anal. Calcd. for C_33_H_50_O_3_ (Mol. Wt.: 494.75): C, 80.11; H, 10.19; O, 9.70. Found: C, 79.93; H, 10.02; O, 9.99.

Mixture *(3β)-Lup-20(29)-Ene-3,28-Diol, 28-(6-Bromo-Hexanoate)* **11** + *(3β)-Lup-20(29)-Ene-3,28-Diol, 3,28-di-(6-Bromo-Hexanoate)* **12**. To an ice-cooled suspension of BET (100 mg, 0.226 mmol) and 6-bromohexanoic acid (133 mg, 0.68 mmol, 3.0 equiv.) in a mixture of DCM (1.15 mL) and THF (0.5 mL), DMAP (3 mg, 0.02 mmol, 0.1 equiv.) and DCC (119 mg, 0.57 mmol, 2.55 eq.) were added, and the resulting mixture was stirred at 0 °C to r. t. Upon completion of the reaction, the mixture was diluted with cold EtOAc and filtered under vacuum. The filtrate was concentrated to dryness, and the oily residue was subjected to FCC purification using gradient PhMe:EtOAc 97:3 to 95:5 as eluent, to afford the pure product **12** as a colorless oil (55 mg, 31% yield), R*f* (PhMe:EtOAc 97:3) = 0.70, and the pure product **11** as a colorless oil (80 mg, 57% yield), R*f* (PhMe:EtOAc 97:3) = 0.15.

*(3β)-Lup-20(29)-Ene-3,28-Diol, 28-(6-Bromo-Hexanoate)* **11**. ^1^H NMR (CDCl_3_, 600 MHz) δ 4.68 (d, *J* = 2.2 Hz, 1 H), 4.58 (m, 1 H), 4.26 (dd, *J* = 11.1, 2.0 Hz, 1 H), 3.84 (dd, *J* = 11.1, 1.4 Hz, 1 H), 3.40 (t, *J* = 6.8 Hz, 2 H), 3.18 (d, *J* = 11.3 Hz, 1 H), 2.44 (td, *J* = 11.2, 5.8 Hz, 1 H), 2.34 (m, 2 H), 1.96 (m, 1 H), 1.88 (m, 2 H), 1.82 (ddd, *J* = 13.4, 4.5, 2.5 Hz, 1 H), 1.75 (ddd, *J* = 12.4, 8.5, 1.3 Hz, 1 H), 1.67 (s, 6 H), 1.65–1.57 (m, 5 H), 1.56–1.43 (m, 5 H), 1.42–1.30 (m, 6 H), 1.26 (qt, *J* = 7.3, 4.0 Hz, 2 H), 1.20 (td, *J* = 12.9, 4.4 Hz, 1 H), 1.13–1.04 (m, 2 H), 1.02 (s, 3 H), 0.97 (s, 3 H), 0.96 (s, 3 H), 0.89 (td, *J* = 13.4, 4.5 Hz, 1 H), 0.81 (s, 3 H), 0.75 (s, 3 H), 0.67 (dd, *J* = 9.1, 2.2 Hz, 1 H); ^13^C NMR (CDCl_3_, 151 MHz) δ 174.1, 150.4, 110.1, 79.2, 62.9, 55.5, 50.6, 49.0, 47.9, 46.6, 42.9, 41.1, 39.1, 38.9, 37.8, 37.4, 34.8, 34.4, 34.4, 33.7, 32.6, 30.0, 28.2, 27.9, 27.6, 27.8, 27.3, 24.4, 24.2, 21.6, 21.0, 18.5, 16.3, 16.3, 15.6, 15.0.

*(3β)-Lup-20(29)-Ene-3,28-Diol, 3,28-di-(6-Bromo-Hexanoate)* **12**. ^1^H NMR (CDCl_3_, 600 MHz) δ 4.68 (d, *J* = 2.2 Hz, 1 H), 4.59 (t, *J* = 1.9 Hz, 1 H), 4.47 (dd, *J* = 10.6, 5.8 Hz, 1 H), 4.27 (dd, *J* = 11.1, 1.9 Hz, 1 H), 3.84 (d, *J* = 11.0 Hz, 1 H), 3.40 (td, *J* = 6.8, 2.3 Hz, 4 H), 2.44 (td, *J* = 11.2, 5.8 Hz, 1 H), 2.33 (m, 4 H), 1.96 (dtd, *J* = 14.1, 10.6, 8.5 Hz, 1 H), 1.88 (pd, *J* = 6.9, 2.2 Hz, 4 H), 1.82 (ddd, *J* = 13.3, 4.5, 2.5 Hz, 1 H), 1.76 (m, 1 H), 1.68 (s, 5 H), 1.65 (m, 6 H), 1.59 (m, 4 H), 1.49 (tdd, *J* = 9.9, 6.6, 2.2 Hz, 5 H), 1.39 (m, 5 H), 1.28 (m, 2 H), 1.21 (m, 1 H), 1.07 (m, 3 H), 1.03 (s, 3 H), 0.97 (s, 3 H), 0.84 (s, 3 H), 0.83 (s, 6 H), 0.79 (m, 1 H); ^13^C NMR (CDCl_3_, 151 MHz) δ 174.1, 173.5, 150.3, 110.1, 81.0, 62.9, 55.6, 50.5, 49.0, 47.9, 46.6, 42.9, 41.1, 38.6, 38.1, 37.8, 37.3, 34.8, 34.4, 34.3, 33.7, 32.6, 32.6, 30.0, 29.8, 28.2, 28.1, 27.9, 27.3, 25.4, 24.5, 24.4, 24.0, 21.0, 19.3, 18.4, 16.8, 16.4, 16.3, 15.0.

*(3β)-Lup-20(29)-Ene-3,28-Diol, 28-(6-Triphenyl Phosphonium-Hexanoate)* **5**. A solution of **11** (30 mg, 0.048 mmol) and Ph_3_P (15 mg, 0.057 mmol, 1.2 equiv.) in PhMe (100 μL) was heated under reflux. Upon consumption of the starting material, the mixture was subjected to FCC purification using chloroform (CHCl_3_):MeOH 92:8 as eluent, to afford the pure product as a colorless oil (15 mg, 36% yield). R*f* (CHCl_3_:MeOH 92:8) = 0.08.

ATR-FTIR (ṽ, cm^−1^): 3362 (OH), 3057 (=C-H), 2958, 2867 (C-H), 1726 (C=OO), 1438 (C-H), 1112 (C-O), 723, 690 (C-P). ^1^H NMR (CDCl_3_, 600 MHz) *δ* 7.84 (dd, *J* = 12.7, 7.8, Hz, 6 H), 7.78 (td, *J* = 7.5, 1.8 Hz, 3 H), 7.69 (td, *J* = 7.8, 3.4 Hz, 6 H), 4.66 (d, *J* = 2.4 Hz, 1 H), 4.56 (s, 1 H), 4.21 (dd, *J* = 11.1, 1.9 Hz, 1 H), 3.87 (td, *J* = 12.5, 11.9, 6.1 Hz, 2 H), 3.78 (d, *J* = 11.0 Hz, 1 H), 3.17 (dd, *J* = 11.6, 4.7 Hz, 1 H), 2.40 (td, *J* = 11.2, 6.0 Hz, 1 H), 2.29 (t, *J* = 7.4 Hz, 2 H), 1.90 (dtd, *J* = 14.1, 10.7, 8.6 Hz, 2 H), 1.77 (ddd, *J* = 13.9, 4.6, 2.6 Hz, 1 H), 1.70 (m, 4 H), 1.66 (s, 3 H), 1.62 (m, 6 H), 1.54 (m, 5 H), 1.38 (m, 5 H), 1.21 (m, 4 H), 1.03 (m, 2 H), 1.00 (s, 3 H), 0.95 (s, 6 H), 0.88 (tt, *J* = 14.0, 4.8 Hz, 1 H), 0.80 (s, 3 H), 0.74 (s, 3 H), 0.66 (d, *J* = 9.7 Hz, 1 H); ^13^C NMR (CDCl_3_, 151 MHz) *δ* 174.0, 150.2, 135.0, 135.0, 133.8, 133.7, 133.7, 133.6, 130.5, 130.5, 130.4, 129.0, 128.2, 125.3, 118.7, 118.1, 110.1, 78.9, 62.5, 55.3, 50.3, 48.8, 47.7, 46.4, 42.7, 40.9, 38.8, 38.7, 37.6, 37.1, 34.5, 34.2, 33.7, 29.8, 29.7, 29.6, 28.0, 27.4, 27.0, 25.2, 24.5, 22.5, 22.5, 21.4, 20.8, 19.1, 18.3, 16.1, 16.0, 15.4, 14.7; ^31^P NMR (CDCl_3_, 243 MHz) *δ* 24.41. GC-MS: m/e: 880.46 (100.0%), 882.45 (97.3%), 881.46 (61.3%), 883.46 (60.0%), 884.46 (18.5%), 882.46 (18.4%), 885.46 (3.7%), 883.47 (3.6%). ESI-MS (30 eV): *m*/*z* 801.67 [(M-Br^−^)^+^]; Anal. Calcd. for C_54_H_74_BrO_3_P (Mol. Wt.: 882.04): C, 73.53; H, 8.46; Br, 9.06; O, 5.44; P, 3.51. Found: C, 73.13; H, 8.50; Br, 9.38; O, 5.42; P, 3.73.

*(3β)-Lup-20(29)-Ene-3,28-Diol, 3-28-di-(6-Triphenyl Phosphonium-Hexanoate)* **6**. A solution of **12** (37 mg, 0.046 mmol) and Ph_3_P (30 mg, 0.112 mmol, 2.4 equiv.) in PhMe (100 μL) was heated under reflux. Upon consumption of the starting material, the mixture was subjected to FCC purification using CHCl_3_:MeOH 92:8 as eluent, to afford the pure product as a colorless oil (18 mg, 30% yield). R*f* (CHCl_3_:MeOH) 92:8 = 0.08.

ATR-FTIR (ṽ, cm^−1^): 3054 (=C-H), 2941, 2867 (C-H), 1721 (C=OO), 1436 (C-H), 1112 (C-O), 745, 722, 690 (C-P). ^1^H NMR (CDCl_3_, 600 MHz) *δ* 7.83 (m, 12 H), 7.78 (t, *J* = 7.6 Hz, 6 H), 7.69 (m, 12 H), 4.65 (d, *J* = 2.4 Hz, 1 H), 4.55 (s, 1 H), 4.38 (dd, *J* = 9.0, 7.4 Hz, 1 H), 4.20 (dd, *J* = 11.1, 1.9 Hz, 1 H), 3.83 (tt, *J* = 12.3, 7.6 Hz, 4 H), 3.77 (d, *J* = 11.0 Hz, 1 H), 2.39 (td, *J* = 11.1, 5.7 Hz, 1 H), 2.28 (t, *J* = 7.4 Hz, 2 H), 2.24 (td, *J* = 7.4, 1.9 Hz, 2 H), 1.89 (m, 4 H), 1.72 (m, 6 H), 1.67 (m, 1 H), 1.65 (s, 3 H), 1.61 (m, 10 H), 1.53 (m, 3 H), 1.45 (m, 1 H), 1.35 (m, 5 H), 1.25 (dd, *J* = 12.6, 2.6 Hz, 1 H), 1.19 (td, *J* = 12.9, 6.3 Hz, 2 H), 1.02 (m, 2 H), 0.99 (s, 3 H), 0.93 (s, 3 H), 0.80 (s, 3 H), 0.77 (s, 3 H), 0.76 (s, 3 H), 0.73 (d, *J* = 9.7 Hz, 1 H); ^13^C NMR (CDCl_3_, 151 MHz) *δ* 176.4, 174.2, 173.6, 150.1, 135.2, 135.2, 133.9, 133.9, 130.7, 130.7, 118.9, 118.4, 118.4, 110.1, 81.0, 64.3, 62.8, 55.6, 50.5, 49.0, 47.9, 46.6, 42.9, 41.1, 38.6, 38.0, 37.8, 37.3, 34.3, 34.0, 30.0, 29.9, 29.9, 29.8, 28.2, 27.2, 25.4, 24.7, 24.6, 23.9, 23.6, 22.7, 22.7, 22.6, 22.3, 21.0, 18.4, 16.8, 16.3, 16.2, 14.9; ^31^P NMR (CDCl_3_, 243 MHz) *δ* 24.36. GC-MS: m/e: 1320.53 (100.0%), 1321.53 (88.0%), 1318.53 (51.2%), 1322.53 (49.2%), 1319.53 (44.5%), 1323.53 (42.8%), 1322.54 (39.7%), 1320.54 (19.7%), 1324.53 (18.5%), 1323.54 (11.7%), 1321.54 (6.0%), 1325.54 (5.5%), 1324.54 (3.3%), 1326.54 (1.3%). ESI-MS (30 eV): *m*/*z* 580.82 [(M-2Br^−^)/2]^+^; Anal. Calcd. for C_78_H_98_Br_2_O_4_P_2_ (Mol. Wt.: 1321.37): C, 70.90; H, 7.48; Br, 12.09; O, 4.84; P, 4.69. Found: C, 70.13; H, 7.10; Br, 12.38; O, 4.51; P, 4.73.

#### 2.2.2. Synthesis of Betulinic Acid (BA) Derivatives

**Scheme 2:**
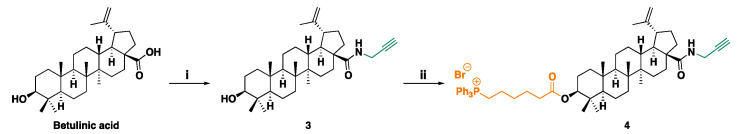
Synthesis of the BA derivatives **3** and **4**. Legend to scheme (**i**) propargylamine, THF, 2-(7-azobenzotriazole)-*N*, *N*, *N*′, *N*′-tetramethyluronium hexafluorophosphate (HATU), Et_3_N, 0 °C to r. t.; (**ii**) 6-TPPHA (**10**), DCM, DCC, DMAP, −10 °C to r. t. Chemical modifications leading to the introduction of triple-bond-containing moieties are in green color, while those leading to the insertion of the triphenyl phosphonium hexanoate group are in orange color.

BA derivatives **3** and **4** were synthesized according to Scheme 2 using the reagents described in Scheme 2’s caption.

*(3β)-3-Hydroxy-N-2-Propyn-1-Yllup-20(29)-En-28-Amide* **3**. To an ice-cooled solution of BA (40 mg, 0.088 mmol) and propargylamine (10 μL, 0.157 mmol, 1.79 equiv.) in THF (0.3 mL), 2-(7-azobenzotriazole)-*N*, *N*, *N*′, *N*′-tetramethyluronium hexafluorophosphate (HATU, 37 mg, 0.097 mmol, 1.11 equiv.) and Et_3_N (50 μL, 0.36 mmol, 4.08 equiv.) were added, and the resulting mixture was stirred at 0 °C to r. t. Upon completion of the reaction, the mixture was concentrated to dryness, and the oily residue was subjected to FCC purification using gradient PhMe:EtOAc 97:3 to 95:5 as eluent, to afford the pure product as a colorless oil (30 mg, 69% yield). R*f* (PhMe:EtOAc 97:3) = 0.09.

ATR-FTIR (ṽ, cm^−1^): 3309 (OH), 3073 (=C-H), 2940, 2867 (C-H), 1639 (C=ONH), 1505, 1447 (C-H). ^1^H NMR (CDCl_3_, 600 MHz) *δ* 5.71 (t, *J* = 5.3 Hz, 1 H), 4.73 (d, *J* = 2.3 Hz, 1 H), 4.59 (dd, *J* = 2.4, 1.4 Hz, 1 H), 4.08 (ddd, *J* = 17.5, 5.4, 2.5 Hz, 1 H), 3.98 (ddd, *J* = 17.5, 5.1, 2.5 Hz, 1 H), 3.18 (dd, *J* = 11.5, 4.8 Hz, 1 H), 3.13 (td, *J* = 11.2, 4.6 Hz, 1 H), 2.42 (ddd, *J* = 13.1, 11.5, 3.5 Hz, 1 H), 2.20 (t, *J* = 2.5 Hz, 1 H), 1.94 (m, 2 H), 1.75 (dd, *J* = 12.2, 7.8 Hz, 1 H), 1.71 (m, 1 H), 1.68 (s, 3 H), 1.65 (t, *J* = 3.6 Hz, 1 H), 1.59 (m, 3 H), 1.54 (m, 1 H), 1.50 (m, 2 H), 1.45 (m, 1 H), 1.42 (m, 2 H), 1.38 (m, 1 H), 1.35 (m, 2 H), 1.25 (m, 2 H), 1.16 (dt, *J* = 13.4, 3.2 Hz, 1 H), 1.00 (m, 1 H), 0.97 (s, 3 H), 0.96 (s, 3 H), 0.94 (s, 3 H), 0.90 (dd, *J* = 13.2, 4.1 Hz, 1 H), 0.86 (d, *J* = 7.1 Hz, 1 H), 0.81 (s, 3 H), 0.75 (s, 3 H), 0.67 (m, 1 H); ^13^C NMR (CDCl_3_, 151 MHz) *δ* 176.1, 151.1, 129.3, 128.4, 109.6, 80.4, 79.2, 71.4, 55.9, 55.6, 50.9, 50.4, 47.0, 42.7, 41.0, 39.1, 38.0, 37.4, 34.6, 33.9, 31.0, 29.6, 29.2, 28.2, 27.7, 25.8, 21.1, 19.7, 18.5, 16.4, 15.6, 14.9. GC-MS: m/e: 493.39 (100.0%), 494.40 (37.5%), 495.40 (7.2%). ESI-MS (30 eV): *m*/*z* 494.45 [M + H^+^]; Anal. Calcd. for C_33_H_51_NO_2_ (Mol. Wt.: 493.76): C, 80.27; H, 10.41; N, 2.84; O, 6.48. Found: C, 80.13; H, 10.50; N, 3.02; O, 6.42.

*(3β)-3-Hydroxy-3-(6-Triphenyl Phosphonium-Hexanoate)-N-2-Propyn-1-Ylurs-20(29)-En-28-Amide* **4**. To an ice-cooled (−10 °C) suspension of **3** (23 mg, 0.047 mmol) and **10** (23 mg, 0.050 mmol, 1.07 equiv.) in DCM (0.45 mL), a solution of DCC (10.3 mg, 0.050 mmol, 1.06 equiv.) and DMAP (2 mg, 0.016 mmol, 0.35 equiv.) in DCM (0.2 mL) was added portion-wise and the resulting mixture was stirred at −10 °C to r. t. Upon completion of the reaction, the mixture was diluted with EtOAc and washed with HCl _(aq)_ 1 M, water and brine. The organic layer was dried over anhydrous Na_2_SO_4_, filtered and concentrated to dryness, and the oily residue was subjected to FCC purification using CHCl_3_:MeOH 92:8 as eluent to afford the pure product as a colorless oil (15 mg, 34% yield). R*f* (CHCl_3_:MeOH 92:8) = 0.08.

ATR-FTIR (ṽ, cm^−1^): 3061 (=C-H), 2940, 2868 (C-H), 1721 (C=OO), 1639 (C=ONH), 1439 (C-H), 1112 (C-O), 723, 690 (C-P). ^1^H NMR (CDCl_3_, 600 MHz) *δ* 7.85 (m, 6H), 7.77 (td, *J* = 7.4, 1.7 Hz, 3 H), 7.69 (td, *J* = 7.8, 3.3 Hz, 6 H), 5.79 (br s, 1 H), 4.72 (d, *J* = 2.3 Hz, 1 H), 4.58 (m, 1 H), 4.39 (m, 1 H), 4.06 (ddd, *J* = 17.4, 5.4, 2.5 Hz, 1 H), 3.96 (ddd, *J* = 17.5, 5.1, 2.5 Hz, 1 H), 3.90 (br s, 2 H), 3.12 (td, *J* = 11.1, 4.5 Hz, 1 H), 2.52 (t, *J* = 2.5 Hz, 1 H), 2.41 (td, *J* = 12.0, 11.5, 3.6 Hz, 1 H), 2.25 (td, *J* = 7.3, 2.1 Hz, 2 H), 1.94 (dtd, *J* = 13.4, 11.0, 7.9 Hz, 3 H), 1.72 (m, 4 H), 1.67 (s, 3 H), 1.64–1.51 (m, 9 H), 1.45 (m, 3 H), 1.35 (ttd, *J* = 15.3, 11.5, 9.3, 3.8 Hz, 5 H), 1.25 (m, 3 H), 1.14 (dt, *J* = 13.4, 3.2 Hz, 1 H), 0.94 (s, 3 H), 0.92 (s, 3 H), 0.81 (s, 3 H), 0.77 (s, 6 H), 0.73 (m, 1 H); ^13^C NMR (CDCl_3_, 151 MHz) *δ* 176.2, 173.7, 151.2, 135.2, 134.1, 134.0, 130.8, 130.7, 119.2, 118.6, 109.7, 81.2, 80.6, 80.5, 71.4, 56.0, 55.8, 50.9, 50.6, 47.1, 42.8, 41.2, 38.7, 38.4, 38.2, 38.1, 37.5, 34.7, 34.4, 33.9, 31.2, 30.1, 30.0, 29.7, 29.3, 28.3, 25.9, 24.8, 24.1, 22.9, 22.8, 22.8, 22.6, 21.3, 19.8, 18.5, 16.9, 16.5, 15.0; ^31^P NMR (CDCl_3_, 243 MHz) *δ* 24.39. GC-MS: m/e: 931.47 (100.0%), 933.46 (97.3%), 932.47 (64.6%), 934.47 (63.3%), 935.47 (20.8%), 933.47 (20.7%), 936.47 (4.4%), 934.48 (4.3%). ESI-MS (30 eV): *m*/*z* 852.72 [(M-Br^−^)^+^]; Anal. Calcd. for C_57_H_75_BrNO_3_P (Mol. Wt.: 933.09): C, 73.37; H, 8.10; Br, 8.56; N, 1.50; O, 5.14; P, 3.32. Found: C, 73.23; H, 8.47; Br, 8.38; N, 1.74; O, 5.32; P, 3.70.

#### 2.2.3. Synthesis of Ursolic Acid (UA) Derivatives

**Scheme 3:**
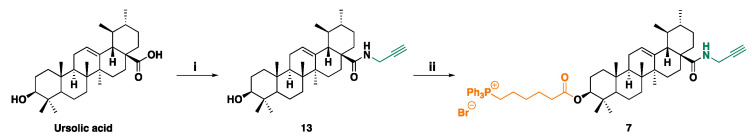
Synthesis of the UA derivative **7** and intermediate **13**. Legend to scheme: (**i**) propargylamine, THF, HATU, Et_3_N, 0 °C to r. t.; (**ii**) **10**, DCM, DCC, DMAP, −10 °C to r. t. Chemical modifications leading to the introduction of triple-bond-containing moieties are in green color, while those leading to the insertion of the triphenyl phosphonium hexanoate group are in orange color.

UA derivative **7** was synthesized according to Scheme 3 via intermediate **13**. Reagents used have been described in the legend in Scheme 3’s caption.

*(3β)-3-Hydroxy-N-2-Propyn-1-Ylurs-12-En-28-Amide* **13**. To an ice-cooled suspension of UA (50 mg, 0.109 mmol) and propargylamine (10 μL, 0.157 mmol, 1.44 equiv.) in THF (0.55 mL), HBTU (46 mg, 0.121 mmol, 1.11 equiv.) and Et_3_N (50 μL, 0.36 mmol, 3.3 equiv.) were added, and the resulting mixture was stirred at −10 °C to r. t. for 72 h. The reaction mixture was concentrated to dryness, and the oily residue was subjected to FCC purification using PhMe:EtOAc 97:3 as eluent to afford both the unreacted UA-active ester and the pure product **13** as a colorless oil (12 mg, 22% yield—51% based on recovered unreacted material). R*f* (PhMe:EtOAc 97:3) = 0.06.

^1^H NMR (CDCl_3_, 600 MHz) *δ* 6.07 (t, *J* = 4.8 Hz, 1 H), 5.36 (t, *J* = 3.6 Hz, 1 H), 4.03 (ddd, *J* = 17.6, 5.3, 2.6 Hz, 1 H), 3.89 (ddd, *J* = 17.6, 4.2, 2.6 Hz, 1 H), 3.22 (dd, *J* = 11.4, 4.6 Hz, 1 H), 2.20 (t, *J* = 2.6 Hz, 1 H), 1.98 (m, 3 H), 1.88 (m, 2 H), 1.73 (ddt, *J* = 13.6, 4.7, 2.3 Hz, 1 H), 1.63 (ddt, *J* = 13.9, 10.1, 4.2 Hz, 3 H), 1.58 (m, 2 H), 1.54 (m, 2 H), 1.46 (m, 4 H), 1.37 (dd, *J* = 12.4, 3.3 Hz, 1 H), 1.30 (m, 3 H), 1.10 (s, 3 H), 1.06 (ddd, *J* = 14.0, 4.2, 2.6 Hz, 1 H), 1.01 (m, 1 H), 0.99 (s, 3 H), 0.95 (s, 3 H), 0.93 (s, 3 H), 0.87 (d, *J* = 6.5 Hz, 3 H), 0.80 (s, 3 H), 0.78 (s, 3 H), 0.72 (dd, *J* = 11.7, 1.9 Hz, 1 H); ^13^C NMR (CDCl_3_, 151 MHz) *δ* 178.2, 140.0, 126.3, 80.0, 79.2, 71.8, 55.4, 54.0, 48.0, 47.8, 42.7, 40.0, 39.8, 39.3, 39.0, 38.9, 37.2, 37.1, 32.9, 31.1, 29.6, 28.4, 28.1, 27.4, 25.2, 23.6, 23.5, 21.4, 18.5, 17.4, 17.1, 15.8, 15.7.

*(3β)-3-Hydroxy-(6-Triphenyl Phosphonium-Hexanoate)-N-2-Propyn-1-Ylurs-12-En-28-Amide* **7**. To an ice-cooled suspension of **13** (12 mg, 0.024 mmol) and **10** (12 mg, 0.026 mmol, 1.09 equiv.) in DCM (0.1 mL), DCC (6.0 mg, 0.029 mmol, 1.2 equiv.) and DMAP (2 mg, 0.016 mmol, 0.68 equiv.) were added and the resulting mixture was stirred at 0 °C to r. t. Upon completion of the reaction, the mixture was diluted with EtOAc and washed with HCl _(aq)_ 1 M, water and brine. The organic layer was dried over anhydrous Na_2_SO_4_, filtered and concentrated to dryness, and the oily residue was subjected to FCC purification using CHCl_3_:MeOH 92:8 as eluent, to afford the pure product as a colorless oil (10 mg, 45% yield). R*f* (CHCl_3_:MeOH 92:8) = 0.08.

ATR-FTIR (ṽ, cm^−1^): 3058 (=C-H), 2962, 2924, 2869 (C-H), 1723 (C=OO), 1644 (C=ONH), 1439 (C-H), 1112 (C-O), 746, 729, 690 (C-P). ^1^H NMR (CDCl_3_, 600 MHz) *δ* 7.84 (ddd, *J* = 12.6, 8.4, 1.3 Hz, 6 H), 7.78 (td, *J* = 7.4, 1.7 Hz, 3 H), 7.69 (td, *J* = 7.8, 3.3 Hz, 6 H), 6.07 (t, *J* = 4.8 Hz, 1 H), 5.34 (t, *J* = 3.7 Hz, 1 H), 4.49 (d, *J* = 8.1 Hz, 2 H), 4.42 (m, 1 H), 4.01 (dd, *J* = 17.6, 5.3 Hz, 1 H), 3.88 (m, 1 H), 3.82 (m, 1 H), 3.47 (tdt, *J* = 11.0, 7.9, 3.9 Hz, 2 H), 2.26 (td, *J* = 7.5, 1.5 Hz, 1 H), 2.20 (t, *J* = 2.6 Hz, 1 H), 1.97 (m, 2 H), 1.89 (dd, *J* = 14.6, 5.5 Hz, 3 H), 1.84 (m, 1 H), 1.79 (s, 1 H), 1.72 (m, 2 H), 1.68 (t, *J* = 4.0 Hz, 1 H), 1.65 (m, 1 H), 1.62 (d, *J* = 7.0 Hz, 1 H), 1.59 (m, 1 H), 1.56 (m, 1 H), 1.55 (d, *J* = 3.9 Hz, 1 H), 1.50 (m, 1 H), 1.48 (m, 1 H), 1.45 (m, 1 H), 1.42 (m, 1 H), 1.35 (m, 1 H), 1.31 (m, 2 H),M 1.28 (m, 1 H), 1.14 (m, 1 H), 1.11 (m, 1 H), 1.10 (m, 1 H), 1.08 (s, 3 H), 1.04 (m, 1 H), 0.94 (s, 3 H), 0.92 (s, 3 H), 0.86 (d, *J* = 6.4 Hz, 3 H), 0.80 (s, 3 H), 0.80 (s, 3 H), 0.79 (s, 3 H); ^13^C NMR (CDCl_3_, 151 MHz) *δ* 178.1, 173.6, 157.3, 140.0, 135.2, 135.2, 134.0, 133.9, 130.7, 130.7, 126.2, 118.9, 118.3, 81.0, 79.9, 71.8, 55.4, 53.9, 49.2, 48.0, 47.7, 42.7, 39.9, 39.8, 39.3, 38.5, 37.9, 37.1, 37.0, 34.3, 34.1, 32.8, 31.0, 30.0, 29.9, 29.6, 28.3, 28.0, 25.9, 25.2, 25.2, 24.8, 23.8, 23.6, 23.5, 22.7, 21.4, 18.3, 17.5, 17.1, 17.0, 15.8; ^31^P NMR (CDCl_3_, 242.9 MHz) *δ* 24.38. GC-MS: m/e: 931.47 (100.0%), 933.46 (97.3%), 932.47 (64.6%), 934.47 (63.3%), 935.47 (20.8%), 933.47 (20.7%), 936.47 (4.4%), 934.48 (4.3%). ESI-MS (30 eV): *m*/*z* 852.71 [(M-Br^−^)^+^]; Anal. Calcd. for C_57_H_75_BrNO_3_P (Mol. Wt.: 933.09): C, 73.37; H, 8.10; Br, 8.56; N, 1.50; O, 5.14; P, 3.32. Found: C, 73.15; H, 8.43; Br, 8.37; N, 1.78; O, 5.43; P, 3.63.

### 2.3. ATR-FTIR Spectroscopy of BA, BET, UA and Compounds ***1***–***7***

FTIR spectra of BA, BET and UA and compounds **1**–**7** were recorded in triplicate directly on the solid samples in attenuated total reflection (ATR) mode. Acquisitions were made from 4000 to 600 cm^−1^, with 1 cm^−1^ spectral resolution, co-adding 32 interferograms, with a measurement accuracy in the frequency data at each measured point of 0.01 cm^−1^, due to the laser internal reference of the instrument. The frequency of each band was obtained automatically by using the “find peaks” command of the instrument software.

### 2.4. Multivariate Analysis of ATR-FTIR and ^13^C NMR Spectral Data

#### 2.4.1. ATR-FTIR Spectral Data

ATR-FTIR data (transmittance, %) of all acquired spectra were arranged in a matrix of 3401 (wavenumbers cm^−1^) × 10 (compounds) = 34,010 measurable variables. Then, spectral data of previously reported triphenyl phosphonium salt (BPPB) were added to this matrix [8], obtaining a second dataset of 3401 (wavenumbers cm^−1^) × 11 (compounds) = 37,411 measurable variables. For each sample, the variables consisted of the values of transmittance (%) associated with the wavenumbers (3401) in the range of 4000–600 cm^−1^. The systems were simplified by exploiting the multivariate analysis, named principal component analysis (PCA), processing each matrix of spectral data using CAT (Chemometric Agile Tool, freely available online at https://www.gruppochemiometria.it/index.php/software/19-download-the-r-based-chemometric-software, accessed on 11 November 2025). Before PCA, ATR-FTIR spectral data were scaled and centered. The results were reported as a score plot of PC1 vs. PC2 and discussed in Section 3.

#### 2.4.2. ^13^C NMR Spectral Data

^13^C NMR values of δ (ppm) of all peaks present in the NMR spectra of analyzed samples were first arranged in a matrix of 52 (δ, ppm) × 15 (compounds) = 780 measurable variables. Then, since the number of ^13^C NMR signals was very different for analyzed compounds, the resulting matrix contained an excessive number of missing data, which did not allow us to calculate the system variance and carry out PCA. To address this issue, the number of missing data was reduced by reducing the range of possible δ (ppm). A second matrix was then constructed, made of 34 (δ, ppm) × 15 (compounds) = 510 measurable variables. For each sample, the variables consisted of the values of δ (ppm) of all peaks present in its ^13^C NMR spectrum. The system was simplified by exploiting PCA, processing the matrix of spectral data using CAT (Chemometric Agile Tool, freely available online at https://www.gruppochemiometria.it/index.php/software/19-download-the-r-based-chemometric-software, accessed on 10 November 2025). Before PCA, ^13^C NMR spectral data were scaled and centered. The results were reported as a score plot of PC1 vs. PC3 and discussed in Section 3.

### 2.5. Potentiometric Titrations of Compound ***1*** and ***4***–***7***

The potentiometric titration of TPP-containing compounds was carried out in a non-aqueous medium (mixture of anhydrous acetic acid (AcOH) and acetic anhydride (Ac_2_O) 30:70 (*v*:*v*)) with HClO_4_, performing a slightly modified procedure previously described by us for the volumetric titration of ammonium salts [25,26,27]. A similar protocol was in fact described by Pifer and Wollish, who applied this method for salts of weak organic bases [28]. Briefly, the exacted weighted samples of compounds **1** and **4**–**7** were dissolved in AcOH:Ac_2_O 30:70, treated with a solution of mercury acetate (1.5 g) in AcOH (25 mL), and titrated with a standardized 0.1 N solution of HClO_4_ in AcOH:Ac_2_O, prepared as described in the following section, using potentiometric endpoint detection. The titrations were performed under efficient stirring with a magnetic stirrer, at room temperature (25 ± 2 °C). Millivolts were measured at fixed points up to the addition of 6 mL of 0.1 N HClO_4_. Titrations were made in triplicate, and the measurements were reported as mean ± SD.

#### Preparation of a 0.1 M Perchloric Acid Volumetric Solution

The 0.1 M perchloric acid volumetric solution was prepared by diluting 8.5 mL of 70–73 wt% perchloric acid with 900 mL of anhydrous acetic acid and 30 mL of acetic anhydride and then diluting to 1000 mL with anhydrous acetic acid. Perchloric acid was standardized by titration against potassium hydrogen phthalate [29].

### 2.6. Optical Microscopy Analyses

Optical microscopy analyses were performed in the laboratory of Professor Paolo Giordani (https://rubrica.unige.it/personale/VUZCU11t, accessed on 25 November 2025) of the Department of Pharmacy (https://rubrica.unige.it/strutture/struttura/100006, accessed on 25 November 2025) of the University of Genoa (SSD BIOS-01/D Pharmaceutical Biology). m-Q Water solutions of compounds **1** and **4**–**7**, possessing the triphenyl phosphonium (TPP) salt group, were investigated via optical microscopy (OM) analysis to assess their possible capability to give spherical vesicles. In the performed experiments, 0.6–1.5 mg of solid compounds were dissolved in m-Q water (44–110 µL) by gentle heating, obtaining clear solutions with a concentration of 13.6 mg/mL. Upon cooling, the obtained solutions were observed using a Leica DM750 optical microscope (Leica Italy, Milan, Italy) equipped with 40× and 100× objectives. The camera used for image capture was a Leica ICC50W (Leica Italy, Milan, Italy). All images were processed using LAS EZ 3.4.0. software (Leica Italy, Milan, Italy).

### 2.7. Dynamic Light Scattering (DLS) Analysis

The particle size, intended as hydrodynamic diameter distribution, polydispersity index (PDI) and zeta potential (ζ-p) (mV) of compound **6,** which demonstrated spherical vesicles at OM, was measured at 25 °C, at a scattering angle of 90° in m-Q water using a Malvern Nano ZS90 light scattering apparatus (Malvern Instruments Ltd., Worcestershire, UK). The solution of **6** used for OM (9.8 mM) was diluted to a final concentration of 5 mM (8.4 kcps) and analyzed. The *ζ-p* value of BPPB was recorded using the same apparatus at a count rate of 20–59 kcps. The results of the experiments are presented as the mean of 3 independent determinations, made of 10 runs (particle size) or 12 runs (ζ-p), each one ± SD. Intensity-based results have been reported to express particle size distribution.

### 2.8. Antibacterial Activity

With the aim of developing novel compounds active against MDR clinical isolates, preliminary experiments were conducted to evaluate the inhibitory effects of the synthesized compounds **1**–**7**, as well as those of pure BET, BA and UA by determining their minimum inhibitory concentrations (MICs) on a selection of Gram-positive and Gram-negative species. All bacteria used in this study were clinical isolates that had developed resistance to at least one or two antibiotics, including also ESKAPE species (*Enterococcus faecium*, *Staphylococcus aureus*, *Klebsiella pneumoniae*, *Acinetobacter baumannii*, *Pseudomonas aeruginosa* and *Enterobacter* spp.). The main objectives were to understand if chemical modifications would have improved the activity of pristine compounds and then select the best-performing compounds to be used for further experimentation.

#### 2.8.1. Bacterial Strains

A total of seven isolates were used in this study. They were derived from a collection of MDR Gram-positive and Gram-negative species isolated in S. Martino Hospital of Genoa (Ospedale Policlinico S. Martino, Largo Rosanna Benzi, 10, San Martino d’Albaro, Genova, Italy, https://www.ospedalesanmartino.it/it/, accessed on 25 November 2025) and were kindly gifted to the University of Genova for research purposes. All were clinical strains isolated from infected human specimens hospitalized at S. Martino Hospital and identified using VITEKR 2 (Biomerieux, Firenze, Italy) or matrix-assisted laser desorption–ionization time-of-flight (MALDI-TOF) mass spectrometric technique (Biomerieux, Firenze, Italy). We declare that no human being was used for this study, but only bacterial species isolated for diagnosis purposes. The seven MDR isolates included four Gram-positive and three Gram-negative bacteria of different genera. Among the species of Gram-positive bacteria, two were enterococci (one *Enterococcus faecalis* and one *E. faecium*), while two were staphylococci (one *Staphylococcus aureus* and one *S. epidermidis*). All enterococci were MDR isolates with resistance to vancomycin (VRE) and teicoplanin, while all staphylococci were MDR strains with resistance to methicillin (MRSA and MRSE). Gram-negative species included one non-fermenting isolate of *Pseudomonas aeruginosa* isolated from cystic fibrosis patients with resistance to carbapenems. Two strains were Enterobacteriaceae, including one *Escherichia coli* and one *Klebsiella pneumoniae*, which were resistant to carbapenems and *K. pneumoniae* carbapenemase (KPC)-producing bacteria. Table 1 summarizes the bacterial strains used in this study and their resistance profiles.

#### 2.8.2. Determination of the Minimum Inhibitory Concentrations (MICs)

To investigate the antibacterial activity of BPPB on the described pathogens, their minimum inhibitory concentrations (MICs) were determined by following the microdilution procedures detailed by the European Committee on Antimicrobial Susceptibility Testing EUCAST [23] and reported in our previous works [30].

## 3. Results and Discussion

### 3.1. Synthesis of Triterpenoid Derivatives

With the aim of finding novel compounds effectively active against difficult-to-treat clinically isolated superbugs, seven triterpenoid derivatives (**1**–**7**) were synthesized by chemical modifications of betulin (BET), betulinic acid (BA) and ursolic acid (UA). As indicated by the literature, which evidenced that several UA, BET and BA derivatives with enhanced potency, bioavailability and water solubility (including esters, amides, oxadiazole, quinolone, etc.), mostly derived from modifications at positions C-3 (hydroxyl), C-12-C-13 (double bonds) and C-28 (carboxylic acid or primary alcoholic groups) [31], we carried out chemical modification of parent compounds at position C-28 or C-28 and C-3, as reported in a recent study for preparing BET [32] and BA derivatives [33]. In particular, it has been reported that structural modifications that transformed the C-28 carboxyl group into ester or amide groups, as well as ester modifications or oxidations of C-3 hydroxyl, could enhance the cytotoxicity of BA [33]. Organic fragments, such as propargyl amine (PAM), propiolic acid (PA) and 6-triphenyl phosphonium hexanoic acid (6-TPPHA), bearing the triphenyl phosphonium group (TPP), were selected as modifying groups. PA was selected and introduced on hydroxyl in C-28 of BET, obtaining the ester derivatives **2**. This choice was promoted by papers by Chrobak et al. [32] and Csuk et al. [34]. Authors reported that the introduction of PA in such a position or a carbonyl group at C-28 and a short substituent with a terminal triple bond, improved significantly BET activity against most of the tested cancer lines at a concentration in the range of 0.35–18.7 µM [32]. PAM was selected to modify BA and UA on hydroxyl in C-28, thus preparing the C-28 propargyl amide BA derivative **3** and the C-28 propargyl UA derivative **13**, which were also the intermediates to obtain the TPP-BA derivative **4** and the final UA derivative **7**. These projects were based on a paper that reported compound **3** to have high anticancer activity against human T47D (breast cancer), SW707 (colorectal adenocarcinoma) and mouse P388 (leukemia) cell lines [35]. Compound **3** was four times more cytotoxic than BA against the human SW707 cell line, also establishing that BA derivatives with a shorter alkynyl chain are more cytotoxic [35]. The prop-2-ynyl-carbamate group was chosen to insert on the BET scaffold a carbamate residue in a two-step reaction and achieve BET-carbamate derivative **9**. In this regard, Wiemann et al. have reported that BET-derived carbamates are interesting scaffolds for the synthesis of novel cytotoxic compounds [36]. Specifically, authors evidenced that anticancer activity of some BET-derived mono- and bis-carbamates against different cancer cell lines was higher than that of pristine BET, while toxicity on normal fibroblasts was lower [36]. The 6-TPP-hexanoic acid (**10**) was selected as the carboxylic acid moiety for the esterification of BET derivative **9**, BA derivative **3** and UA derivative **13**, achieving the TPP-containing triterpenoids **1**, **4** and **7**. This further modification on C-3 hydroxyl was carried out because it has been recently reported that compounds bearing the TPP group possess potent antibacterial effects against several clinical superbugs, despite their complex pattern of resistance [8,30]. Based on this evidence, the TPP-hexanoate group was inserted only in C-3, as well as in both C-3 and C-28 positions of BET, via a two-step reaction, achieving BET derivatives without triple bonds **5** and **6**. In fact, it has also been observed that a compound having two TPP groups, as in the case of **6**, linked by a C-12 chain and capable of giving nanovesicles in water (BPPB), possessed antibacterial effects [8], remarkably higher than those of another compound (namely **1** in the paper) possessing only one TPP moiety [30]. Finally, it was observed that, differently from the previously reported compound **1**, active only against Gram-positive species, BPPB-bearing two TPP groups, was also very potent against superbugs of Gram-negative species [8]. Additionally, experiments carried out with both **1** and BPPB to assess their cytotoxic and haemolytic effects using several mammalian cell lines and red blood that cells evidenced soft cytotoxicity and low haemolytic toxicity, respectively [8,30]. These data established that the TPP group could be an appealing candidate to chemically modify BET, BA and UA and to provide compounds active against bacteria with potential for future clinical development. Concerning the selected triterpenoid nuclei, BET, BA and UA were chosen because naturally occurring and are devoid of evident intrinsic cytotoxicity [37,38,39,40,41,42,43,44,45]. These characteristics could have possibly further softened the sharp cationic characteristic of the TPP group, rationally responsible for the residual cytotoxicity of previously reported compounds containing it, thus reducing or nullifying it, while maintaining the antibacterial properties. In this regard, BA was selected, since it was found to be devoid of toxicity in normal cells [37,38,39]. When it was used in the 24 h treatment of human immortalized keratinocytes (HaCaT), the percentage of viable cells calculated at the highest concentration tested (50 μM) was >81% [40]. BET was chosen, since it has been reported to be selectively toxic towards neoplastic cells, but only weakly toxic towards normal cells [41,42,43]. UA was finally selected because it is reported to have only weak cytotoxic effects on normal cells [44]. There are examples of single-dose subcutaneous injections of 300 mg/kg of UA, which did not cause any deviations in the clinical hematology parameters and tissue morphology of animal models. Additionally, 5-day short-term toxicity studies about the combined UA and oleanolic acid administration at a dose of 1.0 mg/kg did not lead to any morbidity or mortality [45]. Moreover, in a recent long-term (90 days) oral toxicity in vivo study, UA was given to Han-Wistar rats at a repeated dose of 0, 100, 300, or 1000 mg/kg/day to assess its safety and toxicity [45]. No mortality, uncharacteristic body weight changes, or tissue architecture variations were observed at any of the analyzed test doses. Additionally, no changes in behavior, or hematological and clinical parameters were evidenced, suggesting the safe and non-toxic nature of UA [45]. Moreover, BET and BA, while excellent multi-target natural compounds capable of simultaneously improving several health disorders, are totally inactive and not usable against both Gram-positive and Gram-negative species as effective antibacterial agents. Therefore, they were selected to evaluate if, upon our chemical modifications, it would be possible to reevaluate them also as potent new antibacterials. Conversely, UA, already known as a remarkable antibacterial for treating Gram-positive bacteria, was selected to assess if by inserting the TPP group, its spectrum of action could be enhanced and enlarged also to Gram-negative superbugs. In the following, Section 3.1.1, Section 3.1.2 and Section 3.1.3, the synthetic procedures and NMR characterization of BET, BA and UA intermediates and final derivatives tested as antibacterial agents have been discussed. Copies of NMR spectra of compounds discussed in the following, Section 3.1.1, Section 3.1.2 and Section 3.1.3, are available in Section S1.2 (Supplementary Materials) as Figures S1.2.1–S1.2.27. As readers could observe in the description of the synthetic procedure used to prepare compounds **1**–**7**, sometimes both the yield of intermediates and the overall yields were low. Especially for the esterification reactions and amide synthesis using condensing agents as DCC, the formation of side products, such as the acyl ureic derivatives, can occur depending on conditions [26], which can strongly limit the final yield. Additionally, as a correct procedure in organic synthesis, we have isolated and purified all the intermediates by laborious FCCs. Such a purification work-up provides compounds with a high level of purity, but can limit their yield, because some material can be retained in the column by the stationary phase, especially in the case of most of our compounds, which are highly polar salts.

#### 3.1.1. Synthesis of BET Derivatives

*(3β)-Lup-20(29)-Ene-3,28-Diol, 28-(4-Nitrophenyl Carbonate)* **8**. BET derivative intermediate **8** is known and was prepared in 83% yield, following the procedure described by Laiolo et al. [46]. The white foam obtained after FCC purification as described in Section 2 was subjected to ^1^H and ^13^C NMR analysis, which provided spectral data in agreement with those reported by Laiolo et al., thus confirming the structure and high level of purity of **8** [46]. In this reaction, the stoichiometry of reagents and temperature are of paramount importance to obtain the desired C-28 mono-substituted derivative. In fact, a procedure like that of Laiolo et al. but carried out working at room temperature instead of 0 °C and with 2.2 equivalents excess of 4-nitrophenyl chloroformate, instead of 1.04 equivalents, allowed Liu et al. to obtain the C-3, C-28 di-(4-nitrophenyl carbonate) BET derivative (3β)-lup-20(29)-ene-3,28-diol and 3,28-di-(4-nitrophenyl carbonate) in good yield of 74% [47]. NMR characterization of **8** evidenced that, with respect to the ^1^H NMR spectrum of BET, that of **8** presented new peaks at 8.29, 8.28, 7.41 and 7.39 ppm, due to the AA’-BB’ aromatic *p*-di-substituted system given by the introduction of the nitrophenyl carbamate group on C-28. The double doublet signal of the diastereotopic protons of the CH_2_OH group, which were at 3.34 and 3.81 ppm in the spectrum of BET with a *J* = 10.8 Hz [48], shifted to a higher value of *δ* in the spectrum of **8** (4.51 and 4.08 ppm), maintaining the same multiplicity with a *J* of about 10.8 Hz. Similarly, in the ^13^C NMR spectrum of **8**, signals for the aromatic carbon atoms were detected at 155.6, 145.3 ppm (quaternary C) and 125.3, 121.8 (CH=CH), while the signal of C=O of the carbamate group was found at 153.0 ppm. The C-28 methylene signal, with respect to the position occupied in the spectrum of BET (60.47 ppm) [48], in the spectrum of **8** is shifted to a higher value of *δ* (68.30 ppm).

**Figure 2:**
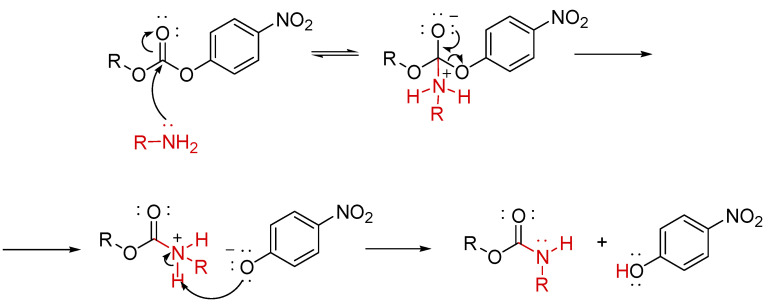
Mechanism of carbonate aminolysis. R = organic residues.

*(3β)-Lup-20(29)-Ene-3,28-Diol, 28-(N-Propargyl Carbamate)* **9**. BET derivative intermediate **9** was unknown. It was prepared via aminolysis of the BET derivative **8** following the modified procedure described by Kemmer et al. [49]. With this approach, the authors prepared a novel dextran norbornene methylcarbamate, completely converting the dextran 4-nitrophrnyl carbonate (DNPC), used as starting material, by its reaction with 5-norbornene-2-methylamine in a ratio of 1:2, at 20 °C for 5 h in DMF [49]. Here, **8** and propargylamine were reacted in a similar ratio 1:1.9 in the presence of Et_3_N, but DMF was replaced by THF, since it is easier to remove under vacuum after reaction. The reaction mixture was stirred at room temperature to completion. According to what is reported by different authors [36,50], the reaction proceeds according to the following two-step process (Figure 2).

Practically speaking, the carbamate formation involves the acyl nucleophilic substitution of the 4-nitro-phenoxyl group by the amine reagent, with expulsion of 4-nitro-phenoxide, thus obtaining a protonated carbamate, which transforms into the final carbamate by deprotonation operated by 4-nitro-phenoxide, providing 4-nitro-phenol [50]. The reactions are proposed to proceed through a stepwise mechanism with a change in the rate-determining step (RDS), based on the curved Brønsted-type plots [50]. Concerning NMR analyses of **9**, in both ^1^H and ^13^C NMR spectra, except for small traces, the aromatic signals disappeared. On the contrary, the signal of NH appeared in the form of broad singlet at 4.95 ppm, the double doublet signal of diastereotopic proton atoms of C-28 methylene (*J* = 10.8 Hz) was now detected at 4.27 and 3.86 ppm, the signal of methylene of propargyl group was detected at 3.98 ppm in form of broad singlet due to the coupling with the NH proton, while the signal of ≡C−H (the red color indicates the atom responsible of the signal) was found at 2.24 ppm. In the ^13^C NMR, the signal of C=O of carbonate of **8** at 153 ppm disappeared and was substituted by the new signal of carbamate of **9** at 157.0 ppm. The signal of methylene in C-28 was found at 64.0 ppm, that of C≡C−H was found at 80.1 ppm, while that of ≡C−H was found at 71.9 ppm. Copies of spectra of **9** are available in Section S1.2 (Supplementary Materials) as Figures S1.2.1 and S1.2.2.

*(3β)-Lup-20(29)-Ene-3,28-Diol-(6-Triphenyl Phosphonium Hexanoate)-28-(N-propargyl carbamate)* **1**. BET derivative **1** was unknown, and it was synthesized starting from **9** by a Steglich esterification reaction, carried out as in other syntheses of this study. In this case, 6-triphenyl phosphonium hexanoic acid **10** was the acid component, while DCC and DMAP were the condensation promoter and the catalyst, respectively. Compound **1** was achieved in a >50% yield and with a high level of purity. The structure of **1** was confirmed by ATR-FTIR, ^1^H, ^13^C and ^31^P NMR, as well as by GC-MS, ESI-MS, elemental analysis and potentiometric titrations, which confirmed its molecular weight. In the ^1^H NMR spectrum of compound **1**, aromatic signals integrable for 15 proton atoms appeared at 7.84, 7.77 and 7.68 ppm, due to the introduction of the triphenyl phosphonium (TPP) group present on C-6 of the new hexanoate inserted on C-3. The signal of C-3 CH shifted from the δ value of 3.18 ppm, found in the spectrum of compound **9**, to 4.39 ppm, in the spectrum of **1**. Five additional methylene signals appeared in the range 3.84–1.25 ppm, due to the alkyl chain of hexanoate. They were dispersed among the signals of CH and CH_2_ groups of pentacyclic structure of BET and of CH_2_ groups already present in **9**, and were thus of critical attribution. The signals at 3.84 and 2.24 ppm, accounting for 3 proton atoms each, unequivocally contained the signals of the two proton atoms of CH_2_P^+^ and CH_2_C=O groups, respectively. Similarly, in the ^13^C NMR of **1**, despite the C=O of carbamate being no longer visible, for the low intensity of the spectrum, the new signal of the C=OO of hexanoate was clearly observable at 173.6 ppm. New aromatic signals, some of which were in the form of a doublet, due to the coupling with the phosphorous atom, appeared at 135.2 (3 equivalent CH=), 134.0, 133.9 (6 equivalent CH=), 130.7 (6 equivalent CH=) and 118.9, 118.3 (3 equivalent quaternary C) ppm. The signal of the C-3 was found at 81.0 ppm, the signals of CH_2_C=O and of the CH_2_P^+^ groups were found at 34.3 and at 22.7 ppm, respectively, while in the ^31^P NMR, a singlet signal was observed at 24.38 ppm. Copies of spectra of **1** are available in Section S1.2 (Supplementary Materials) as Figures S1.2.3–S1.2.5.

*(3β)-Lup-20(29)-Ene-3,28-Diol, 28-(2-Propynoate)* **2**. BET derivative **2** was prepared according to the procedure described by Boryczka et al. in the year 2013 [51] and reproposed later by Bȩbenek et al. [52], achieving the desired compound in 38% yield vs. the 60% claimed by Boryczka et al. [51]. Another discrepancy with compounds previously prepared consisted in the physical state of **2**, which was obtained as a colorless oil after FCC by us, instead of as a white solid with melting points of 132–134 °C [52] and 133–135 °C [51], reported by other authors. The structure and purity of **2** were confirmed by more reliable analyses, including ATR-FTIR, GC-MS, ESI-MS and elemental analyses, as well as ^1^H and ^13^C NMR. Specifically, with respect to the ^1^H NMR spectrum of BET, where the double doublet signal of diastereotopic proton atoms of methylene C-28 (CH_2_OH) is usually present at 3.81 and 3.34 ppm, with a *J* of about 10.80 Hz [48], in the spectrum of **2**, where the propyne ester has been inserted in C-28, such a signal shifted to higher values of δ (4.37 and 3.98 ppm) with *J* = 11.1 Hz, maintaining the same multiplicity. Moreover, a new signal at 2.89 ppm was found, due to the new ≡C−H group of propine. In the ^13^C NMR spectrum of **2**, new signals with respect to those observed in the spectrum of BET were found at 153.5 ppm (C=OO), 75.0 ppm (-C≡C−H) and 74.9 ppm (-C≡C−H), while the signal of methylene C-28, usually at 60.47 ppm in BET [48], in the spectrum of **2,** was found at 65.1 ppm. Copies of spectra of **2** are available in Section S1.2 (Supplementary Materials) as Figures S1.2.6 and S1.2.7.

*(3β)-Lup-20(29)-Ene-3,28-Diol, 28-(6-Bromo Hexanoate)* **11**. BET derivative **11** was unknown, but analogous compounds with shorter alkyl chains, such as (3β)-lup-20(29)-ene-3,28-diol, 28-(5-bromopentanoate) [53,54,55], (3β)-lup-20(29)-ene-3,28-diol, 28-(4-bromobutanoate) [54,55], (3β)-lup-20(29)-ene-3,28-diol, 28-(3-bromopropanoate) [55], (3β)-lup-20(29)-ene-3,28-diol, 28-(2-bromoethanoate) [54], (3β)-lup-20(29)-ene-3,28-diol and 28-chloroacetate) [56] have been reported. Except for the case of (3β)-lup-20(29)-ene-3,28-diol, 28-chloroacetate), which was prepared by Kommera et al. by reacting BET with chloroacetic anhydride in DCM and stirring with full microwave power (250 W) at 90 °C under a maximum pressure of 10 bar for 20 min [56], all other BET derivatives were prepared starting by the proper brome alkyl acid in DCM, by Steglich-type esterification, using a condensing agent, which was EDC for Ye et al. [53] and DCC for Tsepaeva et al. [54,55], and DMAP as catalyst. The monoesters were achieved in very variable yields, from 40% for Ye et al. [53], to yields in the range of 45–95%, as reported by Tsepaeva et al. [54,55]. Specifically, we followed the procedure described by Tsepaeva et al., immediately using 3 equivalents excess of bromoacid to promote in a single reaction the formation of both mono- and diester derivatives, thus achieving the never reported monoester **11** (57% isolated yield) in the mixture with the unknown diester **12** (31% isolated yield) with an overall yield of 88%, which were separated by FCC. Note that, while the formation of diesters was reported by Tsepaeva et al. [54,55], when an excess of brome acids 1:2 was used, the BET di-brome-pentanoate was not reported by Ye et al., despite their excess 4:1 of 5-bromo-pentanoic acid [53]. Isolated compound **11** was characterized by ^1^H and ^13^C NMR spectroscopy analysis, which confirmed its structure and high level of purity. In particular, while in the ^1^H NMR spectrum of BET, the diastereotopic proton atoms of methylene C-28 provided a signal at 3.81 and 3.34 ppm (*J* = 10.80 Hz) [48], in the spectrum of **11**, such a signal shifted to 4.26 and 3.84 ppm (*J* = 11.1 Hz), as reported by Tsepaeva et al. [54]. Five additional methylene signals appeared, due to the alkyl chain of the 6-bromo-hexanoate group on C-28, which were dispersed among the signals of CH and CH_2_ groups of BET, and were thus of critical attribution. The attribution was possible for the CH_2_Br and CH_2_C=O groups, whose signals were found at 3.40 and 2.34 ppm, respectively. Similarly, in the ^13^C NMR of **11**, the signal of C-28 appeared at 62.9 ppm (60.47 ppm in BET), in agreement with findings of Tsepaeva et al. for their BET pentanoate derivative [54]. Moreover, the signals of C=OO, CH_2_C=O and CH_2_Br groups were found at 174.1, 33.7 and 32.6 ppm, respectively. Copies of spectra of **11** are available in Section S1.2 (Supplementary Materials) as Figures S1.2.13 and S1.2.14.

*(3β)-Lup-20(29)-Ene-3,28-Diol, 28-(6-Triphenyl Phosphonium-Hexanoate)* **5**. BET-TPP-derivative **5** was unknown, but analogous compounds with shorter alkyl chains, such as (3β)-lup-20(29)-ene-3,28-diol, 28-(5-triphenyl phosphonium pentanoate) [53,54], (3β)-lup-20(29)-ene-3,28-diol, 28-(4-triphenyl phosphonium butanoate) [52] and (3β)-lup-20(29)-ene-3,28-diol, 28-(2-bromoethanoate) [54], have been reported. They were prepared following the same procedure, based on a S_N_2 nucleophilic substitution of bromo atoms, using Ph_3_P as nucleophile, in CH_3_CN at reflux. Such a procedure was followed by us, but replacing acetonitrile with toluene. Compound **5** was obtained in 36% yield after FCC isolation, which was a yield significantly higher than that reported by Ye et al. (15%) [53], but lower than that reported by Tsepaeva et al. (95%) [54]. The structure of **5** and its high level of purity were confirmed by ATR-FTIR, ^1^H, ^13^C and ^31^P NMR, as well as by GC-MS, ESI-MS, elemental analysis and potentiometric titrations, which confirmed its molecular weight. Concerning the NMR spectra, the ^1^H and ^13^C NMR signals of the CH_2_ Br group were substituted by those of CH_2_P^+^ groups, which were found at 3.87 ppm instead of 3.40 ppm in the proton spectrum, and at 22.5 instead of 32.6 ppm in the carbon spectrum. More importantly, new aromatic CH signals were found in the ^1^H NMR spectrum of **5** at 7.84, 7.78 and 7.69 ppm, due to the 15 proton atoms of the new TPP group. Similarly, new signals, sometimes in the form of a doublet, for the coupling with a phosphorous atom, were found in the ^13^C NMR spectrum of **5** at 135.0 (3 equivalent CH=), 133.8, 133.7, 133.7, 133.6 (6 equivalent CH=), 130.5, 130.5, 130.4 (6 equivalent CH=), 118.7, and 118.1 (3 equivalent quaternary C) ppm. The ^31^P NMR spectrum of 5 evidenced a single signal at *δ* = 24.41. Copies of spectra of **5** are available in Section S1.2 (Supplementary Materials) as Figures S1.2.17–S1.2.19.

*(3β)-Lup-20(29)-Ene-3,28-Diol, 3,28-di-(6-Bromo Hexanoate)* (**12**). BET derivative **12** was herein prepared for the first time. Analogous compounds with shorter alkyl chains, such as (3β)-lup-20(29)-ene-3,28-diol, 28-di-(5-bromopentanoate), (3β)-lup-20(29)-ene-3,28-diol, 28-di-(4-bromobutanoate) and (3β)-lup-20(29)-ene-3,28-diol, 28-(2-bromoethanoate), have been reported by Tsepaeva et al. [54,55]. The authors achieved the desired diesters starting from BET and an excess of 2:1 of the proper brome alkyl acid dissolved in DCM, using DCC as a condensing agent and DMAP as a catalyst. The diesters were achieved in 85, 60 and 70% yields [54,55]. BET di-(6-bromo hexanoate) prepared by us using an excess 3:1 of bromo acid in the same condition as Tsepaeva et al. was not prepared previously, and the attempt of Tsepaeva et al. to prepare the BET-di-3-bromo propionate failed [55]. Compound **12** was characterized by ^1^H and ^13^C NMR spectroscopy analysis, which confirmed its structure and high level of purity. In the ^1^H NMR spectrum of **12**, the value of δ of the signal of methylene C-28 did not change significantly (4.27 and 3.84 ppm, *J* = 11 Hz). The signals for the two CH_2_C=O groups were found in the form of multiplet integrable for 4 protons at 2.33 ppm, while those of the CH_2_Br groups appeared as triple doublet, integrable for 4 protons at 3.40 ppm. The signal of CH-3 shifted from δ value of 2.19 ppm (BET) [48] to δ = 2.44 ppm. In the ^13^C NMR spectrum of **12**, distinct signals at 174.1 and 173.5 ppm were found for the 2 C=OO groups, at 34.4 and 34.3 ppm for the CH_2_C=O groups and as repeated signal at 32.6 ppm for the CH_2_Br groups, while the signal of C-3 shifted from the δ value typical of BET (78.96 ppm) [48] to an higher value (δ = 81.0 ppm). Copies of spectra of **12** are available in Section S1.2 (Supplementary Materials) as Figures S1.2.15 and S1.2.16.

*(3β)-Lup-20(29)-Ene-3,28-Diol, 3-28-di-(6-Triphenyl Phosphonium-Hexanoate)* **6**. As in the case of BET-TPP-derivative **5**, BET TPP-derivative **6** was unknown. Three compounds like **6** were prepared by Tsepaeva et al., starting from the (3β)-lup-20(29)-ene-3,28-diol, 28-di-(5-bromopentanoate), (3β)-lup-20(29)-ene-3,28-diol, 28-di-(4-bromobutanoate) and (3β)-lup-20(29)-ene-3,28-diol, 28-(2-bromoethanoate), via their rection with Ph_3_P in acetonitrile at refluxing temperature, achieving the desired products in 90%, 83% and 80% yield [54]. Using the same procedure, but replacing acetonitrile with toluene, we prepared compound **5** in 30% yield after FCC isolation. The structure of **6** and its high level of purity were confirmed by ATR-FTIR, ^1^H, ^13^C and ^31^P NMR, as well as by GC-MS, ESI-MS, elemental analysis and potentiometric titrations, which confirmed its molecular weight. Concerning the NMR spectra of **6**, the ^1^H and ^13^C NMR signals of two CH_2_Br groups were substituted by those of two CH_2_P^+^ groups, which were found at 3.83 ppm in the form of a triple doublet (4H) instead of at 3.40 ppm (proton spectrum) and at 22.6 ppm instead of 32.6 ppm in the carbon analysis. More importantly, new aromatic CH signals were found in the ^1^H NMR spectrum of **6** at 7.83, 7.78 and 7.69 ppm, due to the 30 proton atoms of the new TPP groups. Similarly, new signals sometimes in form of doublet, for the coupling with phosphorous atom, were found in the ^13^C NMR spectrum of **6** at 135.2 (6 equivalent CH), 133.9 (12 equivalent CH=), 130.7 (12 equivalent CH), 118.9, 118.4, 118.4 (6 equivalent quaternary C), ppm. The ^31^P NMR spectrum of **6** evidenced a single signal at *δ* = 24.36 ppm. Copies of spectra of **6** are available in Section S1.2 (Supplementary Materials) as Figures S1.2.20–S1.2.22.

*6-(Tri-Phenyl Phosphonium)-Hexanoic Acid* (**10**). Compound **10** is known and commercially available. It was previously prepared by different authors following similar procedures, with slight differences in terms of stoichiometry, temperature, times of reaction and purification work-up [57,58,59,60]. Additionally, the synthesis of **10** was described in paragraphs 000561-000563 of the patent by Han, Okamoto and Olson (Current Patent Assignee: REGENERON PHARMACEUTICALS-WO2022/56494, 2022, A1), which was followed by us. The method consisted of reacting 6-bromohexanoic acid with a slight excess of PPh_3_ in toluene under stirring at 120 °C for 12 h. It is curious that, according to what is reported in the literature, compound **10** was always obtained in very high yield (>90%), regardless of the different conditions [57,58,59,60], while in the patent, it was obtained in 56.4% yield, as in our case. Compound **10** was isolated as a white solid and used as such in subsequent reactions.

#### 3.1.2. Synthesis of BA Derivatives

*(3β)-3-Hydroxy-N-2-Propyn-1-Yllup-20(29)-En-28-Amide* **3**. BA derivative **3** was known, and it, as well as its betulonic analogous, were prepared by Deng et al. and Bȩbenek et al. [35,61]. Without transforming BA into its chloride derivative to be reacted with propargyl amine, as reported by Bȩbenek et al., to prepare the amide derivative of betulonic acid, compound **3** was directly obtained from BA following a procedure like that used by Thi et al., with substantial differences. While Thi et al. used DCC, 1-hydroxybenzotriazole and *N*,*N*-diisopropylethylamine (DIPEA) in DMF at room temperature for 12 h, HATU and Et_3_N in THF at 0 °C to r. t. were employed by us [61]. Compound **3**, which was isolated as a white solid with a melting point value of 236–238 °C, was obtained as a colorless oil after FCC with a high level of purity by us. The structure of **3** and its high level of purity were confirmed by ATR-FTIR, ^1^H and ^13^C NMR spectroscopy, GC-MS, ESI-MS, elemental analysis and potentiometric titrations, which confirmed its molecular weight. Observing the ^1^H NMR spectrum of **3**, new signals were found, which are missing in the spectrum of its precursor UA, due to the introduction of the propargyl amine on C-28 carboxyl to form the propargyl amide function. Such signals included a triplet at 5.71 ppm (NH), a double doublet signal at 4.08 and 3.98 ppm with a *J* = 17.5 Hz (diastereotopic proton atoms of the methylene of propargyl group) and a triplet at 2.20 ppm was detected due to the ≡C−H group, whose multiplicity indicated couplings with the methylene (*J*^3^ = 2.5 Hz). Similarly, in the ^13^C NMR of **3**, new signals, due to the introduction of the propargyl amine were detected at 80.4 ppm (C≡C−H), 71.4 p (≡C−H) and 28.2 ppm (-CH_2_-C≡C−H), while the signal of C=O, which in the spectrum of UA was at 177.30 ppm [62], shifted at lower value of δ (176.1 ppm). Copies of spectra of **3** are available in Section S1.2 (Supplementary Materials) as Figures S1.2.8 and S1.2.9.

*(3β)-3-Hydroxy-3-(6-Triphenyl Phosphonium Hexanoate)-N-2-Propyn-1-Ylurs-20(29)-En-28-Amide* **4**. Compound **4** was unknown and was prepared for the first time by us, following the same procedure carried out for the esterification reactions of BET, using derivative **3**, having only one free hydroxyl to be esterified as the starting material, in place of BET, and **10** in place of 6-bromo hexanoic acid, as the acidic component. After isolation of pure **4** by FCC, it was obtained as a colorless oil in 34% yield. The structure of **4** and its high level of purity were confirmed by ATR-FTIR, ^1^H, ^13^C and ^31^P NMR, as well as by GC-MS, ESI-MS, elemental analysis and potentiometric titrations, which confirmed its molecular weight. In the ^1^H NMR spectrum of **4**, the signal of CH-3 shifted from the typical δ value reported for BA (3.19 ppm) [62] to 4.72 ppm. Five new methylene signals, due to the TPP-hexanoate alkyl chain, were dispersed in the region under 3.00 ppm. About these signals, those of CH_2_C=O (2.25 ppm) and of CH_2_P^+^ (3.90 ppm) groups were unequivocally assigned, while the signals of the 15 aromatic proton atoms of the TPP group were clearly visible at 7.85, 7.77 and 7.69 ppm. As in the proton spectrum, in the ^13^C NMR of **4**, the signal of C-3, which in the spectrum of BA is typically detectable at 76.80 ppm [62], was found at a higher value of δ (81.2 ppm), due to the esterification of the hydroxyl in C-3. The signal of C=ONH and C=OO groups were observed at 176.2 and 173.7 ppm, respectively, while those of three aromatic rings, sometimes in form of doublets, due to the coupling with P atom, were detected at 135.2 (3 equivalent CH=), 134.1, 134.0 (6 equivalent CH=), 130.8, 130.7 (6 equivalent CH=), 119.2, 118.6 (3 equivalent quaternary C), ppm. More signals were observed for the CH_2_P^+^ group at 22.9, 22.8, 228 and 22.6 ppm, due to the coupling with the P atom, while the CH_2_C=O group of hexanoate gave a signal at 34.4 ppm, as observed also in the spectrum of **6**. In the ^31^P NMR, a single signal was observed at *δ* = 24.39 ppm. Copies of spectra of **4** are available in Section S1.2 (Supplementary Materials) as Figures S1.2.10–S1.2.12.

#### 3.1.3. Synthesis of UA Derivatives

*(3β)-3-Hydroxy-N-2-Propyn-1-Ylurs-12-En-28-Amide* **13**. The UA derivative intermediate **13** was known and was previously prepared and characterized by different research groups [61,63,64]. Here, **13** was prepared avoiding the two-step procedure proposed by Xiao et al. in the years 2014 and 2016 [63,64]. In these papers, the authors prepared and isolated, first, the 1-benzotriazolyl triterpene ester by treating UA in THF with 1.5 equivalents excess of TBTU in the presence of DIPEA at room temperature, for 5 h under nitrogen, achieving the desired product after purification by FCC. Secondly, they transformed the 1-benzotriazolyl triterpene ester previously prepared, via its reaction with K_2_CO_3_ and 2-propargylamine in DMF at room temperature, being stirred for 1 h under a nitrogen atmosphere [63,64]. Our procedure was like the one-step synthesis proposed by Thi et al., with significant modifications. While authors reacted UA with DCC, HOBt and DIPEA in DMF, for 30 min. at room temperature, followed by the addition of propargyl bromide and stirring for 12 h at room temperature [61], we cooled a suspension of UA and treated it with propargylamine in THF, HBTU and Et_3_N, and stirred the mixture at -10 °C to r. t., upon completion of the reaction. As in other reported cases, while Thi et al. isolated **13** as a white solid with a melting point of 202–204 °C in 96% yield, we obtained a colorless oil in 22% yield (56% yield based on unreacted starting material). The structure of **13** and its high level of purity were confirmed by ^1^H and ^13^C NM spectroscopy. In particular, in the ^1^H NMR spectrum of **13**, new signals appeared with respect to the spectrum of UA, such as a triplet for the proton atom linked to the nitrogen of amide group (6.07 ppm), two complex signals of the diastereotopic protons of methylene of the propargyl group (4.03 and 3.89 ppm, *J* = 17.6 Hz) and the triplet of the proton atom of alkyne group, coupled with protons of methylene (2.20 ppm). In the ^13^C NMR spectrum of **13**, the signal of the C=ONH group shifted to a slightly lower value of chemical shift (178.2 ppm), with respect to that of C=OOH of UA, which is typically found at 178.7 ppm [65]. New signals appeared at 80.0 ppm (C≡C−H), 71.8 (≡C−H) and 28.4 (CH_2_-C≡C−H) ppm, due to the introduction of a new propargyl group. Copies of spectra of **13** are available in Section S1.2 (Supplementary Materials) as Figures S1.2.23 and S1.2.24.

*(3β)-3-Hydroxy-(6-Triphenyl Phosphonium-Hexanoate)-N-2-Propyn-1-Ylurs-12-En-28-Amide* **7**. Compound **7** was unknown and was prepared by reacting an ice-cooled suspension of intermediate **13** in DCM previously prepared, with a slight excess of **10**, in the presence of DCC and DMAP, following the usual esterification procedure carried out for preparing compounds **1**, **4**, **11** and **12**. After 24 h stirring at r. t., the reaction was completed, and the pure compound **7** was isolated as a colorless oil after FCC. The structure of 7 and its high level of purity were confirmed by ATR-FTIR, ^1^H, ^13^C and ^31^P NMR, as well as by GC-MS, ESI-MS, elemental analysis and potentiometric titrations, which confirmed its molecular weight. Particularly, in the ^1^H NMR spectrum of **7**, the signal of CH-3, which was reported at 3.01 ppm for UA [65] and was detected at 3.22 ppm in the spectrum of compound **13**, shifted to a higher value of δ (4.49 ppm). New signals appeared with respect to the spectrum of **13**, due to the introduction of the 6-TPP-exanoate in C-3. Specifically, signals for the 15 aromatic proton atoms of the triphenyl group were detected at 7.84, 7.78 and 7.69 ppm, while the signals of the protons of the five methylene groups of the alkyl chain were dispersed in the region where several other signals of the UA scaffold can also be found. The attribution of the signals of CH_2_C=O and CH_2_P^+^ groups was possible, with a high degree of certainty. In this regard, the methylene group linked to the P atom provided two multiplets at 3.88 and 3.82 ppm, while that linked to the C=OO group gave two signals at 2.26 (td) and 2.20 (t) ppm. In the ^13^C NMR spectrum of **7**, the signal of C-3, reported at 77.3 ppm for the UA [65] and found at 79.2 ppm in the spectrum of **13**, was detected at 79.9 ppm. New signals were observed for the C=OO group at 173.6 ppm, for the aromatic carbons of the TPP group at 135.2 (3 equivalent CH=), 134.0, 133.9 (6 equivalent CH=), 130.7 (6 equivalent CH=), 118.9 and 118.3 (3 equivalent quaternary C), some of which were in the form of doublets for the coupling with the phosphorous atom, and new signals were observed for the new methylene groups of the alkyl chain of the hexanoate. Of these signals, the signals of CH_2_C=O (34.3 and 34.2 ppm) and of CH_2_P^+^ (22.7 ppm) groups could be attributed with certainty. As for other TPP-containing compounds, the ^31^P NMR of **7** showed a single signal (*δ* = 24.38). Copies of spectra of **7** are available in Section S1.2 (Supplementary Materials) as Figures S1.2.25–S1.2.27.

### 3.2. ATR-FTIR Spectra of BA, BET, UA and Compounds ***1***–***7***

ATR-FTIR spectra of all samples were acquired as detailed in the experimental parts, and the related images are available in the Supplementary Materials in Figure S1.1.1–S1.1.10. Despite already being known, the ATR-FTIR spectra of BA, BET and UA were run anyway (Figure S1.1.1–S1.1.3) for a more reliable comparison with those of their derivatives by multivariate analysis as detailed in the Section 2 and later discussed in this section. Bands assignment to functional groups of the chemical structures of analyzed samples, reported in the Section 2, was made according to the Infrared Spectroscopy Absorption Table, available online at https://chem.libretexts.org/Ancillary_Materials/Reference/Reference_Tables/Spectroscopic_Reference_Tables/Infrared_Spectroscopy_Absorption_Table (accessed on 1 November 2025). Bands of stretching C-P were assigned according to what was reported by Daasch and Smith [66]. A detailed discussion of ATR-FTIR observed signals supported by reports from the literature [67] is available in the Supporting Materials after Figure S1.1.10 (Section S1).

### 3.3. Principal Component Analysis (PCA) of ATR-FTIR and ^13^C NMR Spectral Data

PCA is a chemometric tool that conducts part of multivariate analysis (MVA) and is able to reduce many variables of a certain dataset to a small number of new variables, called principal components (PCs) [68]. PCA allows us to reduce a high number of correlated variables (organized in a matrix of n columns x n rows), also including those variables providing non-significant and redundant information, to a limited number of uncorrelated variables, namely principal components (PCs), providing the most important information [69]. The number of PCs is always lower than the number of samples analyzed. In the following sections, we have presented and discussed the results obtained by processing both ATR-FTIR and ^13^C NMR spectroscopic data by PCA, presented as score plots. In both cases, in the score plot, the scores were the new coordinates of the processed samples in the new space of the PCs. Note that PCA was applied to process spectral data intended as transmittance (%) (ART-FTIT) or δ (ppm) values (^13^C NMR), which are correlated to the functional groups present in the samples’ structures and with their structures themselves, in the first case, and to the number and type of carbon atoms present in the samples’ structures, and with their structures themselves, in the second case. In this regard, within the score plot, each sample assumed a position depending on its chemical composition and structure [70]. Samples located close to each other shared similar characteristics, while those placed far apart differed [70].

#### 3.3.1. PCA of ATR-FTIR Spectral Data

**Figure 3:**
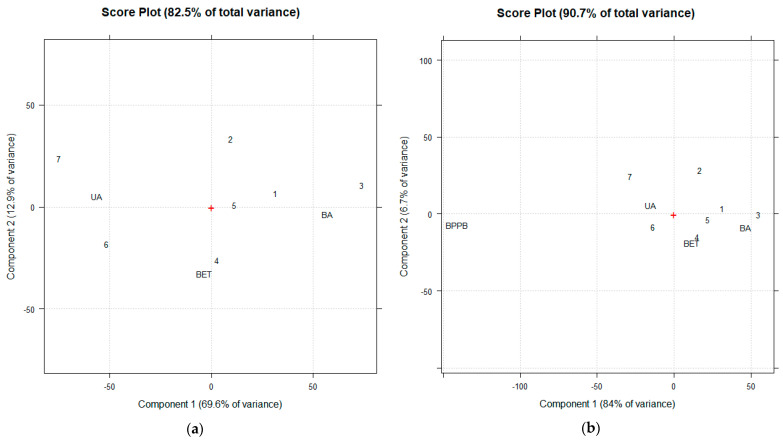
Score plot of PCA on the matrix of 34,010 variables (spectral data of BA, BET, UA and compounds **1**–**7** (**a**) and that of PCA on the matrix of 37,411 variables (spectral data of BA, BET, UA, compounds **1**–**7** and BPPB (**b**). The red cruise indicates the interaction of the two principal components considered (PC1 and PC2) at point 0.

The matrix of 34,010 variables (A) and that of 37,411 variables (B), where variables were the transmittance (%) values of each sample in the ATR-FTIR spectrum, were constructed as described in the Section 2. The results from the PCA on both A and B have been reported as score plots explaining a total variance of 82.5% and 90.7%, respectively. In the first case, PC1 explained 69.6% of total variance vs. PC2, which explained 12.9% (Figure 3a), while in the second case, PC1 explained 84.0% of total variance vs. PC2, which explained 6.7% (Figure 3b).

As observable in Figure 3a, BA and UA, which sheared the carboxylic group, despite being in opposite locations on PC1 (negative score for BA and positive for UA), due to the significantly different triterpene structure, assumed similar scores close to 0 on PC2. Conversely, BET not bearing the carboxylic group was located distant from either BA and UA, both on PC1 (close to 0 score) and PC2 (negative score). Shearing the same triterpene structure as BA, BET was positioned closer to BA than to UA on PC1 and had a negative score on PC2, as BA, while UA had a positive score. The PC unequivocally evidenced that compounds **3** and **7** are BA and UA derivatives, respectively, since on both CP1 and CP2, they have scores very similar to those of BA and UA. As UA, **7** had negative scores on PC1 and positive scores on PC2, while as BA, **3** had positive scores on PC1 and close to zero on PC2. On PC1, compounds **1**, **2**, **5** and **4** were positioned between BET and BA, since they sheared with them the same triterpene structure, while they were positioned at opposite scores (positive) with respect to UA (negative) due to its significantly different triterpene structure. While BA was positioned at a score > 50 as **3**, compounds **1**, **2** and **5** were at scores < 50, closer to BET, thus confirming their BET origin. Additionally, compounds **1** and **5** were very close to score 0 and to each other on PC2 since, differently from **2**, they contained the TPP-hexyl ester group. The BET origin of **6** was evident on PC2, since both BET and **6** occupied similar negative scores. The fact that compound **6** was positioned at negative scores > −50 on PC1, as compound **7**, could depend on their double functionalization in C-3 and C-28 with the same groups, while the presence of a different triterpene nucleus in such compounds was evidenced on PC2, where **7** had a positive score, and **6** had a negative score. Moreover, despite its double functionalization, compound **1** was located a significant distance from both **6** and **7**, with a positive score on PC1, and close to zero on PC2 because of the presence of a carbamate functional group absent in both **6** and **7**. The initially unexpected location of compound **4**, very close to BET, insteat that to BA, which is its parentcan be explained with the same triterpene core for both BET and BA, which were retained similar and not distintcted on both PC1 and PC2. It is more discriminant the double functionalization of **4** on both C-3 and C-28 as **6** which caused its location at scores similar to those of **6** on both PC1 and PC2. Notably, adding to the dataset the spectral data of BPPB, which was sheared with compounds **1**, **4**, **5**, **6** and **7,** only the presence of the TPP group, the PCA score plot of PC1 vs. PC2, well-separated BPPB from all other triterpene compounds on PC1. While BPPB was located at a negative score > −100, all other compounds were at positive scores, or, in the case of UA, **7** and **6**, they were located at negative scores, but very close to zero. Curiously, the relative positions occupied by the triterpene compounds were identical to those occupied by them in the entire score plot in Figure 3a, except that in this new score plot, they grouped on its right side to distance themselves from BPPB. A greater similarity between compound **6** and BPPB, due to the presence of two TPP groups, was evidenced on PC2, where compounds had practically identical negative scores.

#### 3.3.2. PCA of ^13^C NMR Spectral Data

**Figure 4:**
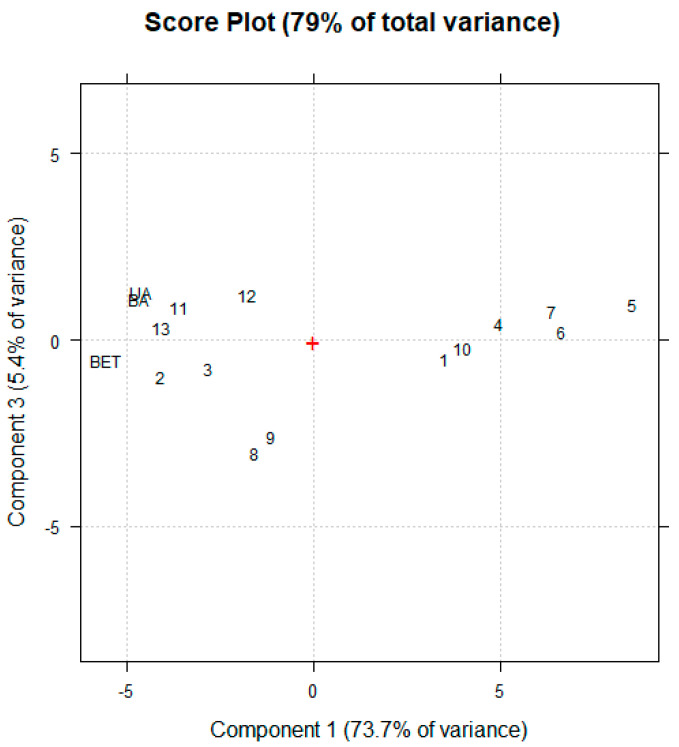
Score plot of PCA on the matrix of 510 variables consisting of the values of δ (ppm) of the ^13^C NMR spectral data of BA, BET, UA, compounds **1**–**7** and intermediates **8**, **9**, **10**, **11** and **12**. The red cruise indicates the interaction of the two principal components considered (PC1 and PC2) at point 0.

To perform PCA on a dataset of NMR spectral data, ^13^C NMR data were chosen in place of ^1^H NMR ones, because total-decouple carbon NMR provides, in most cases, a single peak for values of δ (ppm), thus avoiding multiplicity. The matrix of 510 variables, where variables were the values of δ (ppm) of signals in a selected region of the ^13^C NMR spectrum of each analyzed sample, was constructed as described in the Section 2. The results from PCA have been reported as score plots explaining a total variance of 79%. In the score plot, PC1, explaining 73.7% of total variance, has been reported against PC3, explaining 5.4 of total variance (Figure 4).

As observable in Figure 4, the three pristine triterpenoids were located very close to each other at the most negative scores of about −5 on PC1, thus evidencing their differences from their derivatives. Since BA and UA sheared the carboxylic group, while BET did not, they were very close to each other on PC3 at positive scores, while BET was positioned at a negative score. Moreover, all samples bearing the 6-triphenyl phosphonium hexanoyl group (**1**, **4**, **5**, **6** and **7**) were well separated from compounds not bearing it (BET, BA, UA, **2**, **3**, **11**, **12**, **8** and **9**) on PC1. Precisely, they located all at positive scores, while the compound without such a group positioned them all at negative scores. Among the TPP group at positive scores, apart from compound **1**, which is located curiously close to **10**, PC1 well-separated compounds having the triterpene nucleus, from the non-triterpene **10**. Anyway, the clear separation of **1** from **4**, **5**, **6** and **7** on both PC1 and PC3 could indicate that **1** is the only TPP-compound containing the carbamate group. Among the non-TPP-compounds at negative scores on PC1, PC3 well-separated the majority of compounds containing the triple carbon–carbon bond (**2**, **3** and **9**), which were located at negative scores from those not owing it (**11** and **12**), instead positioned at positive scores. As exceptions, despite containing the triple bond, **13** was located at a positive score, because of its structural identity with UA, which was its origin, while **8** was located at negative scores, because of its structural identity with BET, which was its origin.

### 3.4. Optical Microscopy Analyses

**Figure 5:**
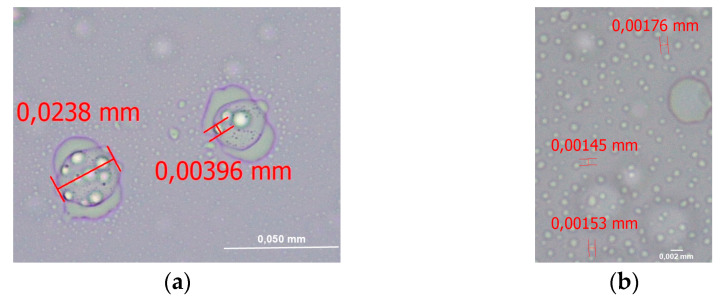
Vesicular aggregates of **6** in water solution observed with a 40× objective (**a**). Smaller spherical vesicles of **6** were better evidenced using the 100× objective (**b**). In these images, commas have been used by the instrument software for decimal separation.

It has been reported that compounds bearing two triphenyl phosphonium groups (TPP) linked by a hydrophobic chain of 12 carbon atoms, defined as bola-amphiphiles, when dispersed in aqueous solution at proper concentration, spontaneously self-assembled into spherical vesicles. Depending on the objective used (40× or 100×), vesicles appeared as spherical aggregates (10–30 µm) of smaller spheres (1–3 µm) or well-dispersed small vesicles of 1–3 µm [8,71]. In this regard, leaving apart compounds **2** and **3**, not possessing the TPP groups, water solutions prepared according to the procedure detailed in Section 2 of all compounds bearing TPP, were investigated using optical microscopy. The solutions were examined with a 40× and 100× objective, observing spherical polydispersed vesicles only for compound **6** carrying two TPP groups, linked to the hydrophobic structure of betulin (BET) via spacers of 7 and 8 atoms. This finding confirmed that the capability to spontaneously form vesicles in water is particular to bola-amphiphilic compounds [71]. Figure 5 shows the spherical vesicles provided by **6** as they appear when observed with the 40× (Figure 5a) and 100× (Figure 5b) objectives.

At the used concentration of **6**, large spherical vesicles of 24 μm or larger were clearly visible using a 40× objective. As previously reported by us for a much simpler bola amphiphilic compound (BPPB), encompassing two TPP groups as **6**, but linked by a less complex spacer, made of a chain of 12 carbon atoms [8], these spheres revealed to be formed of smaller vesicles of about 4 μm, which also appeared not clearly visible in the background (Figure 5a). Using the 100× objective, it was possible to bring to the forefront these smaller vesicles of 1.58 ± 0.13 μm, leaving on the background the larger aggregates (Figure 5b). Despite optical microscopy not being a precise method to evaluate the morphological and topographical characteristics of particles, as well as their size, because observations are strictly limited by the focus distance, these findings confirmed that compounds bearing two cationic heads linked by hydrophobic spacers form spherical vesicles of different sizes in water, which can aggregate in larger one depending on the solution concentration and coexist with them [8]. As reported, **6** was able to self-assemble in spherical vesicles, due to its planar cationic TPP headgroups, because self-assembly properties are strongly dependent on the complex interplay of non-covalent interactions (ionic, hydrophobic, and π-π) inside the aggregate [8]. In this regard, the π-π stacking between the three aromatic rings on the TPP heads of **6** was crucial for the final aggregate morphology.

### 3.5. Particle Size, ζ-p, and PDI of ***6***

**Figure 6:**
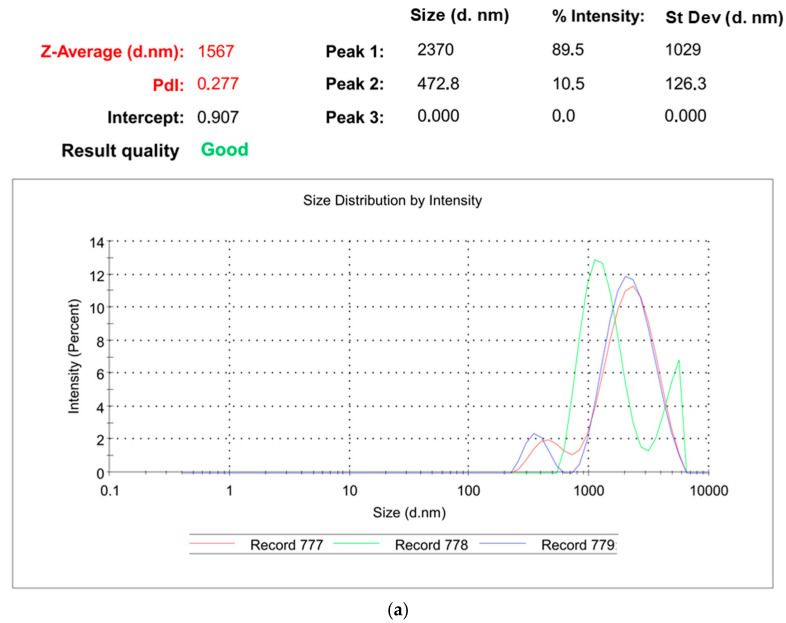

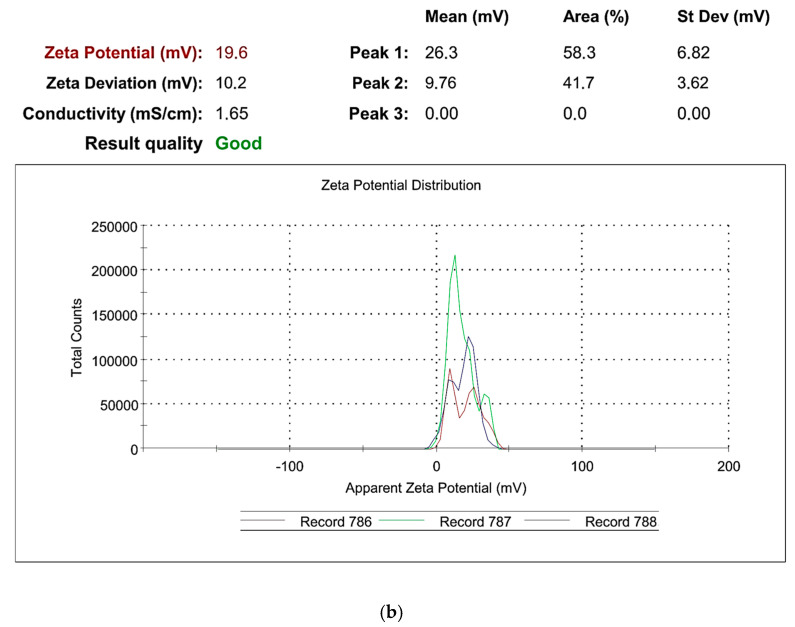
Representative images of the particle size distribution (nm) with DPI of 6 (**a**) and of its ζ-p (**b**).

Using the m-Q water solution of **6** (5 mM), the hydrodynamic size (diameter) (Z-AVE) and polydispersity index (PDI) of **6** vesicles were determined by DLS analysis to assess the dimensional distribution of their particles and how much of their distribution could be uniform. Additionally, zeta potential (ζ-p) measurements were carried out on the same solution to determine its surface charge. According to the results reported in Figure 6a, **6** the analysis evidenced the presence of more than one-dimensional family. Precisely, small-dimensional families made of nanovesicles were detected with an average size of 473 nm, together with larger families made of microparticles up to 2.4 µm. Collectively, the calculated average vesicle dimension was 1.6 µm, while PDI was 0.277, thus confirming the scenario already observed in OM.

Different from what was observed in our recent publication, where the microparticles observed for BPPB in OM analysis, as for **6**, at the DLS investigation appeared nano-dimensioned (49 nm), particles formed by **6** in water resulted micro-dimensioned also in DLS analysis. We suppose that the fact that vesicles from **6** were micro- and not nano-dimensioned could depend on the nature of the linker, which was more complex than the simple carbon chain of BPPB, also encompassing atoms different from carbon and mainly the tri-dimensional structure of BET, which influenced the final dimensions of vesicular spheres. As evidenced in Figure 6b, vesicles self-formed by **6** in aqueous solution demonstrated various ζ-p distributions, as in the case of size Collectively, the average ζ-p of **6** was positive and equal to 19.6 ± 10.2 mV. The size of particles strongly influences their distribution, cytotoxicity, and targeting ability when in vivo administered [24,72,73]. Generally, in biomedical applications, compounds for systemic administration should require sizes smaller than 200 nm, with an optimal size of 100–200 nm [24]. In this regard, envisioning a possible future clinical administration of **6** as an antibacterial agent, due to the micrometric size of its particles, it could be suggestable for topical administration to treat skin infections. Concerning ζ-p, **6** showed positive values, which are desirable for effective drugs. In fact, based on the studies published so far, the internalization of positively charged nanoparticles (NPs) is more efficient than that of neutral and anionic ones [74,75,76,77,78,79]. It was in fact discovered that after electrostatic interaction with anionic components of the cell membrane as phospholipids, positively charged materials can enter the cells by both pore formation, micropinocytosis and clathrin- or dynamin-dependent endocytosis [75].

### 3.6. Non-Aqueous Potentiometric Titration of Compounds ***1*** and ***4***–***7***

To have further confirmation of the molecular weight (MW) and structure of cationic compounds **1** and **4**–**7**, we titrated their phosphonium groups to experimentally determine the P^+^ equivalents contained in an exactly weighted sample. Comparing the obtained results with those calculated according to the molecular weight of **1** and **4**–**7**, we would have had confirmation of their mass and structure. In this regard, non-aqueous titrations emerged in the middle of the last century, enabling the possibility of titrating both weak acids and bases, not measurable in aqueous media [80,81,82]. In particular, the titration with perchloric acid in glacial acetic acid medium is widely used to determine the salt of weak basic drugs. Titrations in acetic anhydride–acetic acid mixture allow the direct non-aqueous titration of halide salts (mainly hydrochlorides) of organic bases and quaternary ammonium salts. Additionally, by adding mercury (II) acetate reagent to the quaternary ammonium salts solution, stable mercury (II) halide complexes and free acetate ions (equivalent to the base) form, which can be titrated with perchloric acid [28,83]. On these considerations, we carried out the potentiometric titration of quaternary phosphonium salts **1** and **4**–**7** in a mixture of anhydrous acetic acid (AcOH) and acetic anhydride (Ac_2_O) 30:70 (*v*:*v*) with 0.1 N HClO_4_, performing the slightly modified procedure previously described by us for the volumetric titration of ammonium salts and described in the Section 2. Briefly, we adapted the protocol described by Pifer and Wollish, who applied this method for titrating quaternary ammonium salts [28] and used micropipettes and micro-burettes for measuring volumes. By plotting the measured mV values against the volumes of 0.1 N HClO_4_ solution added, we obtained the titration curves of all compounds and the related first derivative (FD) curves. The maxima of the FD represent the titration end points, which allowed us to find the volumes of titrating solution needed to titrate the phosphonium groups of our samples and then their P^+^ equivalents. Table 2 reports the experimental details of titrations, the calculated P^+^ equivalents for all samples according to their molecular weight (MW), the experimentally determined P^+^ equivalents obtained by titrations, the experimental MW, the residuals and the percentage error (%).

The experimental MWs were perfectly in agreement with the calculated ones, with a maximum error (%) of 0.78% (<1%), thus further confirming the structure of **1** and **4**–**7**.

### 3.7. Antibacterial Properties

With screening purposes, the antibacterial properties of the synthesized triterpenoid derivatives **1**–**7** were assessed by determining their minimum inhibitory concentration (MICs) against selected clinical isolates of both Gram-positive and Gram-negative species. Additionally, the antibacterial effects of not modified triterpenoids betulin (BET), betulinic acid (BA) and ursolic acid (UA) used to prepare compounds **1**–**7**, were also investigated for comparison purposes. For MICs evaluation, the seven MDR strains described in Section 2.8.1 and reported in Table 1 were used. We selected these superbugs since, currently, a large variety of mechanisms of resistance have been observed, which bacteria could develop to inactivate available antibiotics and make it difficult to manage infections sustained by mutated bacteria. Such mechanisms include the production of potent β-lactamases, such as New Delhi Metallo beta-lactamase (NDM), *Klebsiella pneumoniae* carbapenemase (KPC), β-lactamases enzymes of AmpC type, conferring resistance to ceftazidime-avibactam, D-family β-lactamases OXA-427 and *Pseudomonas* extended resistant (PER)- and sulfhydryl variable (SHV)-type extended spectrum β-lactamases (ESBLs) [7]. Other mechanisms consist of porin mutations and mutations affecting siderophore receptors, efflux pumps, and target (PBP-3) modifications [7]. Therefore, to improve this condition, limit the emergence of further resistance and counteract the spread of worrying infections, novel compounds that also function on superbugs, as those tested in this study, are urgently needed. All bacteria used in this study were clinical isolates that had developed resistance to at least one or two antibiotics. In particular, MDR *E. faecium,* as well as *S. aureus*, as those used here are included by WHO among the ESKAPE bacteria. ESKAPE strains are a group of Gram-positive and Gram-negative bacteria capable of evading or ‘escaping’ commonly used antibiotics, due to their increasing multidrug resistance (MDR) [84]. As a result, they are the major cause of life-threatening opportunistic and hospital-acquired infections throughout the world [85]. Table S1 in Section S2 of the Supplementary Materials (SM) reports all observed MICs for compounds **1**–**7**, BET, BA and UA on both Gram-positive and Gram-negative species tested, as well as those of reference antibiotics currently available to counteract the considered clinical isolates. Conversely, the following Table 3 is a refined form of Table S1, where data concerning Gram-negative species, and not active compounds **2** and **3**, were not further reported.

It is noteworthy that, despite several articles in the literature consider new compounds as active against bacteria also when exert MICs over 128 µg/mL, some authors have reported that only compounds with MICs ≤ 32 µg/mL can be retained active [86]. In this regard, as a compromise between these two trends, here, only compounds with MICs ≤ 64 µg/mL were considered endowed with a certain antibacterial effect. According to these guidelines, all compounds tested in this study were inactive against Gram-negative species (MIC > 64 µg/mL). Six out of ten compounds displayed from weak (compound **1** and UA (staphylococci), MICs = 32–64 µg/mL) to strong antibacterial effects (compounds **4**–**7** and UA (enterococci), MICs = 2–16 µg/mL), against the here-considered Gram-positive strains. These observations align with the fundamental challenges associated with discovering compounds with activity against Gram-negative bacteria [87]. In fact, the attenuated activity of potential antimicrobial agents against *E. coli*, *K. pneumoniae*, *P. aeruginosa* and other bacteria representative of Gram-negative species can be related to the reduced permeability of their outer membrane, often acting as a barrier to the entry of antimicrobial drugs or drug candidates [88]. Conversely, the cell wall of Gram-positive bacteria makes the permeation of these compounds easier, allowing them to enter the bacterial cell [88].

However, it has also been reported that strongly cationic compounds and macromolecules can exhibit antibacterial effects against Gram-negative species, comparable or superior to those observed on Gram-positive isolates. Such cationic compounds include antimicrobial cationic peptides (CAMP) such as colistin [89,90], dendrimers with a high number of peripheral ammonium groups [91], cationic benzyl ammonium-based co-polymers [24,92,93], quaternary phosphonium and ammonium polymers [90] and bola-amphiphilic bis-phosphonium nanovesicles [8]. These molecules act not specifically as membrane disruptors on contact, without the need to enter cells.

Colistin is a classic example of an antibacterial drug that is active only on Gram-negative bacteria and not on Gram-positive ones, since it is less attracted to the surface of the latter [94]. In fact, the antibacterial potency of strongly cationic materials is mainly due to their greater affinity with the membrane of Gram-negative bacteria than with that of Gram-positive ones, where the anionic character is weaker [89,90,91].

Additionally, it has been demonstrated that compounds bearing TPP groups linked to hydrophobic carbon chains of different lengths can exhibit very low MICs = 0.25–8 [86] and 1–32 [8] µg/mL also against *E. coli*, *K. pneumoniae* and *P. aeruginosa*, regardless of their complicated pattern of resistance [8]. Such compounds are very promising as new antibacterial devices to counteract the difficult-to-treat infections sustained by these Gram-negative species.

#### 3.7.1. The Rationale of the Inactivity of the TPP-Bearing Compound of This Study Against Gram-Negative Isolates

According to MICs > 1024, equal to 256–512 and >128 µg/mL, already reported for BET [95], BA [96], and UA [97], against Gram-negative species, it was expected that such pristine triterpenoids could be inactive against *E coli, P. aeruginosa* and *K. pneumoniae* tested in this paper. Anyway, according to data from the literature [8,86], it was unexpected that their synthetic derivatives containing the TPP group could be ineffective as well. A compound recently reported bearing two TPP groups linked by a C12 alkyl chain (BPPB in the study), despite being inferior to those displayed against Gram-positive isolates, demonstrated potent antibacterial effects, as confirmed by very low MICs = 1–2 µg/mL on *E coli*, 16–32 µg/mL on *P. aeruginosa* and 16 µg/mL on *K. pneumoniae* [8]. Nunes et al. evidenced that, among a series of quaternary ammonium and phosphonium salts, bearing different cationic heads and carbon chains, those bearing the TPP group demonstrated the most effective antimicrobial activity and the broadest antimicrobial spectrum, providing in the best cases MICs low to 1 (*E. coli*) and 8 (*P. aeruginosa* and *K. pneumoniae*) µg/mL [86]. Nonetheless, in another less recent study by us [30], a differently structured TPP-bearing compound (PPB), having a 11-undecanol chain linked to a single TPP group, was active against Gram-positive strains including staphylococci and enterococci with low MICs = 4–16 µg/mL, while it was considered inactive against Gram-negative isolates (>128 µg/mL) [30], like TPP derivatives **1** and **4**–**7** of this study.

To explain this fact, it must be considered that the antibacterial efficacy of quaternary cationic salts (QCSs) is intricately linked to their physicochemical properties, including hydrophobic–hydrophilic balance, water solubility, adsorption efficiency, and critical micelle concentration, which could be very different in different TPP-containing compounds [26,27,98]. Generally, the possible antibacterial effects of TPP containing QCSs depend mostly on their capacity to establish electrostatic interactions with the negatively charged bacterial cell surfaces [98]. Collectively, the presence of the permanently cationic TPP group and its capability to create also hydrophobic interaction with bacterial membranes, due to the π-π system of its phenyl rings, play a pivotal role in the effective antibacterial activity of TPP-containing compounds [8,30,98]. Practically, upon early electrostatic interactions with the bacterial envelope, promoted by the cationic character of TPP, such compounds can also readily penetrate the protein–lipid biological membranes of bacteria, thus compromising their structural integrity and functions [2,3,28]. This leads to further detrimental events, such as bacterial protein denaturation, nucleoprotein complex disruption, etc., ultimately resulting in irreversible and lethal damage to the pathogenic cells [2,3,28].

It is thus derived that the “weight” that the TPP group assumes within the whole molecule that contains it becomes of extreme importance for the molecule’s antibacterial effects and for its spectrum of activity against bacteria. Especially, for efficacious effects against Gram-negative species, having a more negative and difficult to penetrate outer membrane, stronger π-π and electrostatic interactions, which can be promoted by more than one cationic TPP group, are needed and could translate into higher membrane damage, causing cell death. Therefore, among previously reported BPPB and PPB, the latter, containing only one TPP residue and possessing a more hydrophilic OH-terminated shorter carbon chain and probably unable to self-assemble nanovesicles in water, was not active against Gram-negative isolates, while strongly active against Gram-positive strains [30]. Conversely, BPPB, owning two TPP heads, linked by a simple and hydrophobic C12 chain and giving nanovesicles of 49 nm in water, was active against both species [8]. Collectively, the structural differences between BPPB and PPB led PPB to have a lower capability to interact with the strongly negative surface of Gram-negative bacteria and kill them on contact, thus resulting in being active on these species [30]. At the same time, the *bis*-cationic structure of BPPB enabled it to be significantly more active than PPB against Gram-positive isolates (0.25–0.50 vs. 4–16 µg/mL) and to be active also against *E coli*, *P. aeruginosa* and *K. pneumoniae* with MICs = 1–32 µg/mL) [8].

Translating these considerations to compounds **1**, **4**, **5** and **7** of this study, they contained only a TPP group as PPB, did not provide nanovesicles in solution and were constructed on original triterpenoids (BET and BA) known to be inactive against Gram-negative species (MICs from >128 to >1024 µg/mL). All these factors contributed to making them inactive against *E coli*, *P. aeruginosa* and *K. pneumoniae* (MICs > 64 µg/mL). The BET derivative **6**, despite possessing two TPP groups as BPPB and providing spherical aggregates in water, was constructed on a BET skeleton, which is reported as the triterpenoid with the lower antibacterial effects among those considered here and the highest MICs against both Gram-positive and negative species (MICs > 1024 µg/mL) [95]. Additionally, the aggregates formed in water by **6** were micro-dimensioned, thus not possessing the high penetrating capacity of nanovesicles (49 nm) formed by BPPB, thus not reaching its antibacterial potency and failing against Gram-negative isolates (MICs > 64 µg/mL).

#### 3.7.2. Antibacterial Behavior of BET, BA, UA and Compounds **1**–**7** Against Gram-Positive Isolates

Pristine BET and BA were inactive also against Gram-positive strains, as well as their derivatives **2** and **3** (MICs > 64 µg/mL), not containing the TPP group, thus confirming reports from the literature where BET demonstrated MICs > 1024 µg/mL against *S. aureus* [95] and BA showed MICs = 256 µg/mL against *S. aureus* and *S. epidermidis* [96]. Collectively, despite the various pharmacological properties of BET and BA, which make them promising compounds as multitarget agents [99,100], they are not encouraging as novel antibacterial agents, as well as their derivatives **2** and **3**, thus not meeting our research needs. In this regard, the overall merit of this study consists of having found the correct approach to recovering these compounds for possible use as future antibiotics, conferring them from weak (**1**) to strong antibacterial effects (**4**–**6**) at least against Gram-positive superbugs, including ESKAPE bacteria, via insertion of the TPP moiety.

According to what was reported by us concerning the antibacterial effects of UA on the same strains used in this study, using the EUCAST-approved cation-adjusted Muller–Hinton broth, it possesses per se week antibacterial activity on both staphylococci isolates (MICs = 32–64 µg/mL) and potent antibacterial effects on enterococci (MICs = 2–4 µg/mL) [97]. Our results were in accordance with those of Farjardo et al. concerning *S. aureus* (31.3 μg/mL), while MICs observed by us concerning *E. faecalis* were lower than those of Farjardo et al. by about 8 times [101]. On the contrary, Sun et al. found MICs inferior to ours by 8 times (MICs = 8 µg/mL), 16 times (MICs = 4 µg/mL) and 4 times (MICs = 16 µg/mL) on *S. aureus* (MRSA), *S. aureus* ATCC 6538 and *S. aureus* ATCC 29,213, respectively, and inferior to ours by 16 times on *S. epidermidis* ATCC 12,228 (MICs = 2 µg/mL) [102]. Also, Do Nascimento et al. reported for UA, MICs like lower by 2 times (MICs = 32 µg/mL) when tested on *S. aureus* [103]. Additionally, different MICs lower than those observed by us for MRSA and VRE by 21, 8 and 8 times were reported in the past by Volska et al. for UA (MICs = 3, 4, 8 µg/mL), when tested on MRSA, VRE and *S. aureus* ATCC 25923, respectively [104]. This scenario unequivocally evidences that data from the literature concerning the antibacterial effects on Gram-positive species of UA are strongly contrasting, depending on the UA origin and the broth used.

Specifically, compound **1** was constructed on a BET core by inserting a propyne carbamate residue on C-28 and a TPP-bearing group on C-3. It was weakly active against both staphylococci and enterococci, exhibiting MICs in the range of 32–64 µg/mL, which is at our threshold of inactivity, despite the presence of the TPP group. A slightly higher antibacterial activity was observed on *S. epidermidis* and *E. faecium* (MICs = 32 µg/mL), rather than on *S. aureus* and *E. faecalis* (MICs = 64 µg/mL). Notably, these isolates demonstrated to be the superbugs most difficult to inhibit, except for compounds **4**, **6** and **7,** which were equally strongly active against *S. aureus* and *S. epidermidis*. Although, in our opinion, the antibacterial effects of **1** could be considered, in general, to be poor, they were significantly higher than those of 9 out of 12 BET derivatives reported as promising antibacterial devices and synthesized by Deng et al. for conferring BET a certain antibacterial effect [95]. Specifically, MICs of **1** against MRSA were lower than those of different BET-derived thioether and S-alkylated sulfonium BET derivatives, by 2–387 times [95]. MICs of **1** on MRSA were 4 times lower than those reported by Serrafi et al. for 6 BET derivatives when tested on *S. aureus* ATCC 25,923 [105]. Moreover, **1** was a better performant in 4 out of 5 BET derivatives synthesized by Haque et al., screened by a wider library of 51 BET-derived compounds [106]. Specifically, MICs of **1** against superbugs of the *S. aureus* and *E. faecalis* genus were lower than those of 4 compounds synthesized by Haque et al. by 1.6 times [106].

When the BET-derived core of **1** was replaced with the BA-derived core, to give **4** analogously functionalized with the same TPP-bearing group of **1** on C-3 and having a propyne amide group on C-28, the antibacterial activity resulted drastically enhanced. Compound **4** exhibited MIC = 4 µg/mL on all strains, except for potent *E. faecalis*, where the observed MIC was 16 µg/mL. Practically, by replacing the BET nucleus with that of BA, which intrinsically has MICs decidedly lower than those of BET [96], the MICs of **1** were lowered by 2–16 times, thus achieving a compound promising for further experimentation as an antimicrobial agent. Compound **4** demonstrated antibacterial activity against MRSA extraordinarily higher than that of two novel BA semisynthetic derivatives ((17 S)-17-(((dimethoxyphosphoryl)methoxy)carbonyl)-3β-hydroxy-28-norlup-20(29)-ene as BPm and sodium (3β-hydroxy-(17R)-17–28-norlup-20(29)-en)-2-oxoethyl-phosphonate as BP), which were synthesized and characterized by Lugiņina et al. [107]. In particular, **4** was more potent of the two BA derivatives by 136 times, despite such compounds being tested on *S. aureus* strains without a defined pattern of resistance [108].

When the unmodified BET nucleus was functionalized by esterification reaction of the hydroxyl on C-28 with the usual TPP-bearing moiety, in place of using the propyne derivative previously inserted and leaving free the hydroxyl on C-3, compound **5** was achieved. Curiously, despite the BET nucleus, previously considered the most responsible for the weak antibacterial effects of **1**, **5** displayed antibacterial effects equal to those of **4** on *S. epidermidis* (MIC = 4 µg/mL), higher on *E. faecium* (MIC = 2 µg/mL) and *E. faecalis* (MIC = 8 µg/mL), and slightly lower than those of **4** on *S. aureus* (MIC = 8 µg/mL). These findings evidenced important structural characteristics that are to be considered to have triterpenoid derivatives based on BET or BA with strong antibacterial effects, at least on Gram-positive species. The presence of propyne-containing groups on C-28 could be dangerous rather than advantageous. Such a modification has, in fact, left unchanged the antibacterial inactivity of BET and BA in the absence of the TPP group. Additionally, although despite such modification, the further insertion of the TPP group in C-3 on the BA derivative **3,** led to achieve **4**, which exhibited potent antibacterial effects, the same insertion on the BET derivative **2** did not translate in similar outcomes but provided **1**, possessing only weak antibacterial effects. Collectively, in BET derivatives, propyne moieties on C-28 are not suggestable, both in the presence and absence of the TPP group. Also, the TPP group should be inserted preferably, by esterification reaction of the hydroxyl on C-28 rather than of that on C-3, which should be left free. This was confirmed by compound **6**, where the further hydroxyl esterification on C-3, despite introducing an additional TPP cationic group, has lowered the antibacterial effects of **5** by two times rather than enhancing them. Ultimately, both compounds **5** and **6** outperformed the antibacterial efficiency of all BET derivatives synthesized by Deng and Serrafy et al. [95,105], and of 4 out of 5 BET-derived compounds proposed by Haque et al., by 14 times against *S. aureus* (5 and 6) and by 7 times on *E. faecalis* [106].

The best-performant compound was **7**, as could be expected, since it was constructed using the natural scaffold UA, which was already endowed with antibacterial activity against Gram-positive strains, especially of the *Enterococcus* genus [97]. Although the presence on C-28 of the propyne carbamate derivative, in place of the TPP-containing moiety, which was instead inserted on the hydroxyl on C-3 as in compounds **1** and **4**, compound **7** revealed strongly enhanced antibacterial effects respect to pristine UA against staphylococci, where it displayed the lowest MICs (2 µg/mL), thus reducing those of UA, by 16–32 times. Concerning enterococci, the antibacterial effects did not change. Therefore, it is rational to think that the insertion of the TPP-containing group on the hydroxyl on C-28 in place of the propyne derivative, leaving free the hydroxyl on C-3, could provide a better-performant compound with lower MICs also on enterococci. Comparing the results of this study with those obtained by us encapsulating UA in G4K nanoparticles, which demonstrated MICs > 128 µg/mL against both staphylococci species tested here, 64 µg/mL against *E. faecalis* and 32 µg/mL against *E. faecium*, **7** demonstrated MICs lower by 16 to much more than 64 times [97]. On the other hand, compared to cyclodextrin β-UA (UA-CDβ) nanocomposites proposed by Farjardo et al. as macromolecules with improved antibacterial effects with respect to pristine UA, **7** outperformed their activity by 2 times on *E. faecalis* and by 4 times on *S. aureus* [101]. Also, compared to the antibacterial activities of UA derivatives synthesized recently by Sun et al., when tested on *S. aureus* (MRSA), **7** outperformed 33 out of 50 compounds and provided results equal to those reported by Sun et al. for 11 compounds among the prepared ones, as well as **7**, were more active than norfloxacin by 2 times [102]. Additionally, when tested by Sun et al. on *S. epidermidis* (MRSE), **7** was more performant than 31 out of 50 compounds and equally performant to 13 compounds and norfloxacin, which were instead tested on *S. epidermidis* ATCC 12,228 [102]. Also, none of the 38 UA and oleanolic acid (OA) derivatives synthesized by Wu et al. exerted antibacterial activity at least comparable with that of 7 when they were tested on ATCC strains of *S. aureus*, *S. epidermidis* and MRSA [109]. Specifically, **7** was more potent than compounds tested by Wu et al. by 1.6–12.5 times, as well as when it was tested on MRSE rather than on ATCC *S. epidermidis*, as carried out by Wu et al. On MRSA, **7** was more performant than all compounds synthesized by Wu et al. by 2.5–50 times [109]. Despite these results demonstrating that none of these new compounds was more potent than BPPB, as recently reported by us [8], at least compounds **4**, **5**, **6** and **7** outperformed most of the naturally derived antibacterial agents reported so far, especially considering the complex pattern of resistance of bacteria selected by us and their clinical origin.

Collectively, it can be assumed that our TPP-bearing compounds behave as antimicrobial agents irrespective of the difficult pattern of resistance of bacterial isolates tested, mainly by targeting the envelope of bacteria, as reported in [110]. In fact, the interaction of TPP-conjugates with the bacterial cell membrane played a key role in their mechanism of action. This involves interaction with the bacterial cell membrane, causing destabilization of membrane proteins and lipids, which disrupts normal cellular processes and affects the metabolism of the bacterial cell wall, leading to disruption of cell division [110]. For the first time, we have succeeded in conferring real (MICs = 2–16 µg/mL) antibacterial activity to BET and BA, thus recovering these natural compounds, already endowed with several pharmacological properties, which make them possible multi-target drugs, also for possible antibacterial uses against worrying Gram-positive ESKAPE bacteria. Moreover, despite BPPB revealing acceptable toxicity on several cell lines [8,111,112,113,114], we thought that it could be possible that the insertion of similarly structured groups on non-cytotoxic natural scaffolds could have provided compounds with further enhanced therapeutic index values. A comparative Table, where the antibacterial activity of triterpenoid derivatives **4**–**7** was compared with that of some other triterpenoid derivatives previously reported, is available in the Supplementary Materials, Section S2, Table S2.2.

## 4. Conclusions

The scope of this study was to find novel compounds effectively active against worrying superbugs, difficult or impossible to inhibit with available antibiotics and responsible for lethal infections. Such infections develop mainly in nosocomial settings, where immunocompromised patients are highly exposed, causing longer hospitalization times and long-term treatments, with a high social and economic impact. To this end, three natural triterpenoids, deprived of cytotoxic effects on eukaryotic cells, including totally inactive betulin (BET) and betulinic acid (BA), as well as ursolic acid (UA), recognized as active on Gram-positive isolates, have been chemically modified by multi-step and laborious synthetic procedures, as well as arduous purification work up, achieving derivatives **1**–**7**, five of which contained the triphenyl phosphonium (TPP) group, recently reported to promote antibacterial effects. All final compounds and synthetic intermediates were characterized by chemometric-assisted FTIR and NMR spectroscopy, as well as by other analytical techniques, which all confirmed their structure and high purity. When tested for evaluating their antibacterial effects by MIC determinations, using a selection of Gram-positive and Gram-negative clinically isolated superbugs, compounds bearing the TPP group resulted active on all MDR staphylococci and enterococci tested. Specifically, MICs observed for compounds **4**–**7** were lower than those reported so far for other BET, BA and UA derivatives. For the first time, due to the use of TPP, a real activity (MICs 2–16 µg/mL) was conferred to inactive BET and BA (original MICs > 1024 and 256 µg/mL). Moreover, the antibacterial effects of UA against MRSA and MRSE were improved by 32 and 16 times, respectively (MICs = 2 vs. 64 and 32 μg/mL). Structure–activity relationships (SAR) studies evidenced that, while the introduction of residues containing the carbon–carbon triple bond, as ester or amide, did not enhance the antibacterial profile of BET and BA (compounds **2** and **3**), the additional insertion of a TPP group as hexanoate in C-3 of BA, already containing the propargyl amide group, led to significant antibacterial effects (compound **4**). While the simultaneous presence of TPP-hexanoate in C-3 and propargyl carbamate in C-28 of BET led only to weak antibacterial activity (compound **1**), the replacement of propargyl carbamate group with the TPP-hexanoate moiety, leaving free the hydroxyl in C-3 of BET (compound **5**) led to excellent antibacterial effects, thus evidencing that the presence of groups containing the triple bond could be avoided. On the other hand, it seems to be essential to leave free the hydroxyl in C-3, as confirmed by the BET derivative containing two TPP-hexanoate groups on both C-3 and C-28 (compound **6**), which demonstrated good antibacterial activity, but inferior to that of **5**. Despite the presence of propargyl amide in C-28 and the TPP-hexanoate groups in C-3, as BET derivative **4**, UA derivative **7** showed the highest antibacterial effects, probably due to the intrinsic antibacterial activity of the UA core. Our question now is, “Could the antibacterial effects of a new UA derivative functionalized as compound **5** be even more active?” Work is in progress to find the answer. Collectively, these preliminary but very promising microbiologic results pave the way for further experiments with the best-performing compounds **5** and **7** (MICs = 2 μg/mL) on a larger number of Gram-positive isolates to evaluate their mechanism of action by setting up time-killing curves and to assess their cytotoxicity on eukaryotic cells and their possible antibiofilm activity.

## Figures and Tables

**Table 1:** Bacterial strains of this study and their resistance to antibiotics.

Gram-Positive	Resistance	Gram-Negative	Resistance
*S. aureus*	methicillin	*P. aeruginosa* *	carbapenems
*S. epidermidis*	methicillin	*E. coli*	carbapenems
*E. faecium*	vancomycin, teicoplanin	*K. pneumoniae*	carbapenems, carbapenemase (KPC) producers
*E. faecalis*	vancomycin, teicoplanin		

* Isolated from patients with cystic fibrosis.

**Table 2:** Data of potentiometric titrations of **1** and **6**.

Sample	Weight (mg)	* MW	* P^+^ (µmol)	0.1 N HClO_4_ ^§^	** P^+^ (µmol)	** MW	*** Residuals	Error (%)
**1**	1.0	963.1	1.0383	10.40	1.0400	961.5	1.6	0.16
**4**	0.6	933.1	0.6430	6.44	0.6440	931.7	0.8	0.14
**5**	0.7	882.0	0.7936	7.96	0.7960	879.4	2.6	0.29
**6**	1.1	1321.4	1.6648	16.60	1.6600	1325.3	3.9	0.30
**7**	1.5	933.1	1.6075	16.20	1.6202	925.8	7.3	0.78

* Calculated; ** experimental; *** refers to MW; ^§^ = in µL.

**Table 3:** MICs of compounds **4**–**7**, BET, BA and UA against MDR clinical isolates of Gram-positive species obtained from experiments conducted at least in triplicate.

Gram-Positive Isolates	1	4	5	6	7	BET	BA	UA	R.A.
		MICs (µg/mL)							
*S. aureus* (MRSA) 18	64	4	8	8	2	>64	>64	64	256 (O)
*S. epidermidis* (MRSE) 22	32	4	4	8	2	>64	>64	32	128 (O)
*E. faecalis* (VRE) 1 *	64	16	8	16	4	>64	>64	4	256 (V); 64 (T)
*E. faecium* (VRE) 152 *	32	4	2	8	2	>64	>64	2	128 (V); 64 (T)

R.A. = reference antibiotics; * resistant to teicoplanin; VRE = vancomycin-resistant enterococci; MRSA = methicillin-resistant *S. aureus*; MRSE = methicillin-resistant *S. epidermidis*; O = oxacillin; T = teicoplanin; V = vancomycin; BET = betulin; BA = betulinic acid; UA = ursolic acid.

## Data Availability

All research data related to this study are available in the main text and in the Supplementary Materials file associated with this article. Further inquiries can be directed to the corresponding author.

## Supplementary Materials

The following supporting information can be downloaded at: https://www.mdpi.com/article/10.3390/pharmaceutics17121614/s1, Figure S1.1.1.: ATR-FTIR spectrum of BA. Figure S1.1.2: ATR-FTIR spectrum of BET. Figure S1.1.3: ATR-FTIR spectrum of UA. Figure S1.1.4: ATR-FTIR spectrum of 1. Figure S1.1.5: ATR-FTIR spectrum of 2. Figure S1.1.6: ATR-FTIR spectrum of 3. Figure S1.1.7: ATR-FTIR spectrum of 4. Figure S1.1.8: ATR-FTIR spectrum of 5. Figure S1.1.9: ATR-FTIR spectrum of 6. Figure S1.1.10: ATR-FTIR spectrum of 7. Figure S1.2.1: 1H NMR spectrum (600 MHz, CHCl3) of compound 9. Figure S1.2.2: 13C NMR spectrum (151 MHz, CHCl3) of compound 9. Figure S1.2.3: 1H NMR spectrum (600 MHz, CHCl3) of compound 1. Figure S1.2.4: 13C NMR spectrum (151 MHz, CHCl3) of compound 1. Figure S1.2.5: 31P NMR spectrum (243 MHz, CHCl3) of compound 1. Figure S1.2.6: 1H NMR spectrum (600 MHz, CHCl3) of compound 2. Figure S1.2.7: 13C NMR spectrum (151 MHz, CHCl3) of compound 2. Figure S1.2.8: 1H NMR spectrum (600 MHz, CHCl3) of compound 3. Figure S1.2.9: 13C NMR spectrum (151 MHz, CHCl3) of compound 3. Figure S1.2.10: 1H NMR spectrum (600 MHz, CHCl3) of compound 4. Figure S1.2.11: 13C NMR spectrum (151 MHz, CHCl3) of compound 4. Figure S1.2.12: 31P NMR spectrum (243 MHz, CHCl3) of compound 4. Figure S1.2.13: 1H NMR spectrum (600 MHz, CHCl3) of compound 11. Figure S1.2.14: 13C NMR spectrum (151 MHz, CHCl3) of compound 11. Figure S1.2.15: 1H NMR spectrum (600 MHz, CHCl3) of compound 12. Figure S1.2.16: 13C NMR spectrum (151 MHz, CHCl3) of compound 12. Figure S1.2.17: 1H NMR spectrum (600 MHz, CHCl3) of compound 5. Figure S1.2.18: 13C NMR spectrum (151 MHz, CHCl3) of compound 5. Figure S1.2.19: 31P NMR spectrum (243 MHz, CHCl3) of compound 5. Figure S1.2.20: 1H NMR spectrum (600 MHz, CHCl3) of compound 6. Figure S1.2.21: 13C NMR spectrum (151 MHz, CHCl3) of compound 6. Figure S1.2.22: 31P NMR spectrum (243 MHz, CHCl3) of compound 6. Figure S1.2.23: 1H NMR spectrum (600 MHz, CHCl3) of compound 13. Figure S1.2.24: 13C NMR spectrum (151 MHz, CHCl3) of compound 13. Figure S1.2.25: 1H NMR spectrum (600 MHz, CHCl3) of compound 7. Figure S1.2.26: 13C NMR spectrum (151 MHz, CHCl3) of compound 7. Figure S1.2.27: 31P NMR spectrum (243 MHz, CHCl3) of compound 7. Table S2.1: MICs of compounds 1–7, BET, BA and UA against MDR clinical isolates of Gram-positive and Gram-negative species obtained from experiments conducted at least in triplicate. Table S2.2: Comparison between the antibacterial effects of BET derivatives 1, 5, 6, BA derivative 4 and UA derivative 7, with those of other BET, BA and UA derivatives previously reported. Scheme S1.1.1: Propargyl—allenyl tautomerization process.
