# Supplementary material for: Synthesis and Characterization of Triphenyl Phosphonium-Modified Triterpenoids with Never Reported Antibacterial Effects Against Clinically Relevant Gram-Positive Superbugs
Source: Pharmaceutics. 2025 Dec 16;17(12):1614. doi: 10.3390/pharmaceutics17121614 (PMC12736544; doi:10.3390/pharmaceutics17121614)
Supplement: Supplementary file 1 [file pharmaceutics-17-01614-s001.zip › pharmaceutics-4010311-supplementary.pdf]

pharmaceutics
MDPI

Supplementary Materials

Synthesis and Characterization of Triphenyl Phosphonium-Modified Triterpenoids with Never Reported Antibacterial Effects Against Clinically Relevant Gram-positive Superbugs

Dafni Graikioti [1], Constantinos M. Athanassopoulos [1,*], Anna Maria Schito [2] and Silvana Alfei [3,*]

Department of Chemistry, University of Patras, University Campus, 26504 Rio, Achaias, Patras, Greece; dafnigraikioti@upnet.gr
[2] Department of Surgical Sciences and Integrated Diagnostics (DISC), University of Genoa, Viale Benedetto XV, 6, 16132 Genova, Italy; amschito@unige.it
[3] Department of Pharmacy, University of Genoa, Viale Cembrano, 16148 Genoa, Italy
* Correspondence: kath@upatras.gr (C.M.A.); alfei@difar.unige.it (S.A.), Tel.: +39-010-355-2296 (S.A.)

Section S1. Spectrophotometric Analyses.

Section S1.1. ATR-FTIR of BA, BET, UA and Compounds **1-7**.

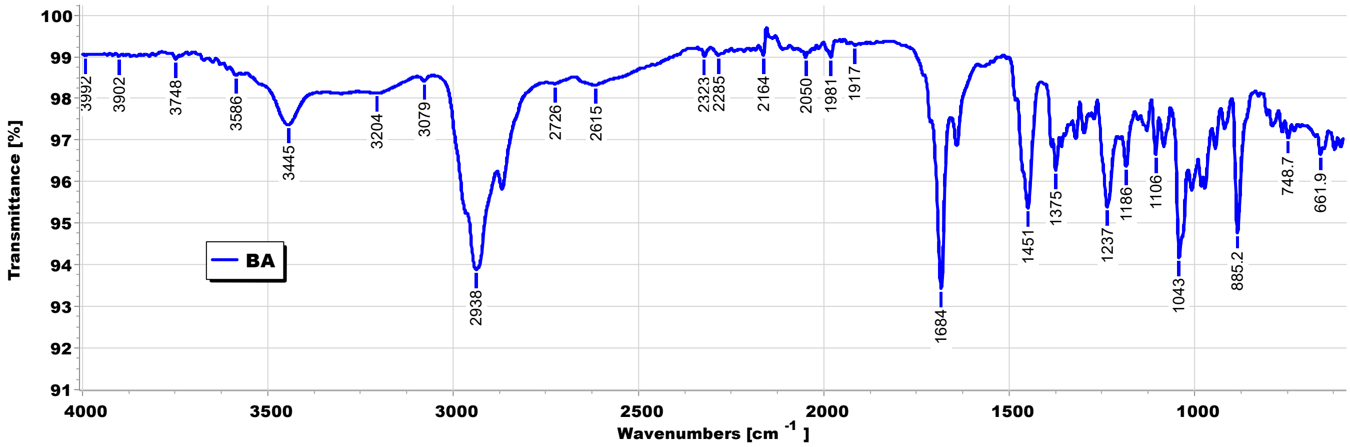

Figure S1.1.1. ATR-FTIR spectrum of BA.

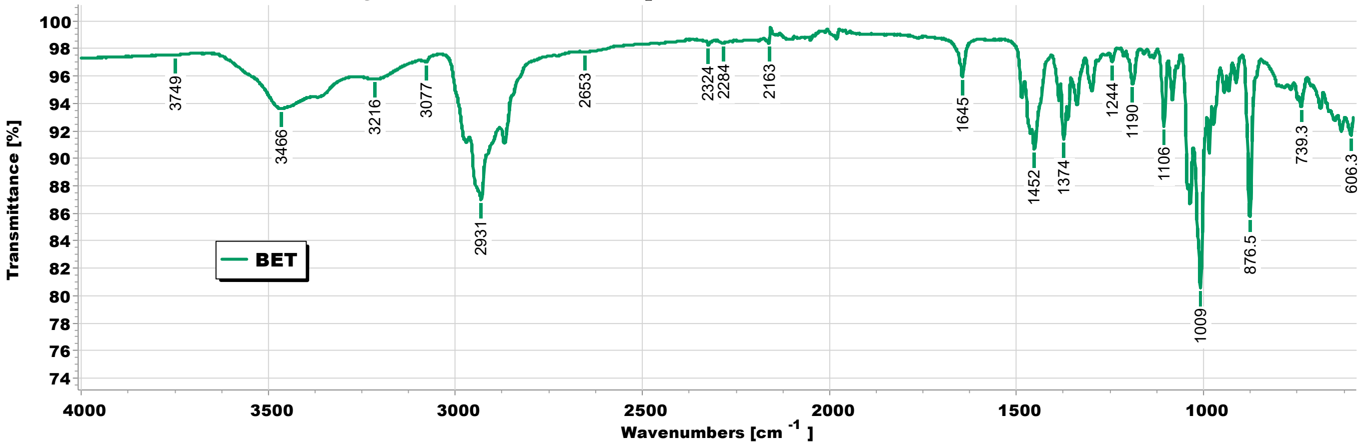

Figure S1.1.2. ATR-FTIR spectrum of BET.

 https://doi.org/10.3390/pharmaceutics17121614

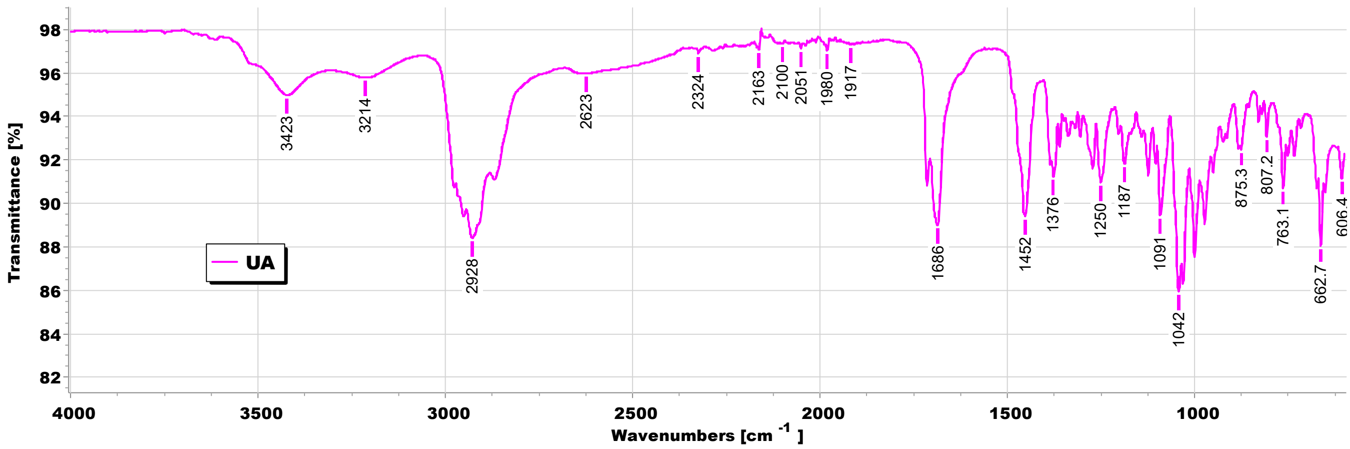

Figure S1.1.3. ATR-FTIR spectrum of UA.

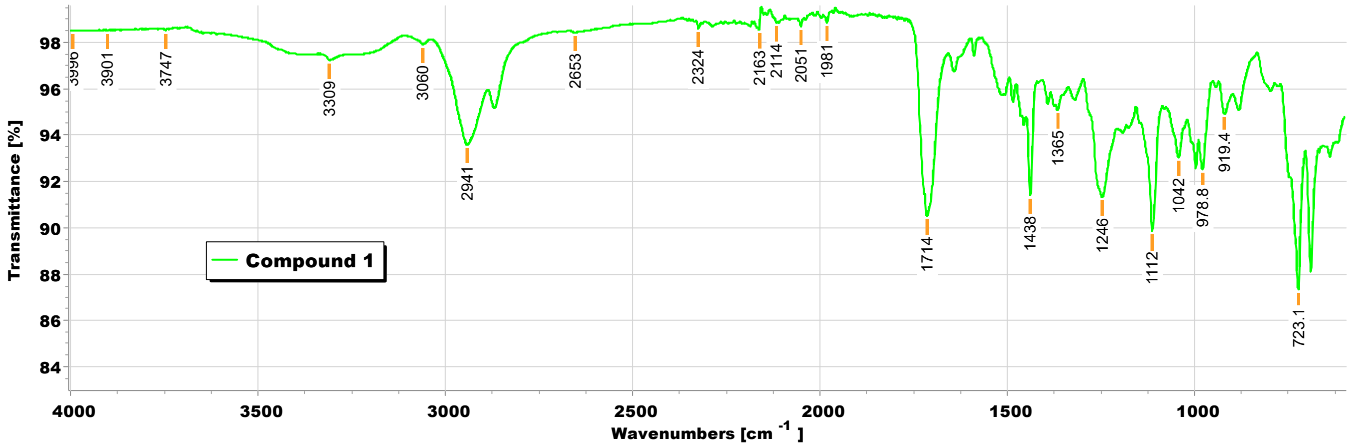

Figure S1.1.4. ATR-FTIR spectrum of **1**.

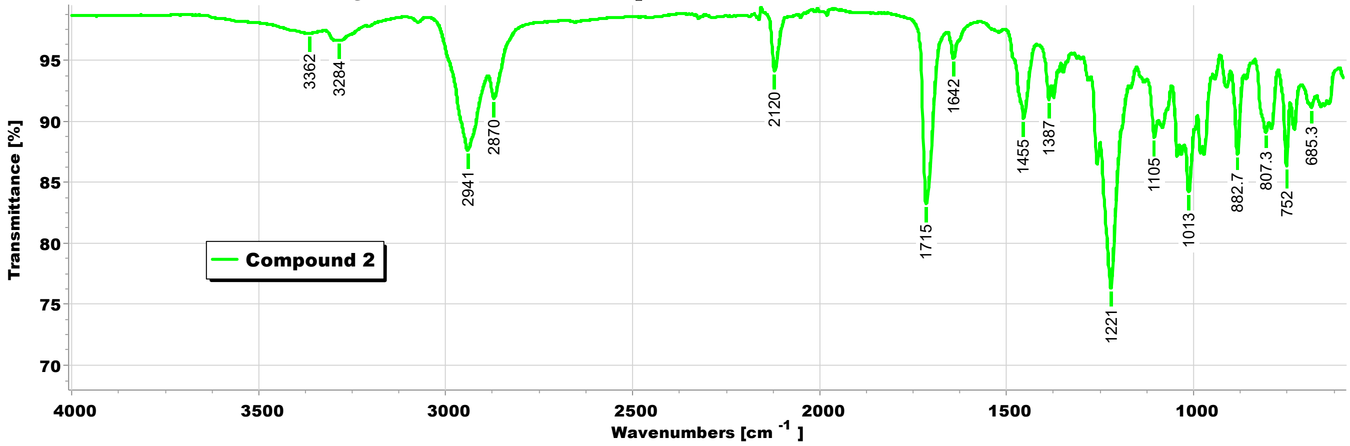

Figure S1.1.5. ATR-FTIR spectrum of **2**.

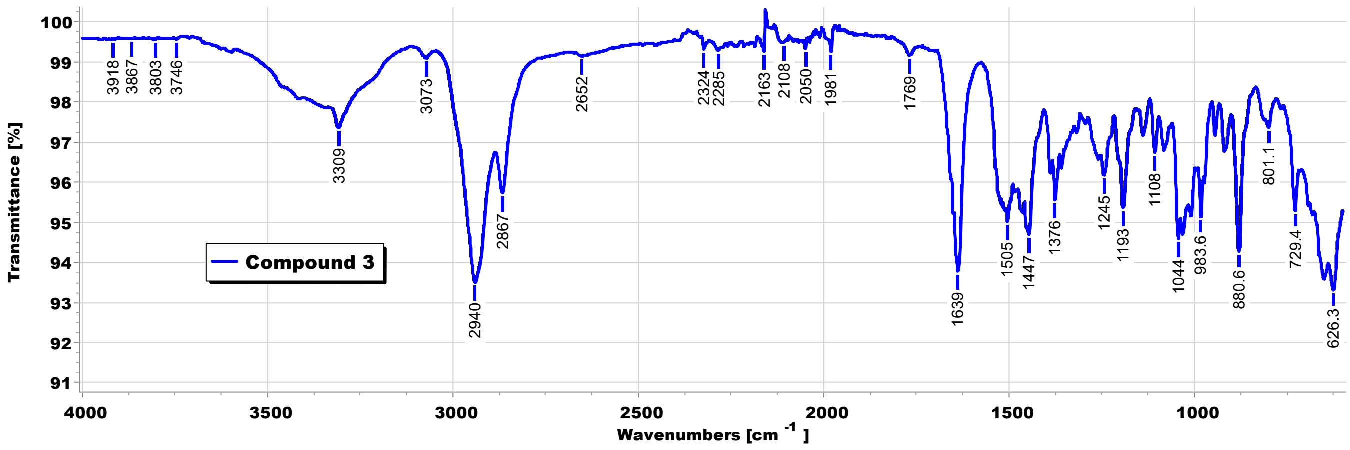

Figure S1.1.6. ATR-FTIR spectrum of **3**.

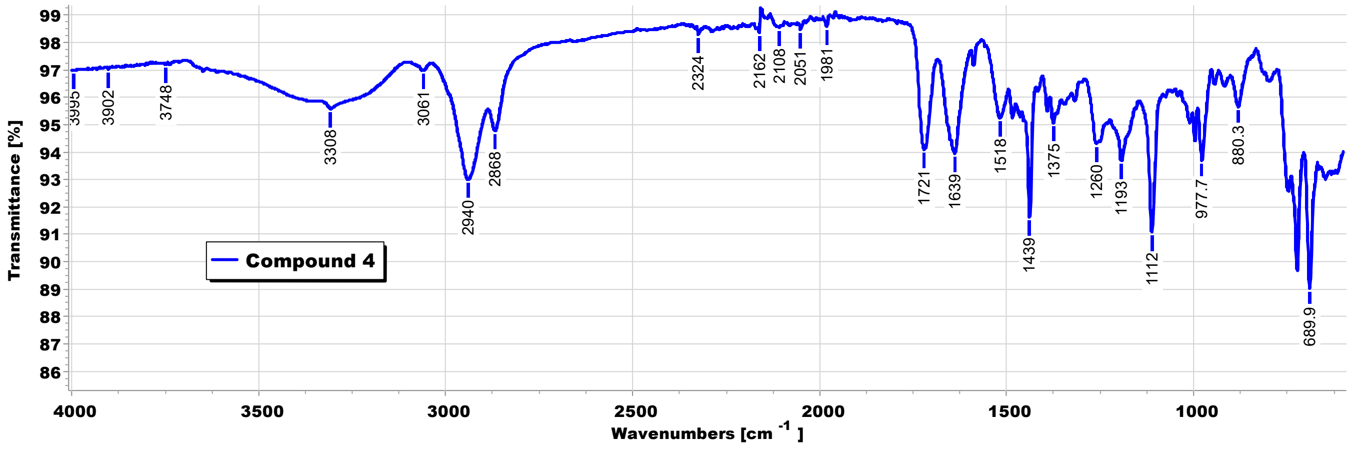

Figure S1.1.7. ATR-FTIR spectrum of **4**.

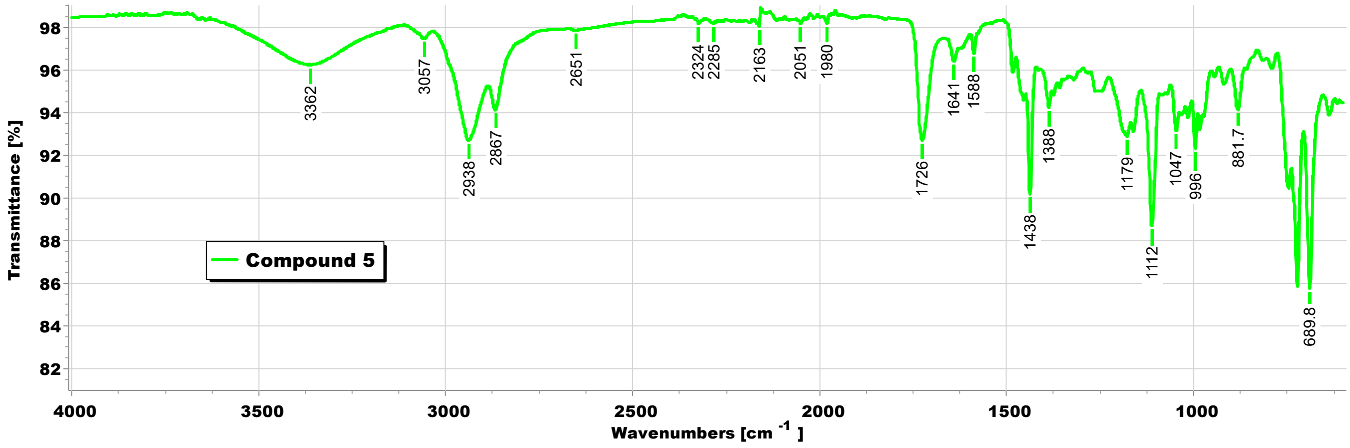

Figure S1.1.8. ATR-FTIR spectrum of **5**.

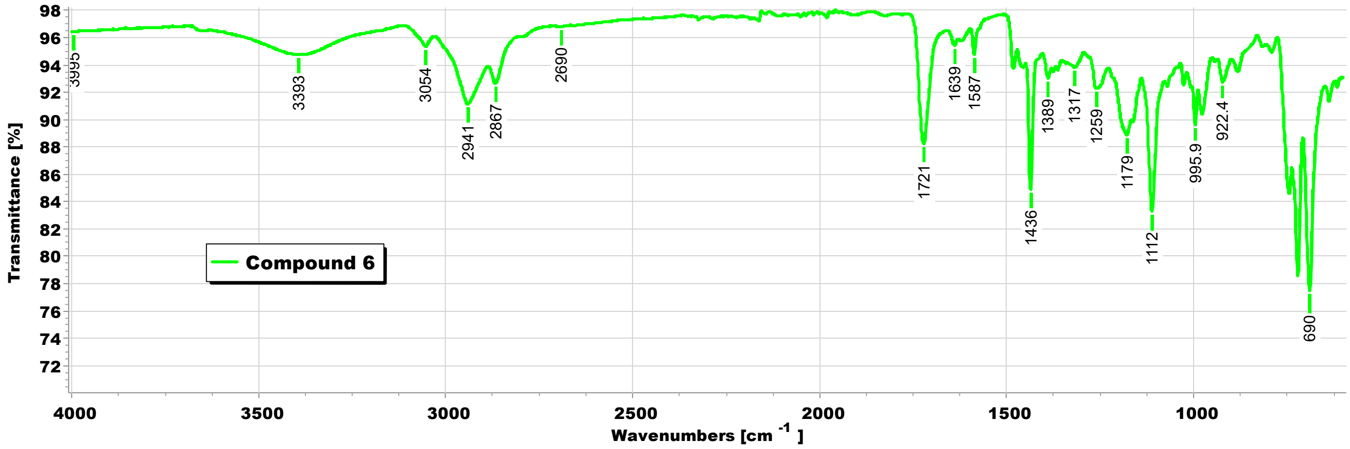

Figure S1.1.9. ATR-FTIR spectrum of **6**.

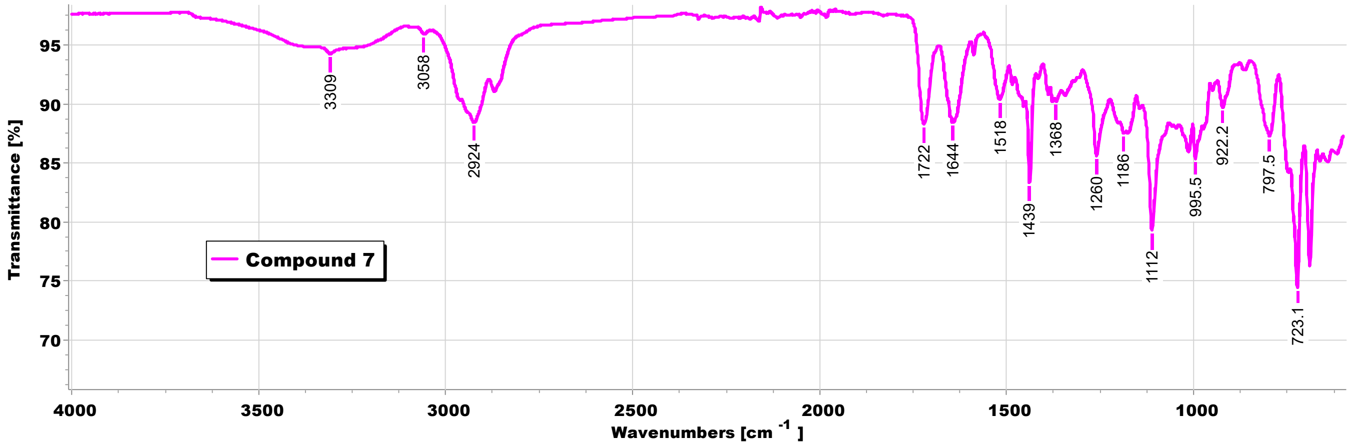

Figure S1.1.10. ATR-FTIR spectrum of **7**.

ATR-FTIR Bands Assignment

In BA spectrum weak bands at 3445 and 3079 cm^{-1} due to the O-H and =C-H stretching respectively, were observable. Bands related to the C-H stretching of methyl and methylene groups were present at 2939, 2965 and 2869 cm^{-1}, while the C=O stretching of carboxylic acid group in C-28 was detectable at 1684 cm^{-1}. Bands observed at 1451, 1043 and 885 cm^{-1} were assigned to the C-H banding, C-O stretching and -C=C- banding, respectively. The spectra of BA derivative **3** showed bands similar to those of BA for the O-H and =C-H stretching (3309 and 3073 cm^{-1}), for the C-H stretching of methyl and methylene groups (2940 and 2867 cm^{-1}), for the C-H banding (1505, 1447 cm^{-1}) and for the -C=C- bond (881 cm^{-1}). In addition, a new band at 1639 cm^{-1} was observable for the C=O stretching of the amide group, which substituted that of C=O of carboxylic acid previously observable at 1684 cm^{-1}. The stretching band of C≡C–H bond of the propyne group was not detectable, as in other compounds containing the propargyl amide group (**4** and **7**). This fact was due to the weak acidity of proton atoms of the methylene propargyl groups (CH_2-C≡C–H), which can tautomerize from form I to provide allenyl derivatives (II), which no longer contain the triple bond [67] (Scheme S1.1.1).

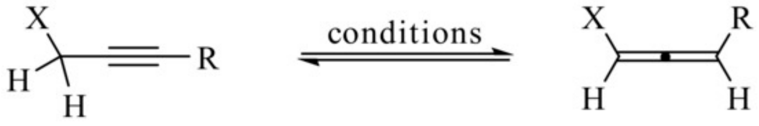

I **II**

Scheme S1.1.1. Propargyl–allenyl tautomerization process [67]. The Scheme is a reproduction from an open access article published on Molecules, and no special permission is required to reuse all or part of article, including figures and tables, since published under an open access Creative Common CC BY license.

In our specific case, tautomerism was further promoted by the presence of the amide function, where the lone pair on the nitrogen atom can delocalize towards the carboxyl group to form new compounds with a negative formal charge on the oxygen and a positive one on the nitrogen, thus augmenting the acidity of the H atoms in CH_2, necessary for tautomerism. In the spectrum of the other BA derivative **4**, having new groups on both the secondary OH group on C-3 and on the carboxylic acid group in C-28, in addition to bands already detected in the spectrum of BA for the =C-H stretching (3061 cm^{-1}), for the C-H stretching of methyl and methylene groups (2940, 2868 cm^{-1}) and for the C-H banding (1439 cm^{-1}), new bands of stretching C=O of the amide and ester groups were observable at 1639 cm^{-1} and 1721 cm^{-1}. Moreover, two very strong bands due to the aliphatic C-P stretching were present at 723 and 690 cm^{-1}, the C-O band shifted to 1112 cm^{-1} and like for **3**, the C≡C–H bond of the propyne group was not detectable. Similarly to BA the spectrum of BET showed weak bands at 3486 and 3077 cm^{-1} due to the O-H and =C-H stretching respectively, bands related to the C-H stretching of methyl and methylene groups at 2970, 2931 and 2868 cm^{-1}, no stretching C=O band was present due to the absence of carboxylic acid group, replaced by a primary alcohol (CH_2OH) group in C-28, while at 1452, 1009 and 887 cm^{-1} were visible the C-H banding, the C-O stretching and the -C=C- banding, respectively. BET derivatives **2** and **5** showed bands like those of BET for the O-H and =C-H stretching (3362, 3339 and 3057, 3054 cm^{-1}), for the C-H stretching of methyl and methylene groups (2938, 2867 and 2941, 2867 cm^{-1}) and for the C-H banding (1438 and 1436 cm^{-1}), while the C-O stretching shifted to 1112 cm^{-1} as in the case of compound **4**. In addition, in the spectrum of **2**, containing the propargyl ester in C-28, the C≡C–H stretching of the triple bond was this time clearly visible at 2120 cm^{-1} as weak band. Conversely, in the spectrum of **5** containing the TPP-hexyl ester, new bands at 1726 and at 723, 690 cm^{-1} were observed, due to the C=O stretching of ester and to the stretching of aliphatic C-P. BET derivatives **1** and **6** showed bands like those of BET for the =C-H and C-H stretching (3060, 2941, 2869 and 3054, 2941, 2867 cm^{-1}) and for the C-H banding (1438 and 1436 cm^{-1}), while the C-O stretching shifted to 1112 cm^{-1} as in the case of compound **2**, **4** and **5**. In addition, in the spectrum of **1**, an additional band for the C-O stretching was visible at 1246 cm^{-1}. In the spectrum of **1**, containing both the propargyl carbamate group in C-28, and the TPP-hexyl ester in C-3, the strong and large band of C=O stretching of carbamate and ester groups was visible at 1714 cm^{-1}, while the bands of the aliphatic C-P stretching were observable at 723, 689 cm^{-1}. As in the cases of compounds **3** and **4**, the C≡C–H stretching of the triple bond was not visible. Conversely, in the spectrum of **6** containing two TPP-hexyl ester groups in C-3 and C-28, the band of C=O stretching of ester groups was visible at 1721 cm^{-1}, while the bands of the aliphatic C-P stretching were observable at 745, 722 and 690 cm^{-1}. Similarly to BA, the spectrum of UA showed a weak band at 3423 cm^{-1} due to the O-H stretching, bands related to the C-H stretching of methyl and methylene groups at 2976, 2951, 2928 and 2869 cm^{-1}, a C=O stretching band at 1686 cm^{-1}, associated to a new band at 1714 cm^{-1}, due to carboxylic acid group in C-28, while at 1452 and 1030, 1042 cm^{-1} bands were visible for the C-H banding and the C-O stretching. The band of =C-H stretching and of the -C=C- banding were not visible. UA derivative **7** showed bands like those of UA for the C-H stretching of methyl and methylene groups (2962, 2924, 2869 cm^{-1}) and for the C-H banding (1439 cm^{-1}), while the C-O stretching shifted to 1112 cm^{-1} as in the case of compounds **1**, **2**, **4, 5** and **6**. In addition, the band of =C-H stretching, not detected in the spectrum of UA, was visible at 3058 cm^{-1}. New bands

were then detected at 1722, 1644 and 746, 729, 690 cm-1, related to the C=O stretching of the ester (1722) and amide (1644) groups and to the aliphatic stretching C-P. As in the case of compounds **3** and **4**, the CΞCH stretching of the triple bond was not visible.

Section S1.2. Copies of ^{1}H, ^{13}C NMR and ^{31}P NMR spectra of Most Significant Compounds of This Study.

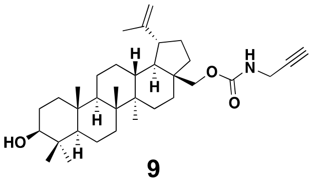

9

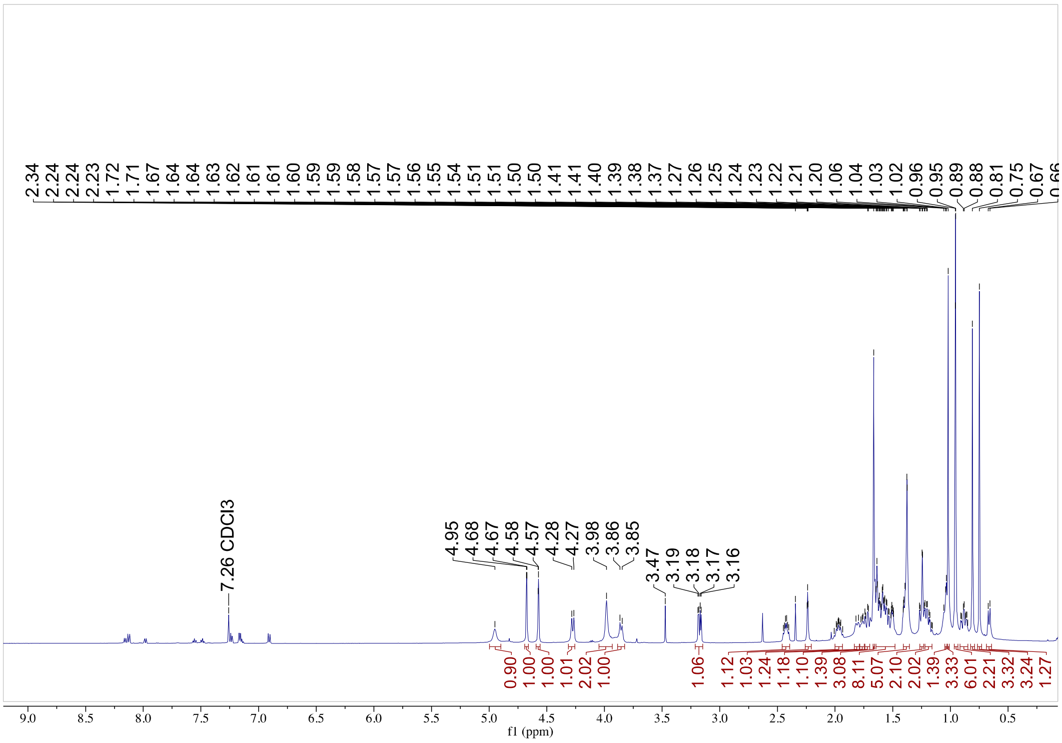

Figure S1.2.1. ^{1}H NMR spectrum (600 MHz, $CHCl_3$) of compound **9**.

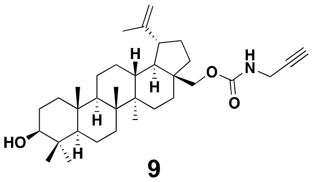

9

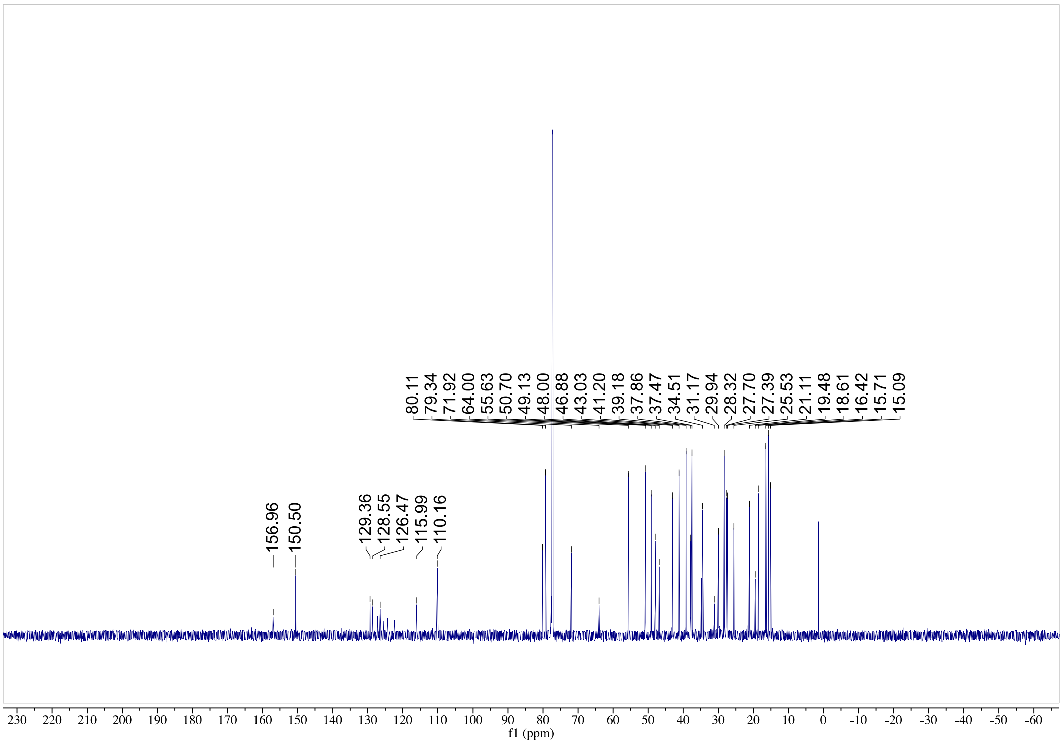

Figure S1.2.2. ^{13}C NMR spectrum (151 MHz, $CHCl_3$) of compound **9**.

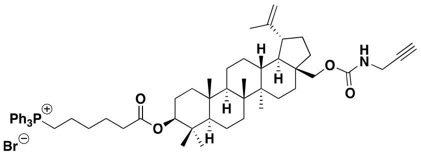

1

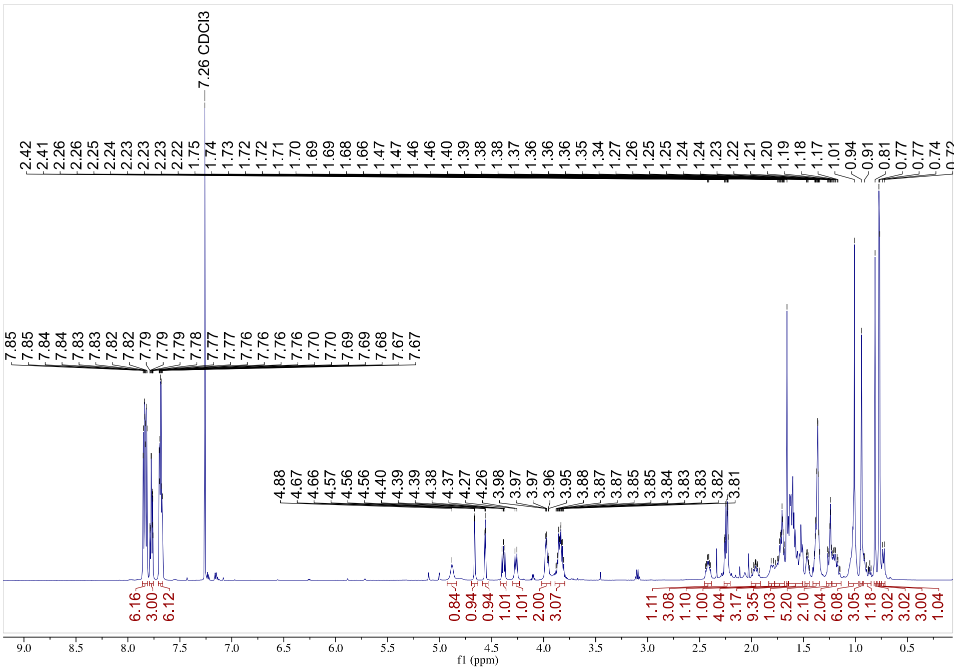

Figure S1.2.3. ^{1}H NMR spectrum (600 MHz, $CHCl_3$) of compound **1**.

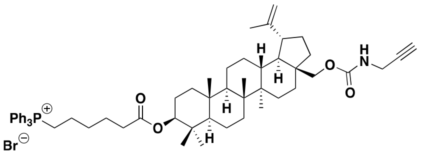

1

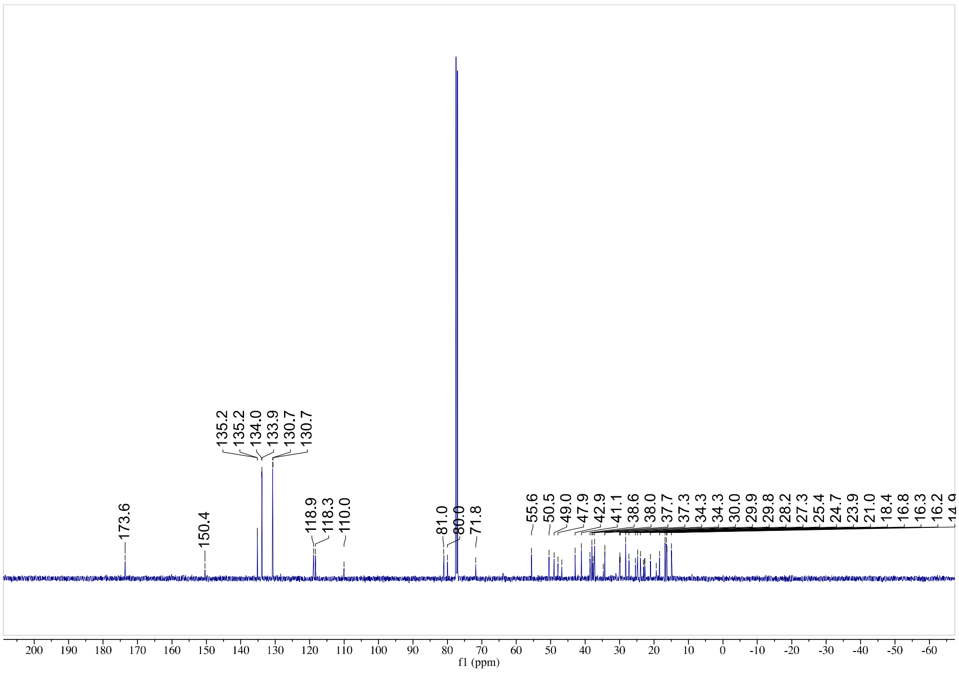

Figure S1.2.4. ^{13}C NMR spectrum (151 MHz, $CHCl_3$) of compound **1**.

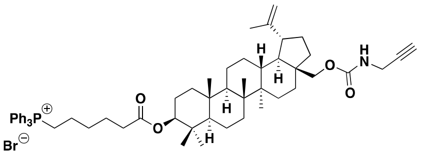

1

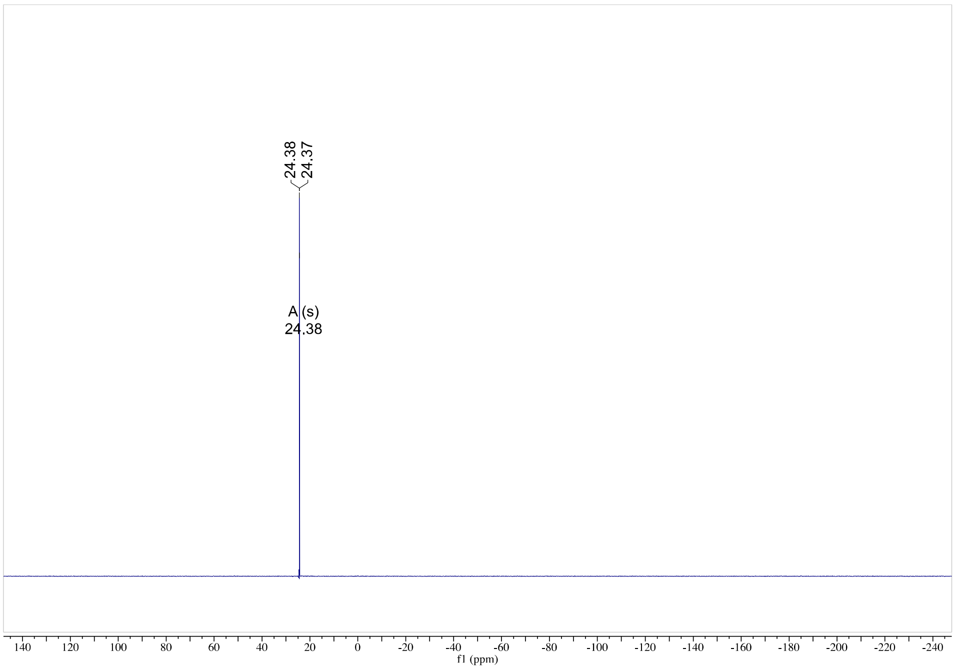

Figure S1.2.5. ^{31}P NMR spectrum (243 MHz, $CHCl_3$) of compound **1**.

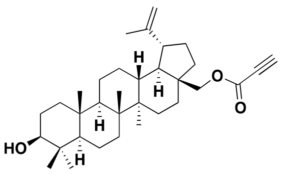

2

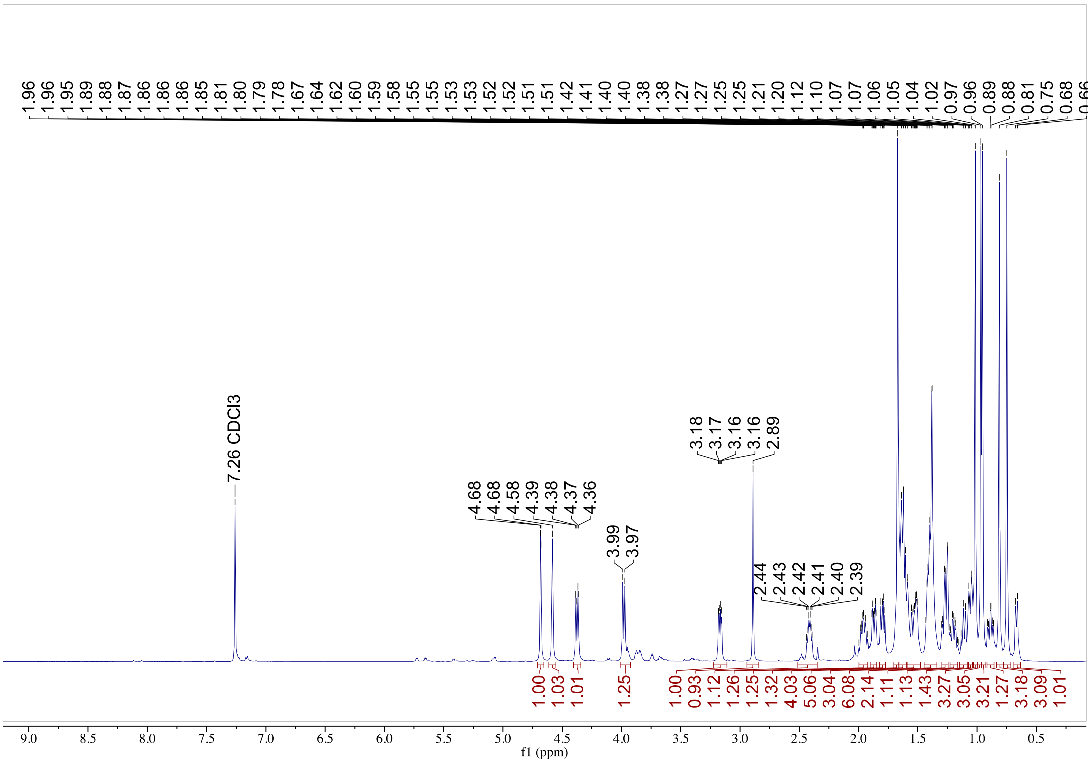

Figure S1.2.6. ^{1}H NMR spectrum (600 MHz, $CHCl_3$) of compound **2**.

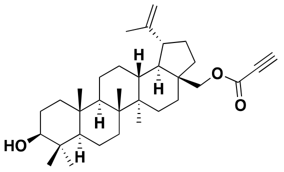

2

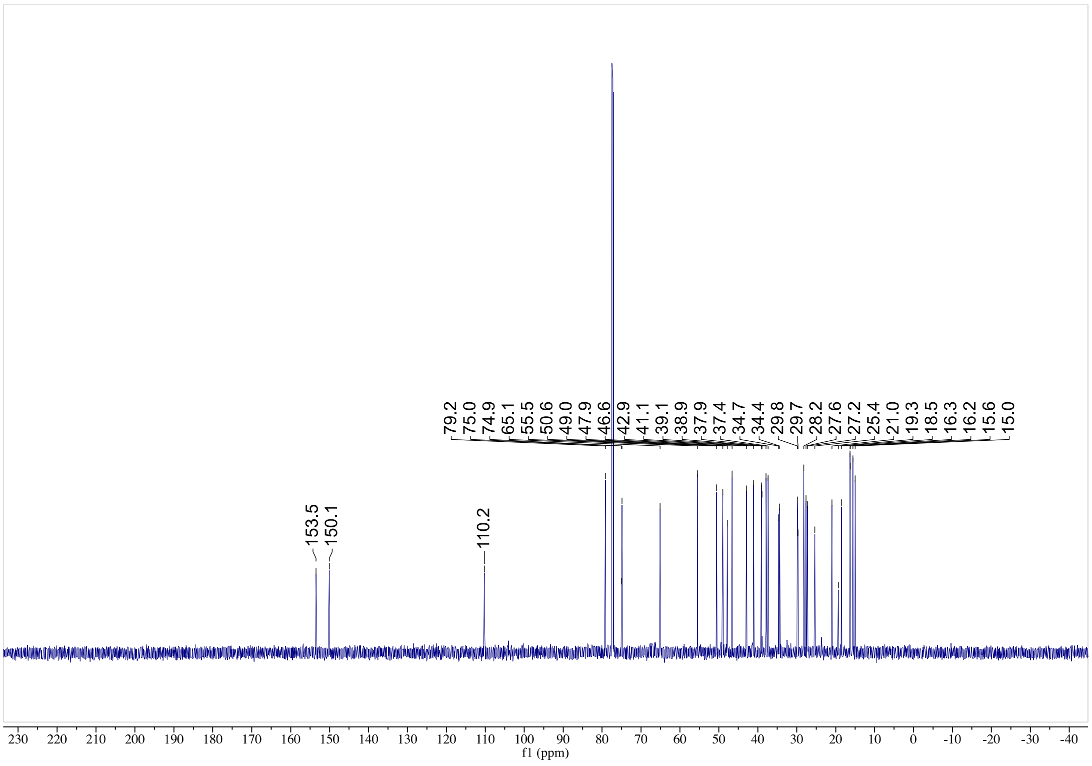

Figure S1.2.7. ^{13}C NMR spectrum (151 MHz, $CHCl_3$) of compound **2**.

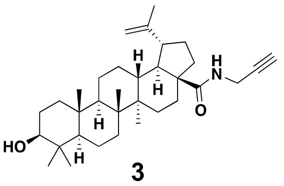

3

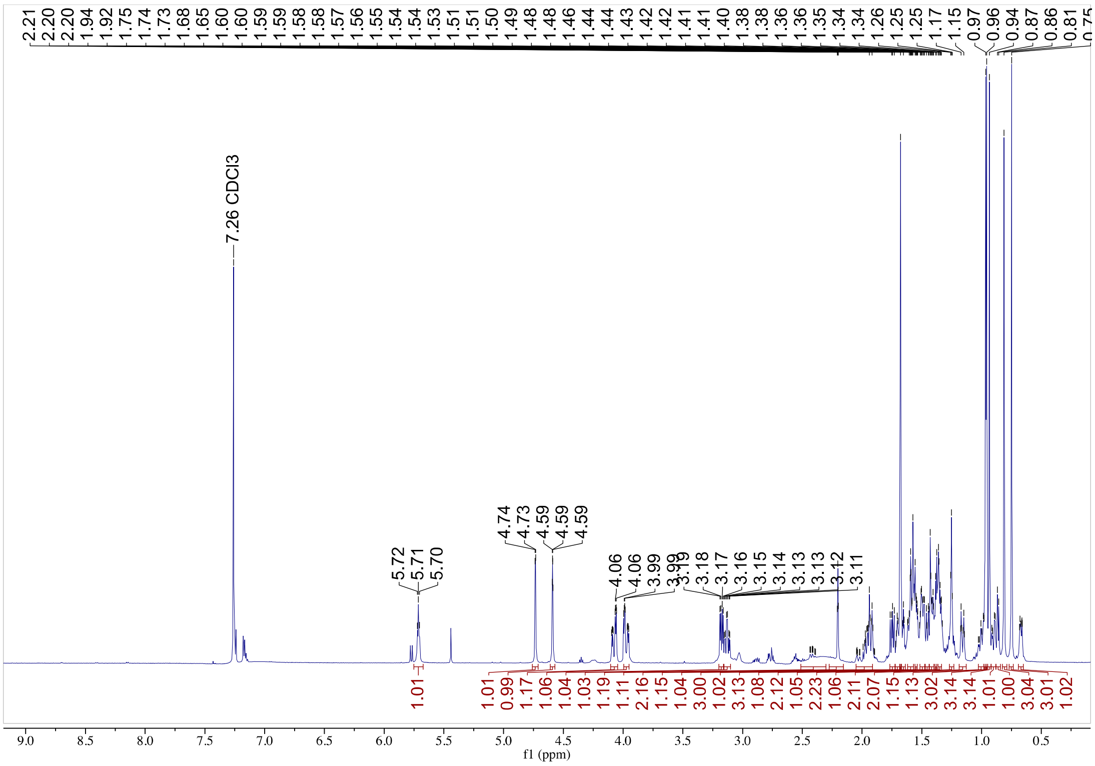

Figure S1.2.8. ^{1}H NMR spectrum (600 MHz, $CHCl_3$) of compound **3**.

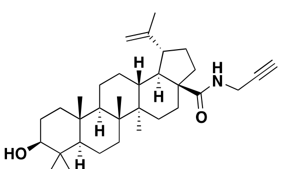

3

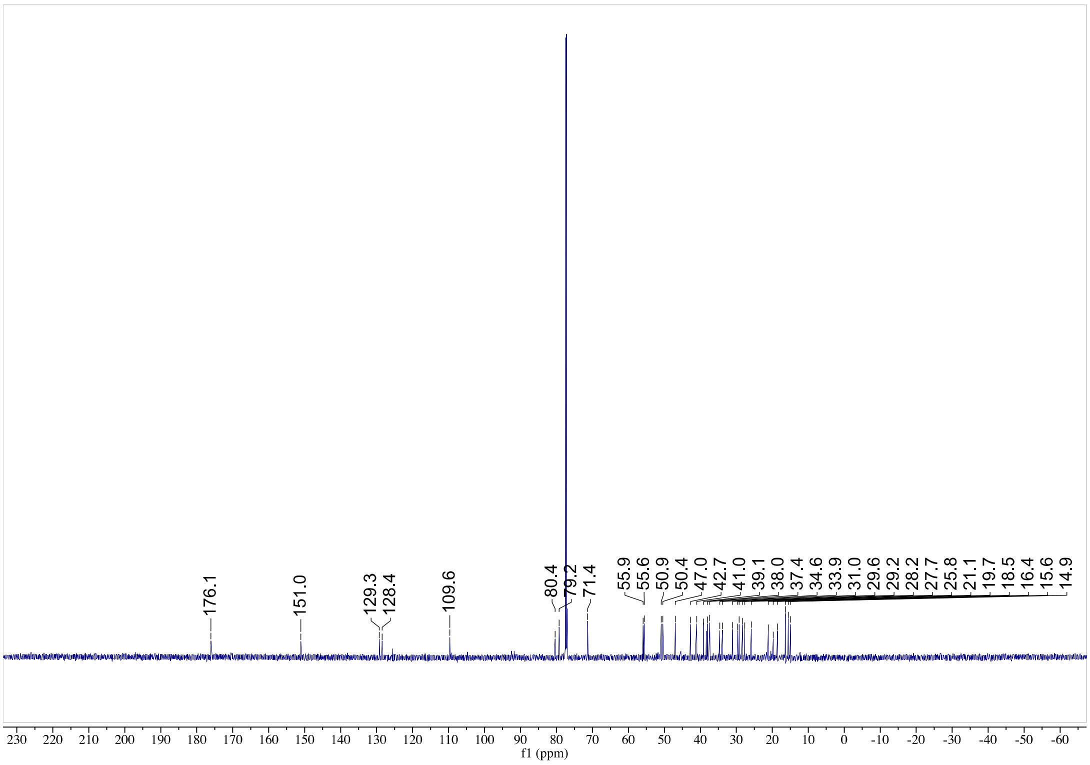

Figure S1.2.9. ^{13}C NMR spectrum (151 MHz, $CHCl_3$) of compound **3**.

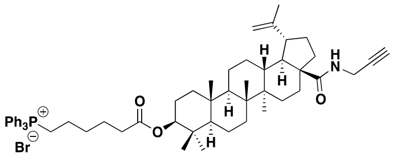

4

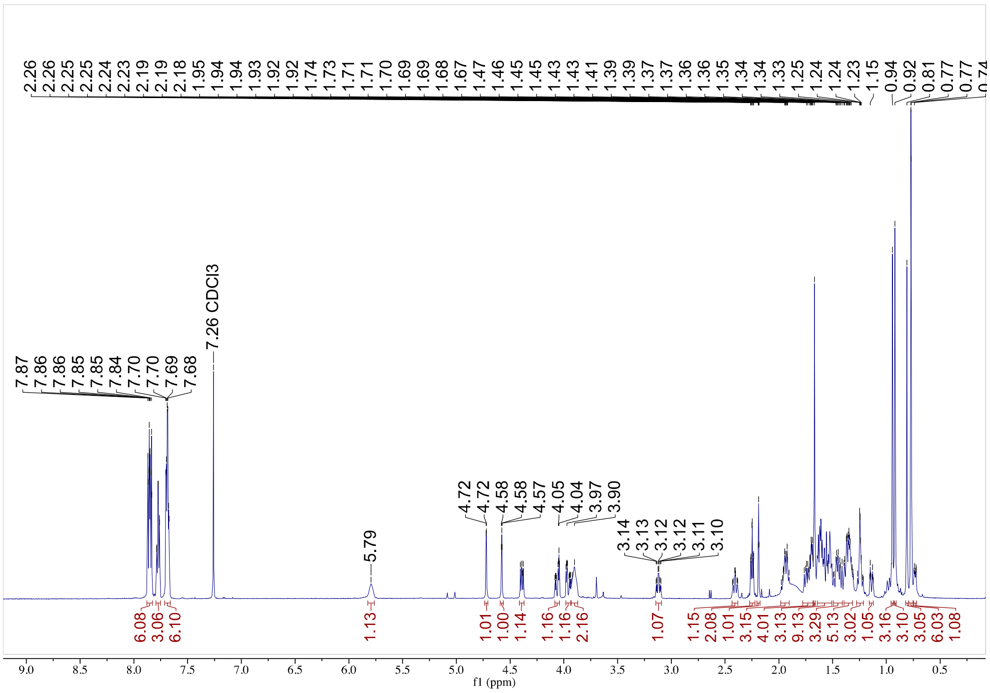

Figure S1.2.10. ^{1}H NMR spectrum (600 MHz, $CHCl_3$) of compound **4**.

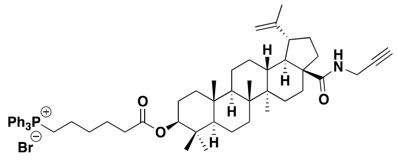

4

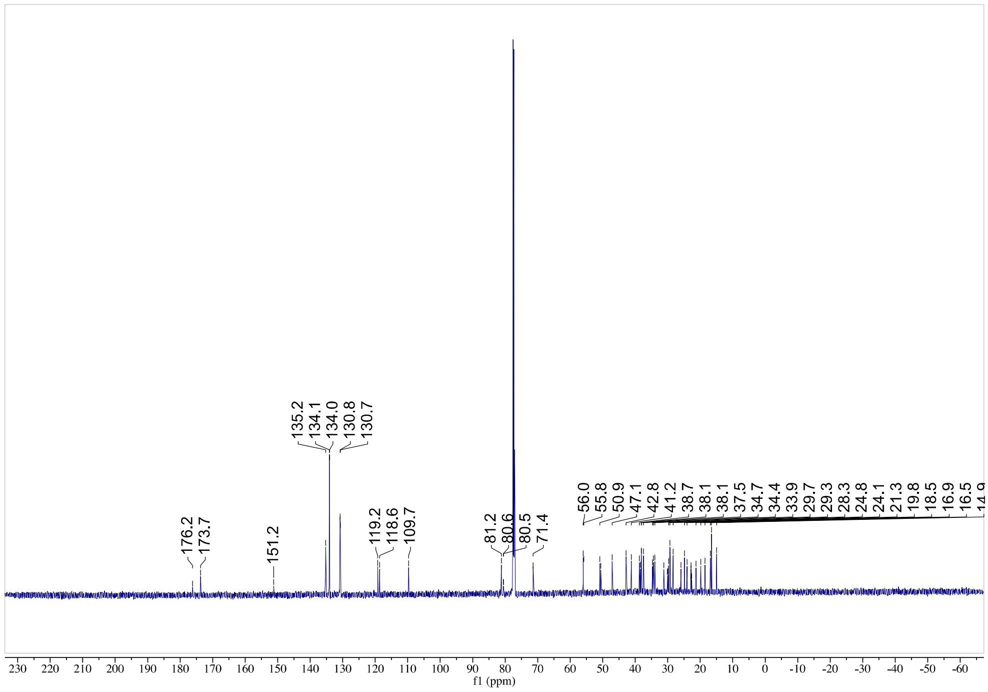

Figure S1.2.11. ^{13}C NMR spectrum (151 MHz, $CHCl_3$) of compound **4**.

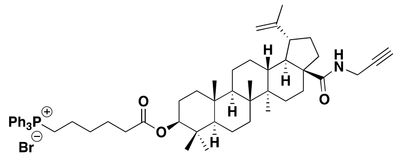

4

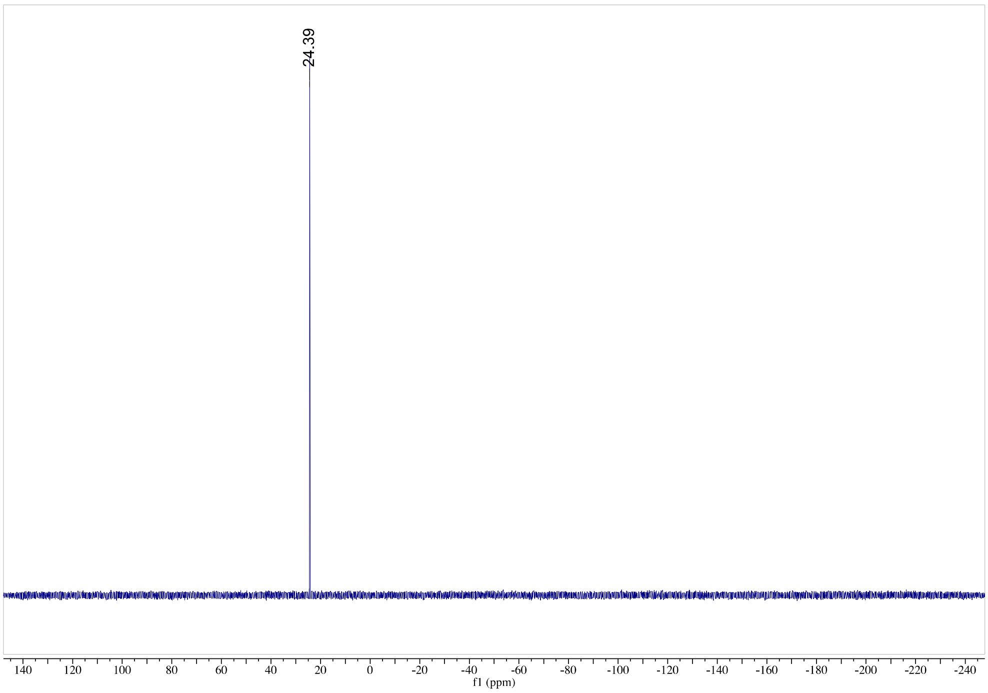

Figure S1.2.12. ^{31}P NMR spectrum (243 MHz, $CHCl_3$) of compound **4**.

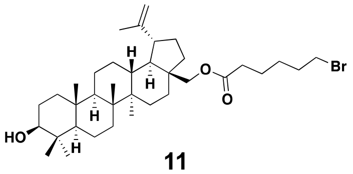

11

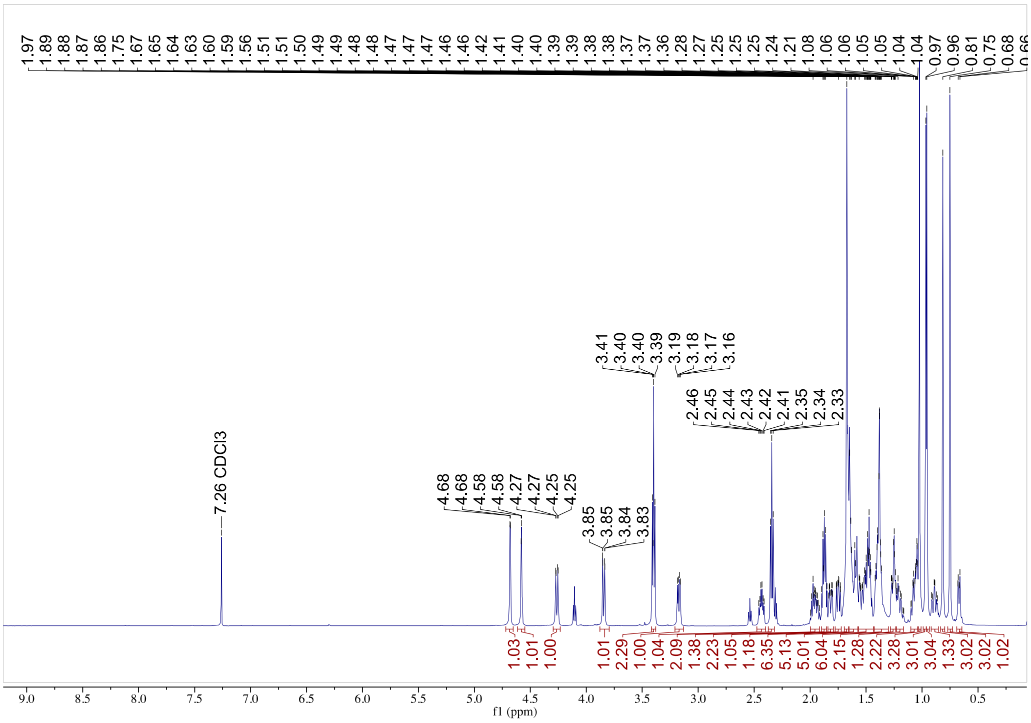

Figure S1.2.13. ^{1}H NMR spectrum (600 MHz, $CHCl_3$) of compound **11**.

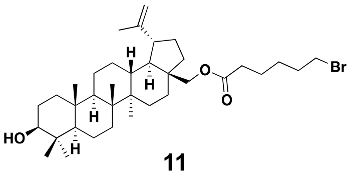

11

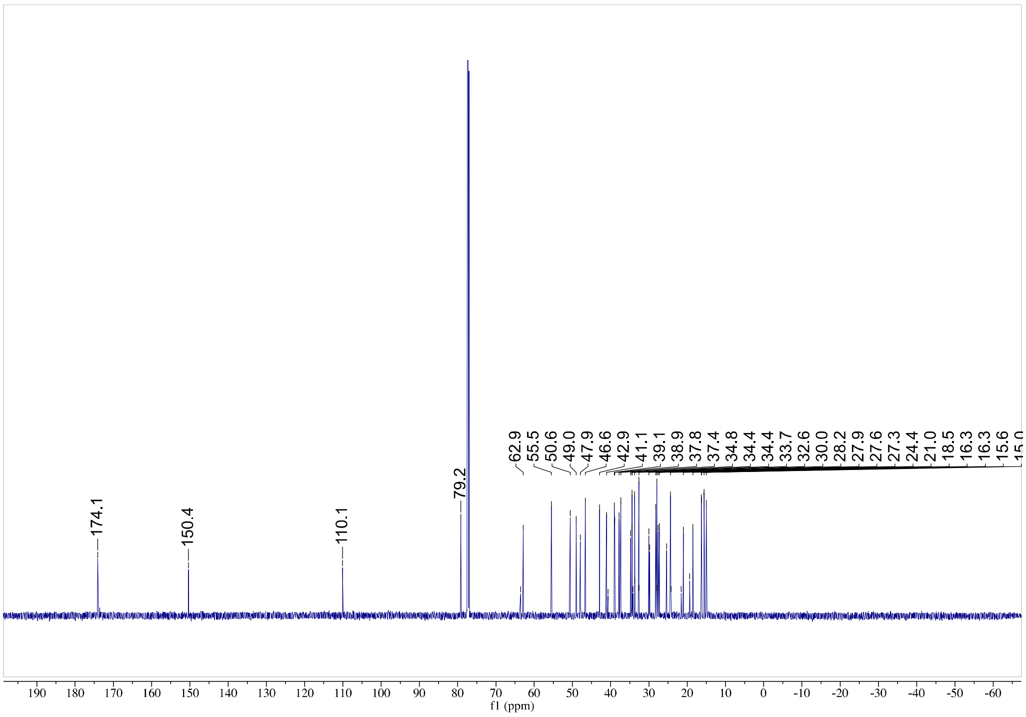

Figure S1.2.14. ^{13}C NMR spectrum (151 MHz, $CHCl_3$) of compound **11**.

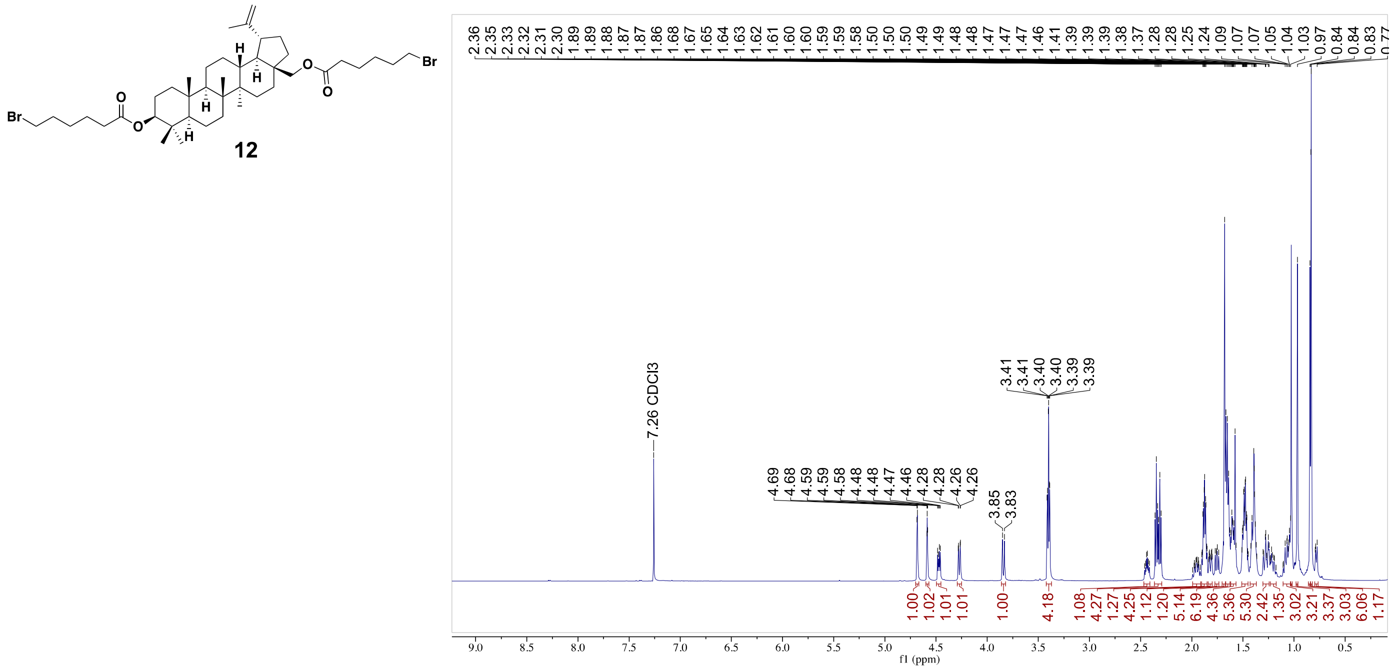

Figure S1.2.15. ^{1}H NMR spectrum (600 MHz, $CHCl_3$) of compound **12**.

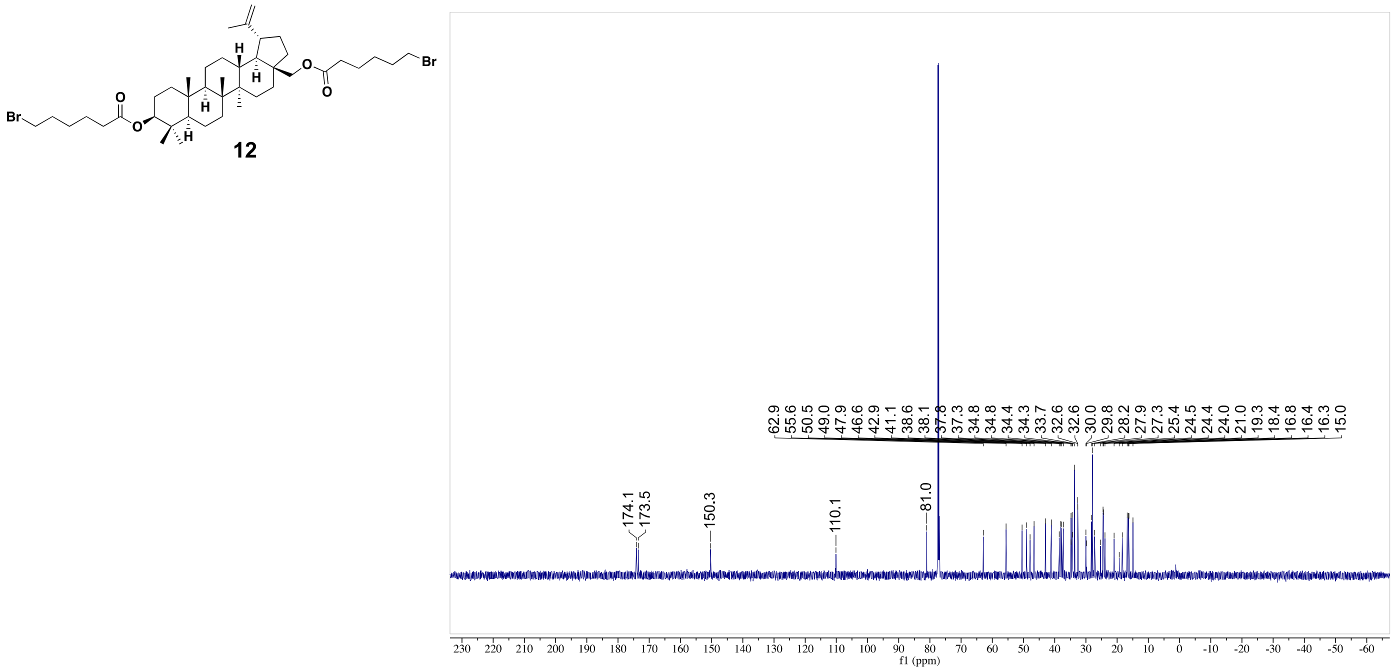

Figure S1.2.16. ^{13}C NMR spectrum (151 MHz, $CHCl_3$) of compound **12**.

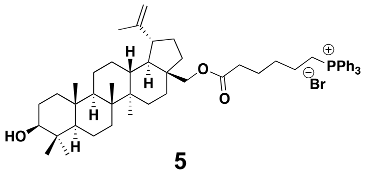

5

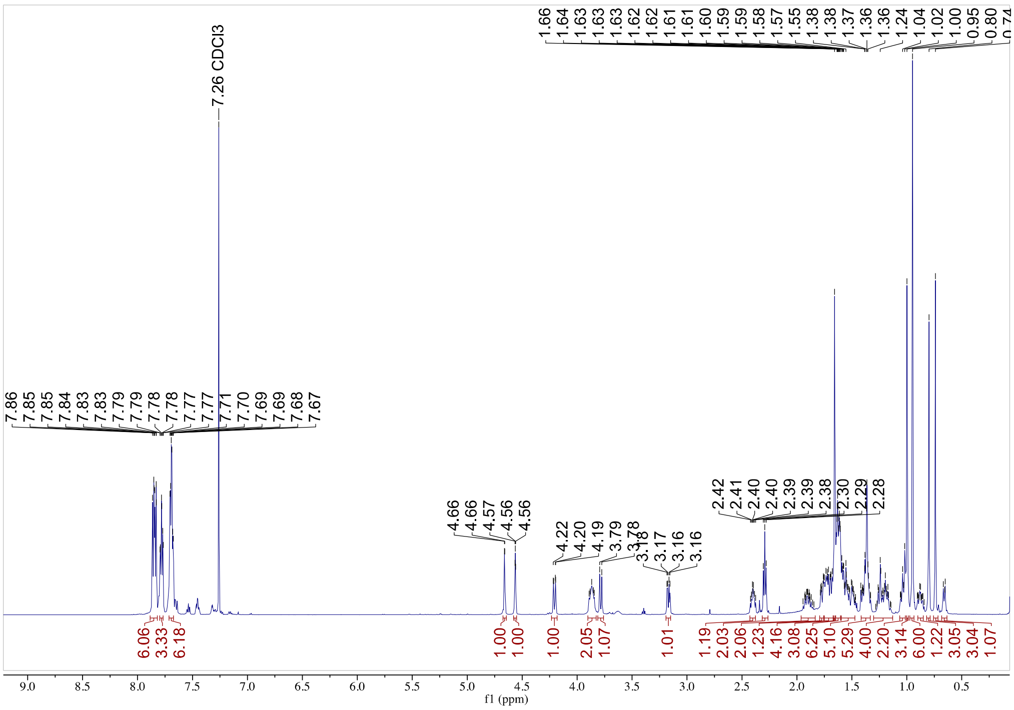

Figure S1.2.17. ^{1}H NMR spectrum (600 MHz, $CHCl_3$) of compound **5**.

.

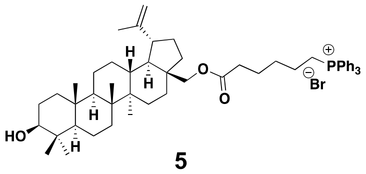

5

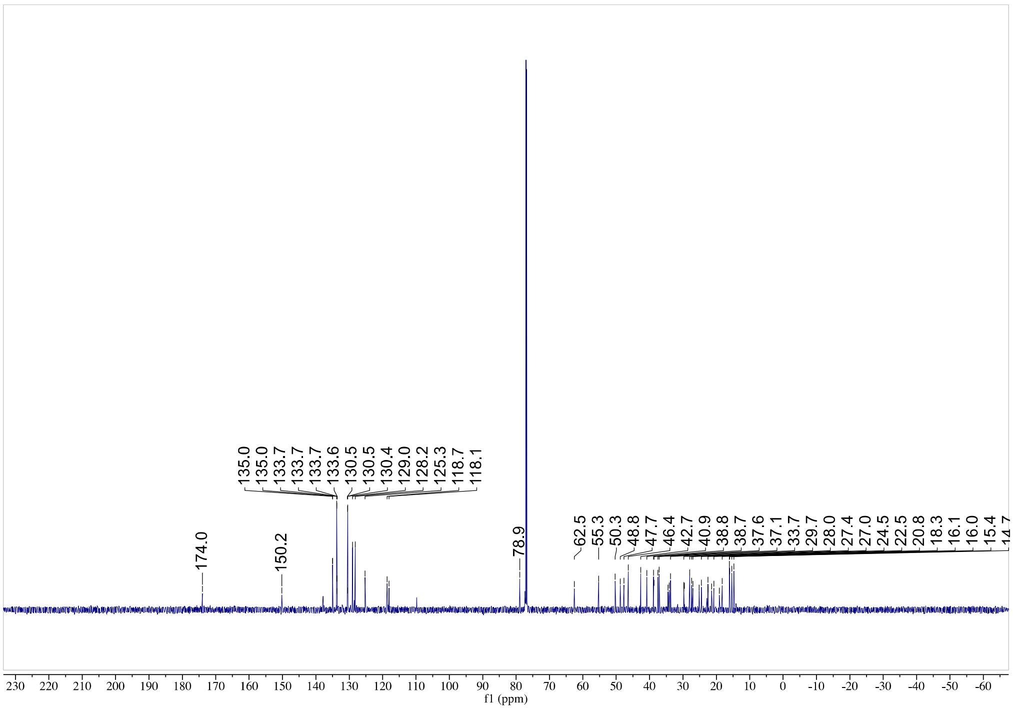

Figure S1.2.18. ^{13}C NMR spectrum (151 MHz, $CHCl_3$) of compound **5**.

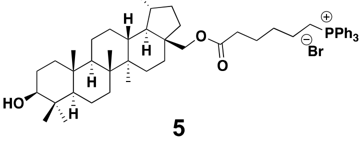

5

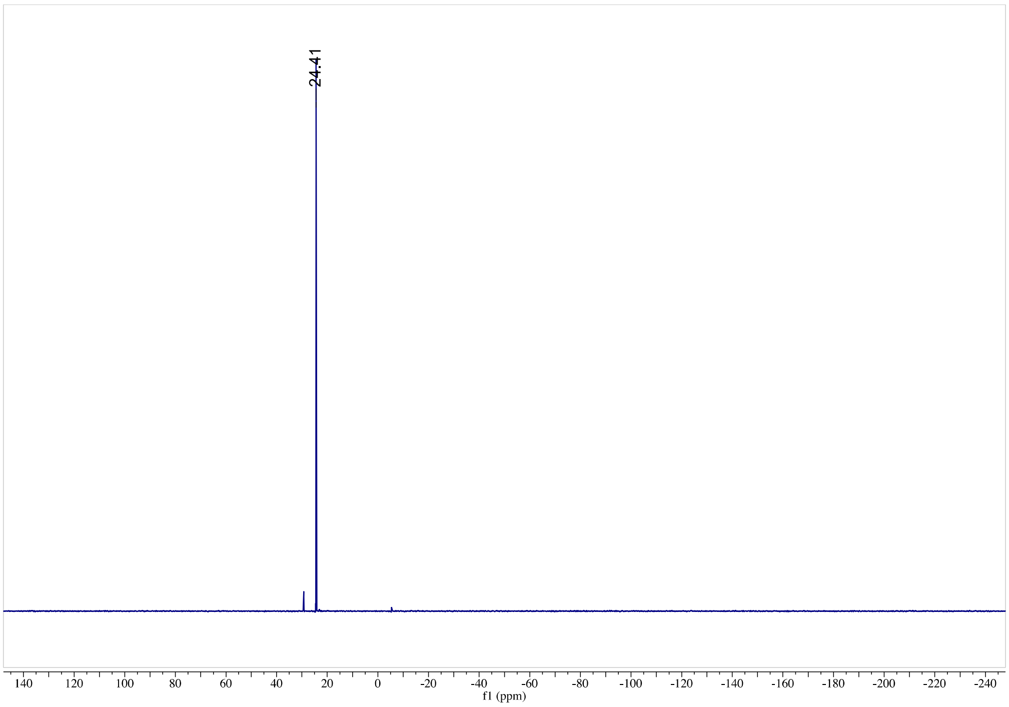

Figure S1.2.19. ^{31}P NMR spectrum (243 MHz, $CHCl_3$) of compound **5**.

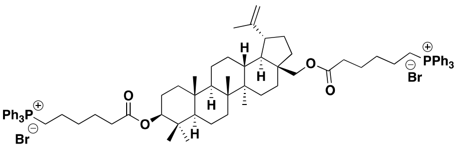

6

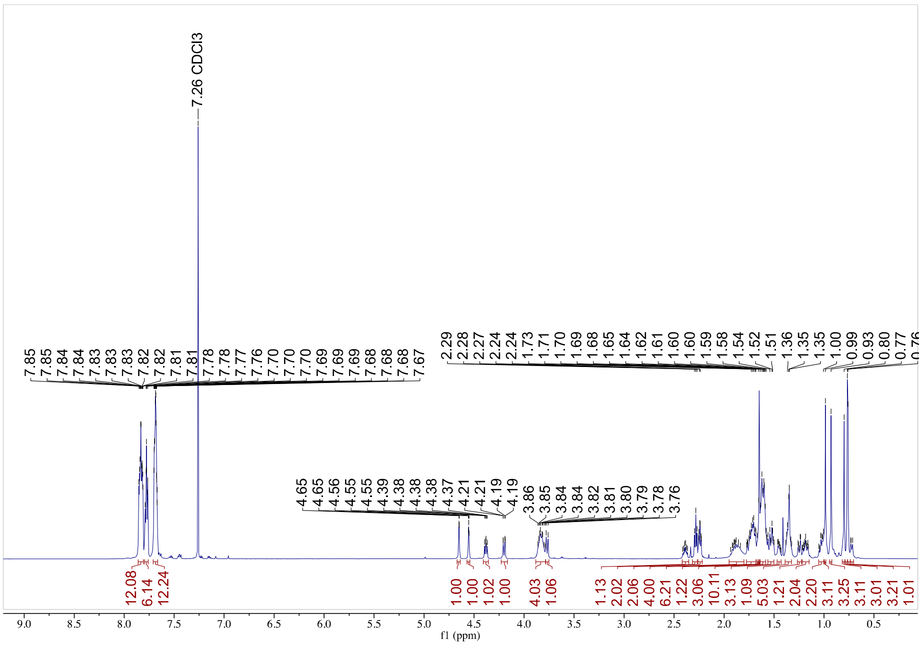

Figure S1.2.20. ^{1}H NMR spectrum (600 MHz, $CHCl_3$) of compound **6**.

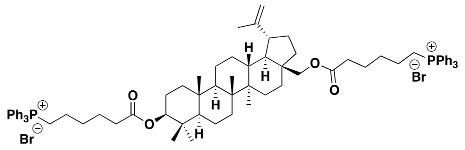

6

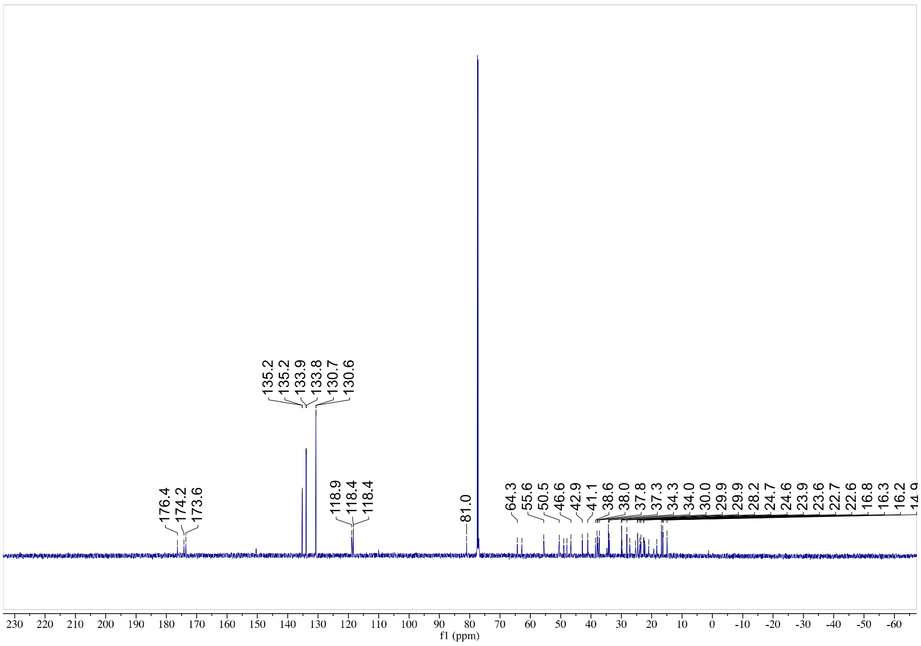

Figure S1.2.21. ^{13}C NMR spectrum (151 MHz, $CHCl_3$) of compound **6**.

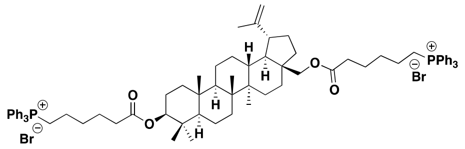

6

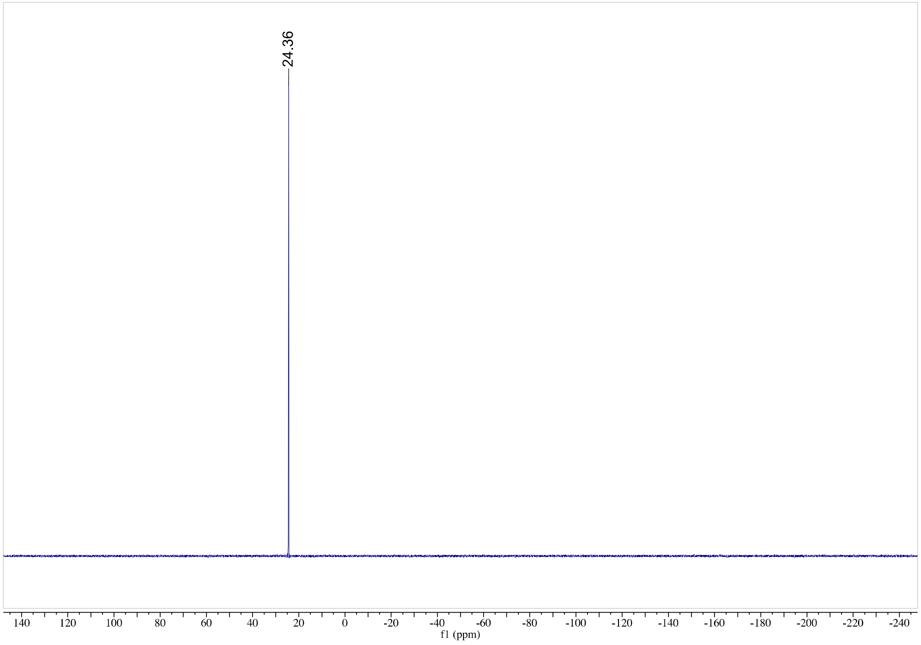

Figure S1.2.22. ^{31}P NMR spectrum (243 MHz, $CHCl_3$) of compound **6**.

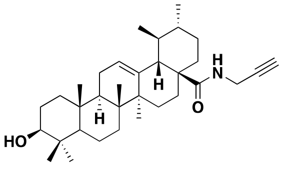

13

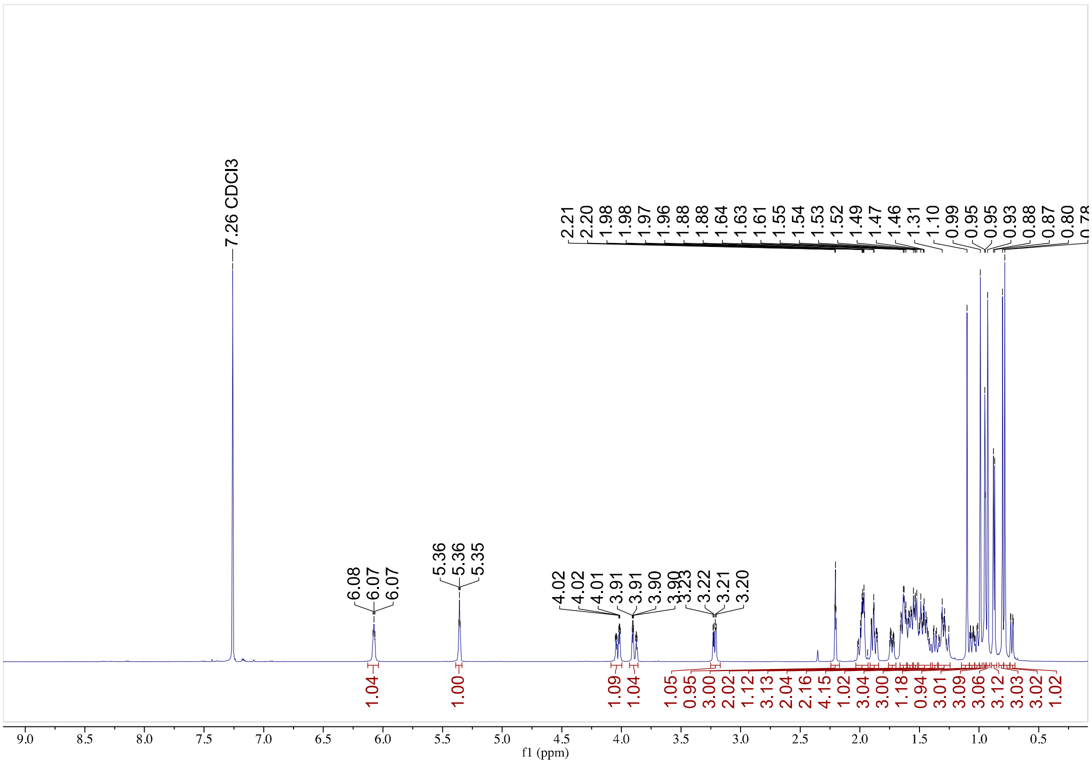

Figure S1.2.23. ^{1}H NMR spectrum (600 MHz, $CHCl_3$) of compound **13**.

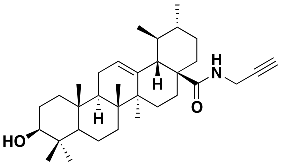

13

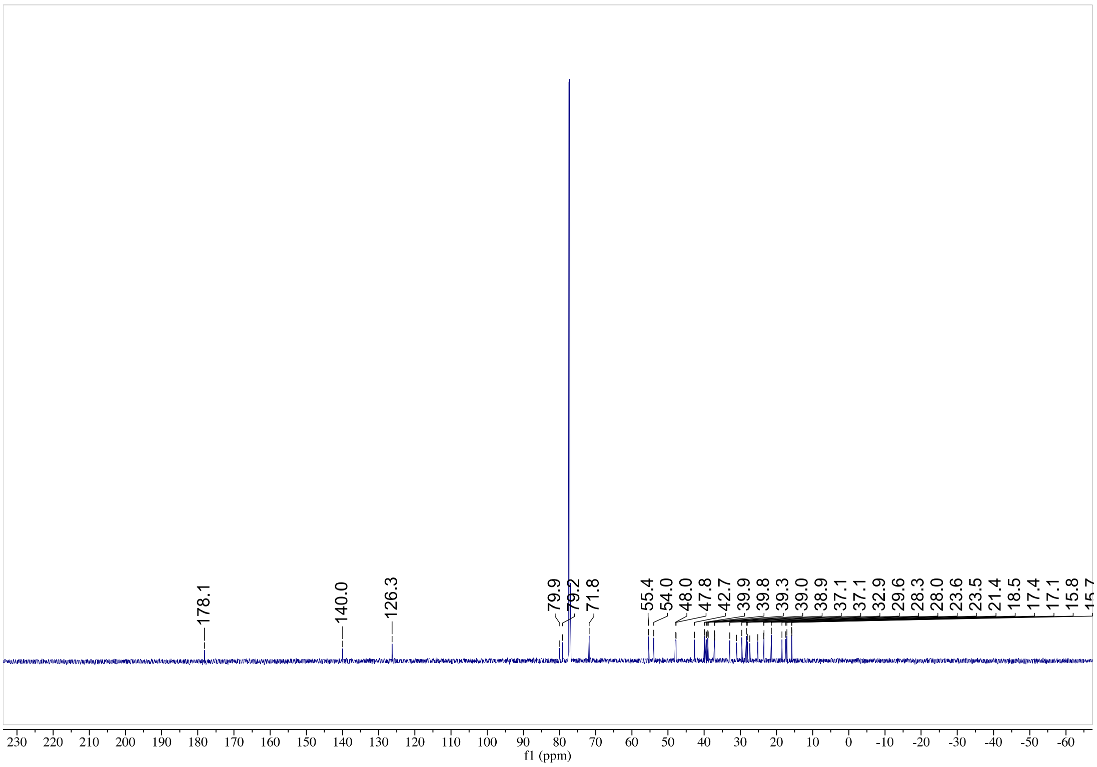

Figure S1.2.24. ^{13}C NMR spectrum (151 MHz, $CHCl_3$) of compound **13**.

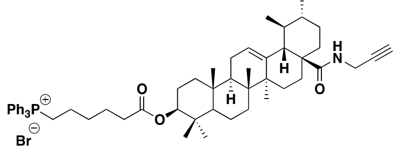

7

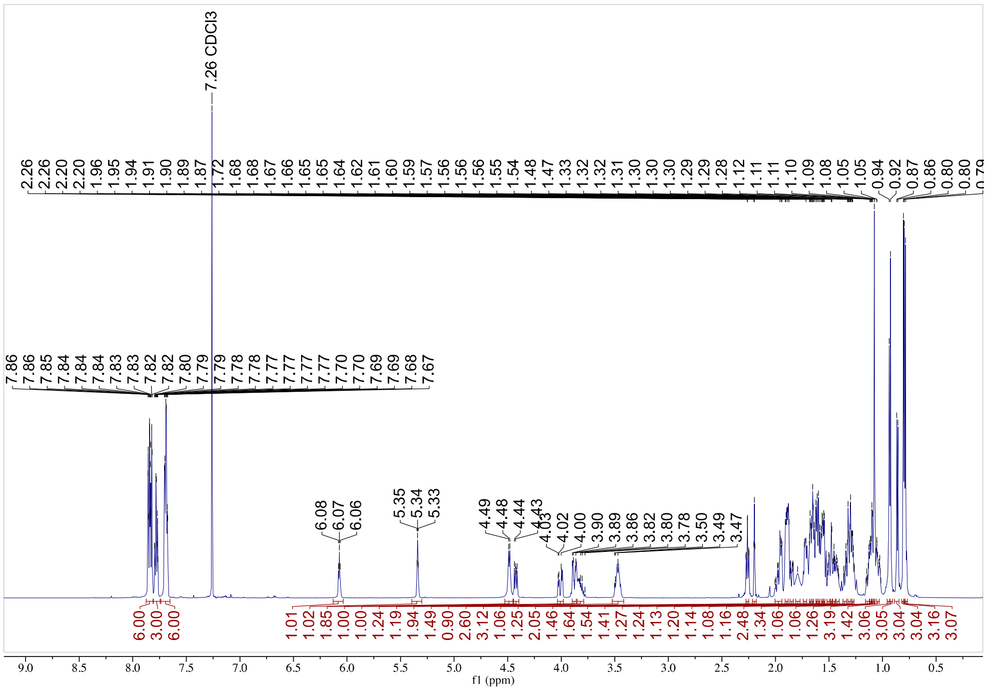

Figure S1.2.25. ^{1}H NMR spectrum (600 MHz, $CHCl_3$) of compound **7**.

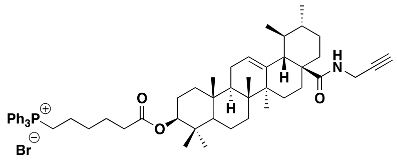

7

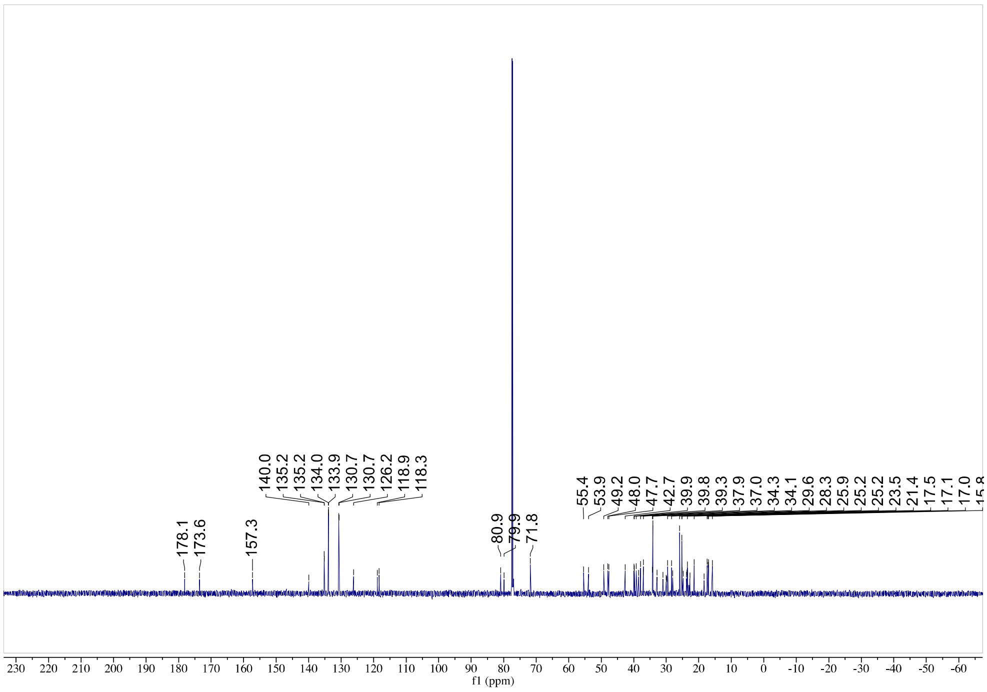

Figure S1.2.26. ^{13}C NMR spectrum (151 MHz, $CHCl_3$) of compound **7**.

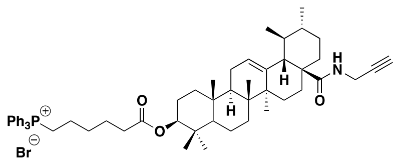

7

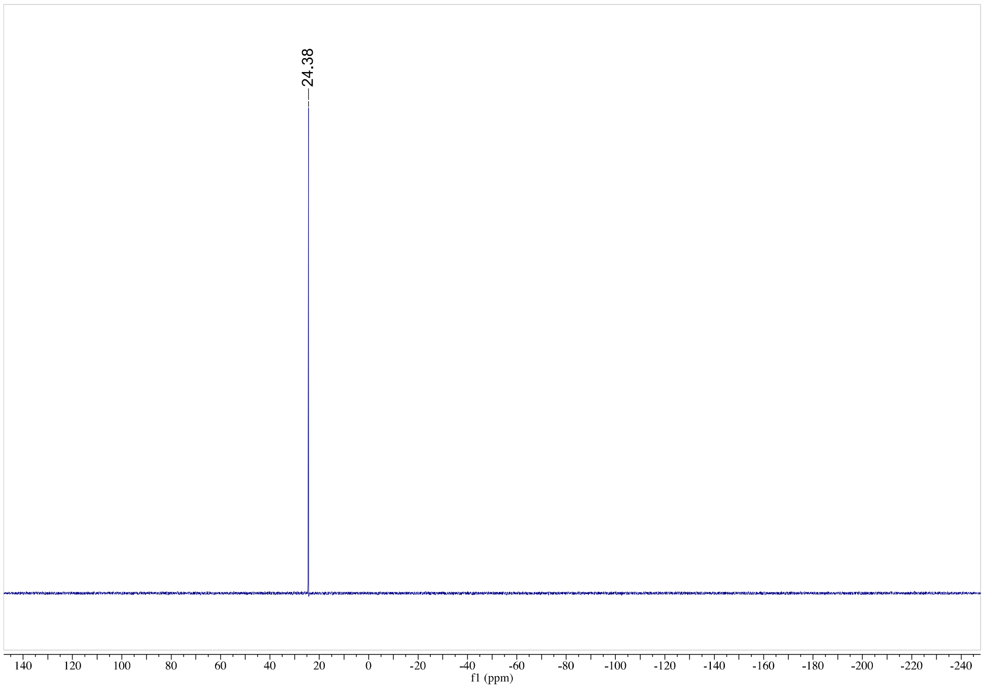

Figure S1.2.27. ^{31}P NMR spectrum (243 MHz, $CHCl_3$) of compound **7**.

Section S2. Antibacterial Properties of BET, BA, UA and Compounds **1-7** on Gram-positive and Gram-negative Clinical Isolates

Table S2.1. MICs of compounds **1-7**, BET, BA and UA against MDR clinical isolates of Gram-positive and Gram-negative species obtained from experiments conducted at least in triplicate.

Gram positive isolates	1	2	3	4	5	6	7	BET	BA	UA	R.A.
								MICs (μg/mL)			
S. aureus MRSA 18	64	>64	>64	4	8	8	2	>64	>64	64	256 (O)
S. epidermidis MRSE 22	32	>64	>64	4	4	8	2	>64	>64	32	128 (O)
E. faecalis VRE 1 *	64	>64	>64	16	8	16	4	>64	>64	4	256 (V); 64 (T)
E. faecium VRE 152 *	32	>64	>64	4	2	8	2	>64	>64	2	128 (V); 64 (T)
**Gram negative isolates**											
P. aeruginosa 259 °,°°,°°°	>64	>64	>64	>64	>64	>64	>64	>64	>64	>64	4 (M)
E. coli 477 KPC, °°	>64	>64	>64	>64	>64	>64	>64	>64	>64	>64	16 (M)
K. pneumoniae 376 KPC, °°	>64	>64	>64	>64	>64	>64	>64	>64	>64	>64	32(M)

RA = Reference antibiotics; * Resistant to teicoplanin; VRE = vancomycin-resistant enterococci; MRSA = methicillin resistant *S. aureus*; MRSE = methicillin resistant *S. epidermidis*; ° From patients with cystic fibrosis; °° resistant to carbapenems; °°° colistin resistant; KPC = *K. pneumoniae* carbapenemase-producing bacteria; R.A. = reference antibiotic; O = oxacillin; T = teicoplanin; V = vancomycin; BET = betulin; BA = betulinic acid; UA = ursolic acid; M = meropenem.

Table S2.2. Comparison between the antibacterial effects of BET derivatives **1**, **5**, **6**, BA derivative **4** and UA derivative **7**, with those of other BET, BA and UA derivatives previously reported.

Strains	BET derivatives (µg/mL)/(µM)			BA derivatives (µg/mL)/(µM)	UA derivatives (µg/mL)/(µM)	Refs
	**1**	**5**	**6**	**4**	**7**	
MRSA	64/66.4.	8/9.1.	8/6.1.	4/4.3.	2/2.1	This work
	**Others (µg/mL)**			**Others (µg/mL)**	**Others (µg/mL)**	
	128-24768/N.R.	128-24768/N.R.	128-24768/N.R.		>128/N.R.	[96], [111]
	250/N.R.**	250/N.R.**	250/N.R.**	544/N.R.	8/N.R.	[108], [98]
	102.4/N.R.	102.4/N.R.	102.4/N.R.		N.R./5-100	[109], [104],[112]
MRSE	32/33.2	4/4.6	8/6.1	4/4.3	2/2.1	This work
	**Others (µg/mL)**			**Others (µg/mL)**	**Others (µg/mL)**	
	N.F.	N.F.	N.F.	N.F.	>128/N.R.<br>N.R./3.1-25	[98], [112]
*E. faecalis**	64/66.4	8/9.1	16/12.2	16/17.2	4/4.2	This work
	**Others (µg/mL)**			**Others (µg/mL)**	**Others (µg/mL)**	
	102.4/N.R.	102.4/N.R.	102.4/N.R.	N.F.	64/N.R.<br>8/N.R.	[109],[98],[104]
*E. faecium**	32/33.2	2/2.3	8/6.1	4/4.3	2/2.1	This work
	**Others (µg/mL)**			**Others (µg/mL)**	**Others (µg/mL)**	
	N.F.	N.F.	N.F.	N.F.	32/N.R.	[98]

* VRE = vancomycin resistant; N.F. = not found; N.R. = not reported; ** ATCC. In red MIC values of compounds of this study.

Disclaimer/Publisher's Note: The statements, opinions and data contained in all publications are solely those of the individual author(s) and contributor(s) and not of MDPI and/or the editor(s). MDPI and/or the editor(s) disclaim responsibility for any injury to people or property resulting from any ideas, methods, instructions or products referred to in the content.
